# HLA dependency and possible clinical relevance of intrathecally synthesized anti-IgLON5 IgG4 in anti-IgLON5 disease

**DOI:** 10.3389/fimmu.2024.1376456

**Published:** 2024-05-16

**Authors:** Inga Koneczny, Stefan Macher, Markus Hutterer, Thomas Seifert-Held, Evelyn Berger-Sieczkowski, Morten Blaabjerg, Markus Breu, Jens Dreyhaupt, Livia Almeida Dutra, Marcus Erdler, Ingrid Fae, Gottfried Fischer, Florian Frommlet, Anna Heidbreder, Birgit Högl, Veronika Klose, Sigrid Klotz, Herburg Liendl, Mette S. Nissen, Jasmin Rahimi, Raphael Reinecke, Gerda Ricken, Ambra Stefani, Marie Süße, Helio A. G. Teive, Serge Weis, Thomas Berger, Lidia Sabater, Carles Gaig, Jan Lewerenz, Romana Höftberger

**Affiliations:** ^1^Division of Neuropathology and Neurochemistry, Department of Neurology, Medical University of Vienna, Vienna, Austria; ^2^Department of Neurology, Medical University of Vienna, Vienna, Austria; ^3^Department of Neurology, Johannes Kepler University Linz, Linz, Austria; ^4^Department of Neurology with Stroke Unit and Acute Geriatrics, Saint John of God Hospital Linz, Linz, Austria; ^5^Department of Neurology, Medical University of Graz, Graz, Austria; ^6^Department of Neurology, Landeskrankenhaus (LKH) Murtal, Standort Knittelfeld, Austria; ^7^Department of Neurology, Odense University Hospital, Odense, Denmark; ^8^Institute for Epidemiology and Medical Biometry, Ulm University, Ulm, Germany; ^9^Brain Institute, Hospital Israelita Albert Einstein, São Paulo, Brazil; ^10^Department of Neurology and Karl Landsteiner Institute for Neuroimmunological and Neurodegenerative Disorders Klinik Donaustadt, Vienna, Austria; ^11^Department of Blood Group Serology and Transfusion Medicine, Medical University of Vienna, Vienna, Austria; ^12^Center of Medical Data Science, Medical University of Vienna, Vienna, Austria; ^13^Department of Neurology, Medical University of Innsbruck, Innsbruck, Austria; ^14^Department of Neurology, University Hospital Ulm, Ulm, Germany; ^15^Department of Neurology, University Medicine Greifswald, Greifswald, Germany; ^16^Movement Disorders Unit, Neurology Service, Internal Medicine Department, Hospital de Clínicas, Federal University of Paraná, Curitiba, PR, Brazil; ^17^Division of Neuropathology, Department of Pathology and Molecular Pathology, Johannes Kepler University Linz, Linz, Austria; ^18^Comprehensive Center for Clinical Neurosciences and Mental Health, Medical University of Vienna, Vienna, Austria; ^19^Fundació de recerca clínic-Institut d’Investigacions Biomèdiques August Pi i Sunyer (FCRB-IDIBAPS), Caixa Research Institute (CRI), Universitat de Barcelona, Barcelona, Spain; ^20^Centro de Investigación Biomédica en Red de Enfermedades Raras (CIBERER), Madrid, Spain; ^21^Department of Neurology, Hospital Clínic, Barcelona, Spain

**Keywords:** IgLON5, IgG4, HLA, cerebrospinal fluid, intrathecal synthesis, therapy

## Abstract

**Background:**

Anti-IgLON5 disease is a rare chronic autoimmune disorder characterized by IgLON5 autoantibodies predominantly of the IgG4 subclass. Distinct pathogenic effects were described for anti-IgLON5 IgG1 and IgG4, however, with uncertain clinical relevance.

**Methods:**

IgLON5-specific IgG1-4 levels were measured in 46 sera and 20 cerebrospinal fluid (CSF) samples from 13 HLA-subtyped anti-IgLON5 disease patients (six females, seven males) using flow cytometry. Intervals between two consecutive serum or CSF samplings (31 and 10 intervals, respectively) were categorized with regard to the immunomodulatory treatment active at the end of the interval, changes of anti-IgLON5 IgG1 and IgG4 levels, and disease severity. Intrathecal anti-IgLON5 IgG4 synthesis (IS) was assessed using a quantitative method.

**Results:**

The median age at onset was 66 years (range: 54–75), disease duration 10 years (range: 15–156 months), and follow-up 25 months (range: 0–83). IgLON5-specific IgG4 predominance was observed in 38 of 46 (83%) serum and 11 of 20 (55%) CSF samples. Anti-IgLON5 IgG4 levels prior clinical improvement in CSF but not serum were significantly lower than in those prior stable/progressive disease. Compared to IgLON5 IgG4 levels in serum, CSF levels in HLA-DRB1*10:01 carriers were significantly higher than in non-carriers. Indeed, IgLON5-specific IgG4 IS was demonstrated not only in four of five HLA-DRB1*10:01 carriers but also in one non-carrier. Immunotherapy was associated with decreased anti-IgGLON5 IgG serum levels. In CSF, lower anti-IgLON5 IgG was associated with immunosuppressive treatments used in combination, that is, corticosteroids and/or azathioprine plus intravenous immunoglobulins or rituximab.

**Conclusion:**

Our findings might indicate that CSF IgLON5-specific IgG4 is frequently produced intrathecally, especially in HLA-DRB1*10:01 carriers. Intrathecally produced IgG4 may be clinically relevant. While many immunotherapies reduce serum IgLON5 IgG levels, more intense immunotherapies induce clinical improvement and may be able to target intrathecally produced anti-IgLON5 IgG. Further studies need to confirm whether anti-IgLON5 IgG4 IS is a suitable prognostic and predictive biomarker in anti-IgLON5 disease.

## Introduction

Anti-IgLON5 disease is a rare chronic autoimmune disorder hallmarked by antineuronal surface autoantibodies against IgLON5, an immunoglobulin superfamily cell-adhesion molecule with high expression in the central nervous system (CNS). The clinical presentation of anti-IgLON5 disease is quite heterogeneous with patients presenting with distinct rapid eye movement (REM) and non-REM parasomnias, obstructive sleep apnea and stridor, variable features of gait instability, movement disorders, and brainstem symptoms (1). Anti-IgLON5 disease is typically chronically progressive. Depending on which symptoms predominate, four different phenotypes have been delineated which are dominated by either (1): the sleep disorder with parasomnia and sleep apnea (2); bulbar dysfunction with dysarthria, dysphagia, sialorrhea, stridor, or acute respiratory insufficiency (3); a progressive supranuclear palsy (PSP)–like movement disorder; or (4) cognitive decline (1). Rare phenotypes mimic motor neuron disease or acute encephalitis (2, 3). The typically insidious clinical course and neuronal tau aggregates predominantly involving the brainstem and hypothalamus (4) suggest an underlying neurodegenerative process. However, tauopathy may be absent in some patients (5). In turn, a strong association with HLA-DRB1*10:01 and HLA-DQB1*05:01 alleles and the observations that IgLON5-specific IgG1 may cause IgLON5 protein internalization and subsequent cytoskeletal changes *in vitro* (6) point to a primary autoimmune process. Indeed, patients with anti-IgLON5 disease may benefit from early immunotherapy (7). The anti-IgLON5 antibodies are predominantly of the IgG4 subclass (followed by IgG1) in most patients (4). Anti-IgLON5 IgG1 induced irreversible IgLON5 internalization (8), whereas IgG4 interfered with IgLON5 protein interactions (9) *in vitro*. However, their individual contributions to disease *in vivo* and their prognostic/predictive relevance remain unclear.

Autoimmune encephalitis (AIE) with neuronal surface autoantibodies are heterogenous (10) but may be grouped according to the IgG1 or IgG4 predominance of their target-specific antineuronal antibodies (10). CSF inflammation in IgG4-predominant AIEs is typically less prominent than in IgG1-predominant AIEs (11). AIE associated with NMDAR antibodies is a prototypical IgG1-predominant AIE. Herein, prominent intrathecal synthesis (IS) of the total and target-specific IgG is well established (12). Surprisingly, in LGI1-antibody AIE (which is the most common IgG4-associated AIE), the otherwise non-inflammatory CSF contains many target-specific B cells. B cells and plasma cells also occur frequently in the largely non-inflammatory CSF in anti-IgLON5 disease (13, 14). Thus, target-specific IgG IS may also play a role in IgG4-predominant autoimmune disorders, including anti-IgLON5 disease. Of note, antigen-specific IgG IS is much more difficult to target therapeutically than systemic antibody production (15, 16).

Here, we longitudinally investigate the changes in individual anti-IgLON5 IgG subclass profiles in serum and CSF of 13 anti-IgLON5 disease patients in association with short- and long-term treatment follow-up. In addition, we established a method to quantify IS of anti-IgLON5 IgG4. Our aim was to delineate factors contributing to disease progression and characterize candidate biomarkers for therapeutic response.

## Methods

### Patients

Longitudinal serum and CSF of 13 patients with anti-IgLON5 disease were included in this study. The samples were referred to the Division of Neuropathology and Neurochemistry at the Department of Neurology, Medical University of Vienna for diagnostic testing between October 2014 and December 2022. The clinical information was obtained by the investigators. Four patients were previously published (17–21). The disease severity was estimated as functional impairment using the modified Rankin scale (mRS) at the different sample time points.

### Flow cytometry analysis of IgG subclasses

The analysis of IgG subclasses was adapted from our previously published method (22, 23). Briefly, HEK293T cells were transfected with plasmid DNA encoding full-length human IgLON5 fused to Turbo GFP (tGFP, derived from Origene, Rockville, Maryland, USA, RG22549). Each 2 × 10^5^ cells were incubated with a 1:40 dilution of patient serum or 1:10 dilution of CSF in incubation medium (DMEM supplemented with 1% penicillin/streptomycin, 1% BSA, 20mM HEPES, and 10% donkey serum) for 20 min at 4°C. The cells then were washed, fixed for 10 min using 4% paraformaldehyde, washed, and incubated with 1:500 biotinylated mouse anti-human IgG1 (B6775), IgG2 (B3398), IgG3 (B3523), or IgG4 (B3648, all by Sigma-Aldrich, St Louis, Missouri, USA) in incubation medium for 30 min at 4°C. The cells were again washed, and bound biotinylated IgG was visualized by incubating cells with Cy3-streptavidin (434315, Invitrogen, Waltham, Massachusetts, USA) diluted 1:750 in incubation medium for 30 min at 4°C in the dark. The cells were washed and resuspended in phosphate-buffered saline with 2.5 mM ethylenediaminetetraacetic acid, pH 8.0 before analysis with a Cytoflex LX flow cytometer (Beckman Coulter Brea, California, USA). tGFP-positive and -negative cells were gated, respectively, and their median fluorescence intensity (MFI) for Cy3 was measured in the phytoerythrin channel (**Supplementary Methods Figure S1**), the value of tGFP-negative cells was subtracted from tGFP-positive cells to account for unspecific binding of antibodies to the cells (ΔMFI). Depending on serum/CSF availability, the measurements were repeated in independent experiments (*N* = 1–5). Four reference samples were measured in every experiment and used to normalize the results of individual experiments (**Supplementary Methods**). These assay conditions resulted in a largely linear correlation of ΔMFI with antibody levels. At very high levels, ΔMFI reached the nonlinear range (**Supplementary Methods**). While technical limitations (the use of different subclass-specific antibodies) did not permit the calculation of absolute percentages of anti-IgLON5 IgG1, 2, 3, and 4 and total anti-IgLON5 IgG, the sum of anti-IgLON5 IgG1 + 2+3 + 4 ΔMFI was used as a surrogate marker for total anti-IgLON5 IgG (“total” anti-IgLON5 IgG), which was used to determine relative percentages of anti-IgLON5-IgG1, 2, 3, and 4. IgG4 predominance was defined as anti-IgLON5 IgG4 exceeding 66% of “total” anti-IgLON5 IgG. Anti-IgLON5 IgG4/IgG1 ratios were calculated for every sample. All analyses were made for anti-IgLON5 IgG1-4. Thus, all references to IgG1-4 always refer to anti-IgLON5 IgG1-4.

### Interval analysis

**Figure 1:**
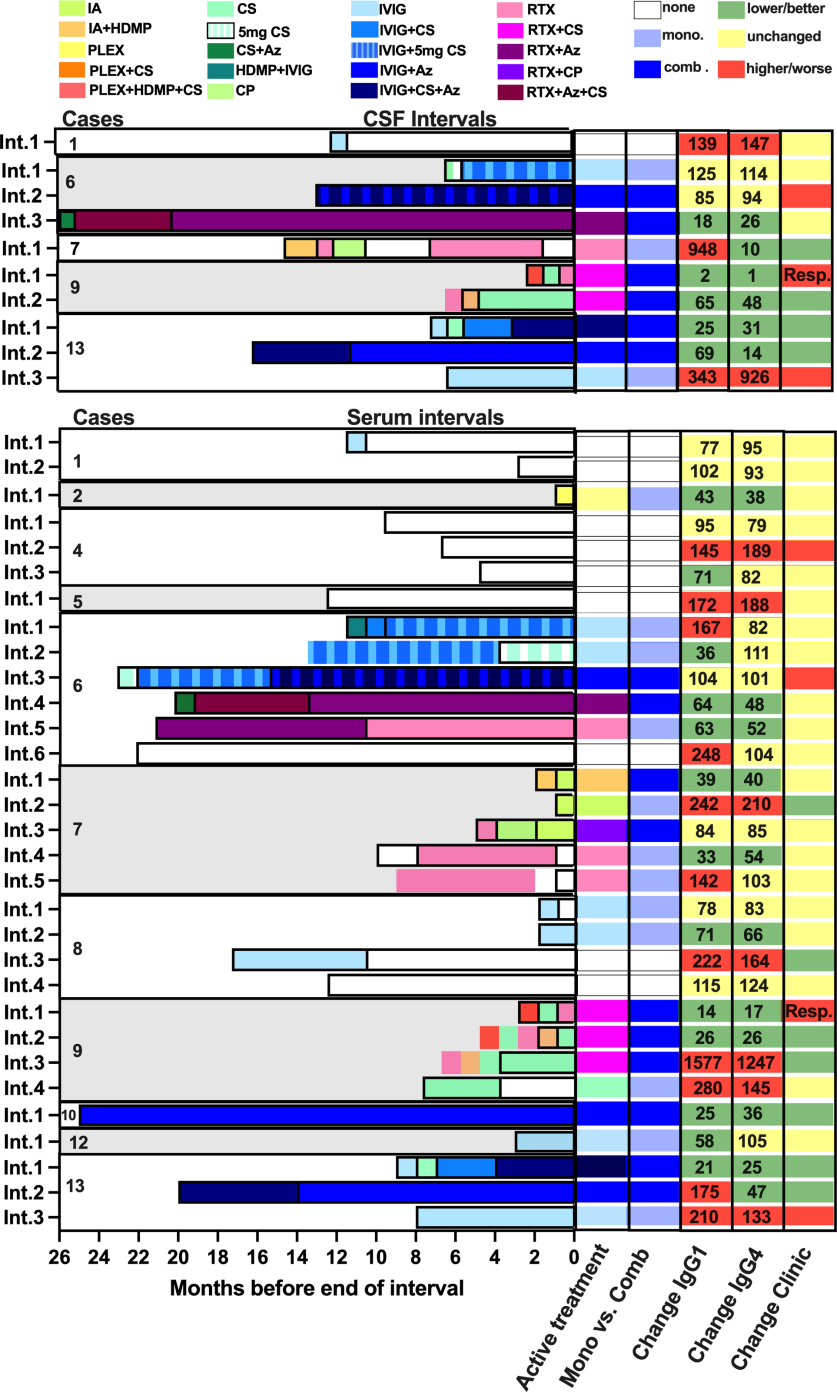
Graphical summary of serum and cerebral spinal fluid intervals including immunomodulatory treatments, changes in anti-IgLON5 IgG1 and IgG4 levels, as well as clinical changes. Treatments were color-coded as indicated. The last active treatment was assessed as described in the Methods sections. Two long-active treatment in parallel (with corticosteroids >5 mg/day) were categorized as combination treatment. The numbers in the columns for changes in IgG1 and IgG4 indicate the percentage change at the end of each interval compared to the start. Resp = patient responded well to rituximab 1 month after first treatment, but the interval terminated 1 week before manifestation of clinical improvement.

Each interval flanked by either two subsequent serum or CSF samplings was characterized in four dimensions: (1) duration, (2) immunosuppressive treatment regimen at the end of the interval, (3) changes in disease severity, and (4) anti-IgLON5 IgG levels. We defined the following time spans for the treatment effects, which include one additional month as buffer: 4 months for intravenous immunoglobulins (IVIG) (24), 7 months for rituximab, 4 months for corticosteroids >5 mg, 5 months for cyclophosphamide, 5 months for azathioprine. For acute therapies, immunoadsorption (IA) and plasmapheresis were considered active for 1 month (**Figure 1**). Clinical improvement was defined as a decrease and clinical worsening as an increase in the mRS. In mildy affected patients (mRS always 1), descriptions of clinical improvements or worsening were taken into account. A >25% increase in antibody levels during the interval was defined as increased, a change of ±25% as unchanged, and >25% decrease as decreased levels. We defined IgG4/IgG1 ratio during sampling intervals as lower (>20% less), stable (0 ± 20%), or increased (>20% higher) ratios.

### HLA genotyping

Genotyping of HLA-DRB1 and -DQB1 was performed as described (25). We used HLA-genotyping data from the 11 Austrian patients (**Supplementary Table S3**) and HLA data from a cohort of 200 Austrian individuals from the database of the Allele Frequency Network (26) to calculate the relative frequency of distinct HLA alleles as odds ratio (OR, **Supplementary Table S4**).

### Intrathecal IgLON5 antibody synthesis

Anti-IgLON5 IgG4 IS was assessed in anti-IgLON5-disease CSF/serum pairs with routine data for the total CSF/serum IgG ratio (Q_IgG_) and CSF/serum albumin ratio (Q_Alb_) available. No IS of total IgG was reported for the serum/CSF pairs utilized. To calculate CSF/serum ratios, data points need to be in the absolutely linear range of the assay, and matrix effects must be absent. To generate suitable raw data, anti-IgLON5 IgG4 levels were measured in sera and CSF following dilution to identical IgG concentrations, to ensure ΔMFI intensities in a similar range in the absence of IS and strictly within the linear range of the assay (see **Supplementary Methods**). The CSF/serum ΔMFI intensity ratio obtained by this approach is identical to the CSF/serum antibody index (AI) as reported by Reiber and Lange (27). The CSF/serum anti-IgLON5 IgG4 ratio was then obtained by multiplying the CSF and serum ΔMFI by the respective dilution factors. Z scores for anti-IgLON5 IgG4 IS and total IgG IS were calculated as described recently (28). Control CSF/serum pairs without IS (“control AI 1,” IgLON5-antibody-negative CSF/serum pairs spiked with high-titer IgLON5-IgG4 sera to obtain a Q_IgLON5-IgG4_ identical to the Q_IgG_) and 10-fold IS (“control AI 10,” IgLON5-antibody-negative CSF/serum pairs spiked with high-titer IgLON5-IgG4 sera to obtain a Q_IgLON5-IgG4_ 10-fold higher than the Q_IgG_) were used to successfully validate the assay (**Supplementary Methods**).

### Statistical analysis

The OR and 95% confidence intervals (95% CI) for individual HLA alleles of Austrian patients were calculated using Episheet^1^ (29). Unless stated otherwise, descriptive statistics of ordinal- and interval-scaled variables are given as median and interquartile range (IQR). The statistical analysis was done with GraphPad Prism 9.0 software, using either Fisher’s exact test, Mann–Whitney, Kruskal–Wallis test or linear regression with Pearson correlation as appropriate. Statistical significance was determined as *p* ≤ 0.05. Due to the low number of patients, data points and the retrospective nature, the statistical analysis had to be categorized as exploratory and merely hypothesis-generating. Consequently, a one-sided testing strategy was applied if not stated otherwise.

### Standard protocol approvals, registrations, and patient consents

The study was approved by the Ethics Committee of the Medical University of Vienna (EK 1442/2017, 1636/19, and 1123/15).

## Results

### Patients

Twenty CSF and 46 serum samples (in total 66 samples) were available from 13 patients with positive results in the IgLON5 cell-based assay (six females, seven males, 11 from Austria, one from Denmark, and one from Germany). The median age at onset was 66 years (range: 54–75) with a follow-up time of 25 months (range: 0–83). Longitudinal sampling was available from 11 patients with a median number of four serum samples (range: 0–7) and one CSF sample (range: 0–4). Among these, five patients with matching serum/CSF pairs with routine data for Q_Alb_ and Q_IgG_ were still available after initial measurements. The disease duration between disease onset and the last serum/CSF sample analyzed was 10 years (range: 1.25–13 years).

Nine of the 13 patients (69%) exhibited one of the phenotypes characterized by Gaig et al. (1). However, only one patient (8%) presented with the prototypical sleep disorder. A bulbar syndrome found in four patients (31%) was most common. A PSP-like phenotype was also common (3 of 13, 23%). One patient (8%) presented with a cognitive phenotype. Four patients (31%) could not be classified according to Gaig et al. (1). Two (15%) predominantly exhibited peripheral hyperexcitability. One of the two remaining patients’ phenotype clinically resembled Parkinson’s disease in combination with peripheral hyperexcitability. The other remaining patient showed gait instability, mild cognitive impairment, sensory hyperexcitability as well as transient choreoathetosis of the left arm (**Supplementary Tables S1/S2**, see Appendix for detailed case descriptions).

Median mRS at first presentation was 3 (range: 1–5). Three (23%) patients improved by at least one mRS point during follow-up, six (46%) worsened and died during follow-up after a disease duration of 124 (120–147) months compared to seven (54%) surviving patients with a disease duration at last follow-up of 85 (48–128) months (*p* = 0.08). The patients who died had significantly higher mRS (median: 3.5, range: 3–5) at time of diagnosis than surviving patients (median: 2, range: 1–3; *p* = 0.006, **Supplementary Figure S1**). Nine of 13 (69%) patients received eight different therapeutic regimens [IVIG, plasmapheresis (PLEX), oral corticosteroids (CS), intravenous high-dose methylprednisolone, azathioprine (Aza), rituximab (RTX), cyclophosphamide (CP), immunoadsorption (IA) alone or in combination]. Four patients (31%) carried both HLA-DRB1*10:01 and HLA-DQB1*05:01 risk alleles, six (46%) carried one risk allele (one HLA-DRB1*10:01 only, five HLA-DQB1*05:01 only), while three (23%) carried none (**Supplementary Table S3**). Compared to an Austrian reference population, the allele frequency for both HLA-DRB1*10:01 and HLA-DQB1*05:01 was significantly increased in the 11 Austrian patients [OR (95%CI): HLA-DRB1*10:01 75 (5–3933), *p* < 0.001; HLA-DQB1*05:01 8 (2–51), *p* < 0.01, **Supplementary Table S4**]. Presence of the HLA-DRB1*10:01 risk allele did not affect age of onset or sex distribution (**Supplementary Figure S2A**). However, DRB1*10:01-negative patients at clinical presentation tended to be slightly less functionally impaired [median mRS (IQR): DRB1*10:01-negative 2.5 (2.0–3.0), DRB1*10:01-positive 3.0 (3.0–4.5); *p* = 0.1974] associated with a tendency to more frequent clinical stability [negative: five of eight (63%), positive: one of five (20%); *p* = 0.1795, **Supplementary Figure S2B**]; however, these effects were not statistically significant. All three patients with a PSP-like phenotype were DRB1*10:01-negative [PSP-like/total with specificDRB1*10:01 carrier status: negative 3/8 (38%), positive 0/5, *p* = 0.0435], two other DRB1*10:01-negative cases presented with atypical phenotypes with peripheral hyperexcitability (one resembling Parkinson’s disease, and the other non-classifiable, **Supplementary Figure S2C** and **Supplementary Tables S1/S2**). Interestingly, median CSF cell count was sevenfold higher in DRB1*10:01-positive compared to negative cases [positive: 7 (2.5–36) leukocytes/µl; negative: 1 (0–3.5) leukocytes/µl; *p* = 0.0101, **Supplementary Figure 2D**].

### Changes in CSF anti-IgLON5 IgG4 coincide with changes in disease severity

**Figure 2:**
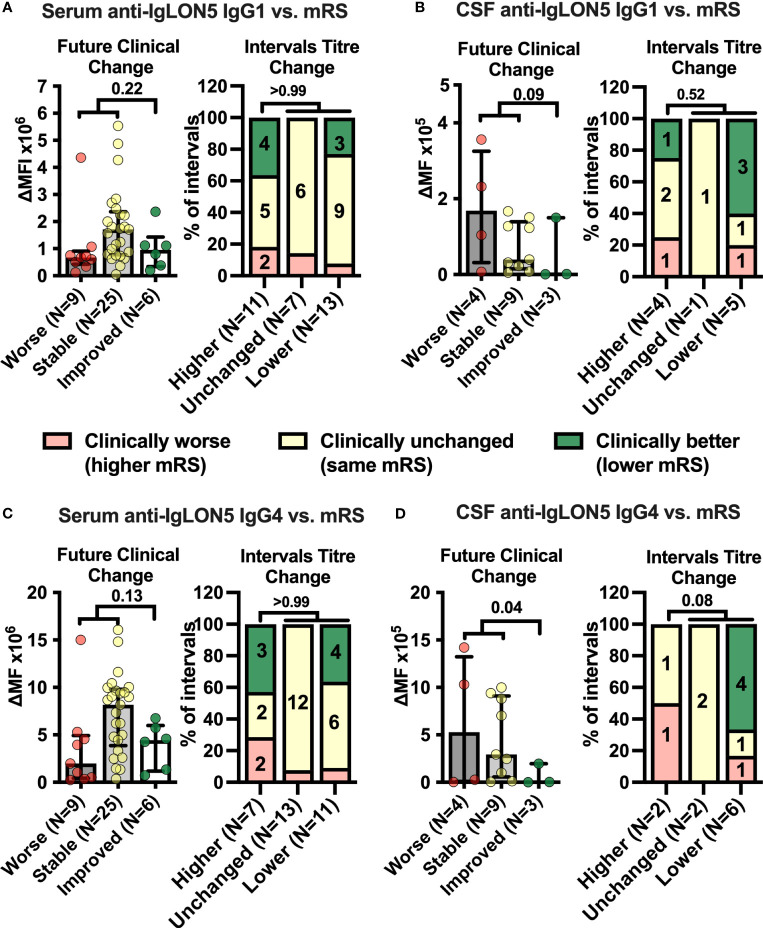
Cerebrospinal fluid anti-IgLON5 IgG1 and IgG4 levels and their changes over time correspond better with disease severity than serum levels. **(A–D)** Left graphs: IgLON5 IgG1 (**A, B** left) or IgG4 (**C, D** left) levels (bars + error bars = mean ± SEM) in serum (**A, C** left) and cerebrospinal fluid (CSF, **B, D** left) measured by flow cytometry and expressed as mean fluorescence intensity above background (ΔMFI) as in **Figure 1** were grouped according to future clinical change at the next follow-up visit categorized as clinically worsened (≥ +1 mRS point, red), unchanged (mRS unchanged, yellow) or improved (≤ −1 mRS point, green). If the mRS was 1 (no functional impairment), changes of reported symptoms were used to categorize intervals. **(A–D)** Right graphs: The clinical change between two consecutive serum (A, C, right) or CSF (B, D, right) sampling intervals was categorized as clinically worse (≥ +1 mRS point, or worsening of symptoms if mRS = 1, red), unchanged (mRS unchanged, or unchanged symptoms if mRS =1, yellow) or improved (≤ −1 mRS point, or improvement of symptoms if mRS =1, green) and changes of anti-IgLON5 IgG1 (**A, B** right) or IgG4 (**C, D** right) levels during these intervals were categorized as higher (+ ≥ 20%), unchanged (± 20%), or lower (−≥ 20%). Bars show the percentage of intervals with one of the three clinical change categories when grouped according to the antibody change category. Numbers in stacked bar graphs indicate the number of intervals. Numbers in column titles indicate the numbers of measurements (left graphs) or intervals (right graphs). For statistical analysis, data were dichotomized into clinically worse/stable versus improved (left graphs) or higher levels versus unchanged or lower levels (right graphs) before testing using one-tailed Mann–Whitney *U* (left graphs) or Fisher’s exact tests (right graphs). The *p*-values are indicated.

Next, we assessed anti-IgLON5 IgG1-4 levels in serum and CSF (**Supplementary Figures S3/S4**). The relative variability in serum anti-IgLON5 IgG1 was higher than for IgG4 (**Supplementary Figure S5**). Anti-IgLON5 IgG4 predominated in 38 of 46 (83%) serum, but only 11 of 20 (55%) CSF samples (*p* = 0.022), IgG1 in one of 46 (2%) serum and one of 20 (5%) CSF samples. Interestingly, IgG4 predominance of anti-IgLON5 IgG was due to higher anti-IgLON5 IgG4 levels but not lower anti-IgLON5 IgG1 levels in both serum and CSF (**Supplementary Figure S6**). Then, we analyzed whether either anti-IgLON5 IgG1 and 4 levels in serum and CSF preceded future clinical changes or whether changes in antibody levels were paralleled by changes in symptoms severity in the various intervals between CSF and serum samplings with different treatments considered active at the end ot the interval (**Figure 1**). In contrast to serum (**Figures 2A/2C**, left panels), CSF anti-IgLON5 IgG1 tended to be and IgG4 levels were significantly lower prior to improvement compared to stable or worsening disease (IgG1: *p* = 0.09, IgG4: *p* = 0.04, **Figures 2B, D**, left panels). During the intervals, no association with changes in serum anti-IgLON5 IgG1/4 levels nor CSF anti-IgLON5 IgG1 levels was found (**Figures 2A–C**, right panels). However, in four of six (66%) intervals with decreased CSF anti-IgLON5 IgG4 levels clinical improvement was noted but, in none of the intervals in stable or increased CSF anti-IgON5 IgG4 levels, a finding not statistically significant due to the low number of data points (*p* = 0.08, **Figure 2D**, right panel). Of note, no systematic changes of interval length correlated with changes in IgG4, IgG1, IgG4/IgG1 ratio, or functional impairment (**Supplementary Figure S7**). Q_Alb_, available for seven of 13 (54%) measurements preceding stable disease or worsening and two of three (67%) preceding improvement, was not lower in the group experiencing future improvement than in those with less good outcome (**Supplementary Figure S8**). Thus, it seems highly unlikely that the low CSF IgG4 levels prior to improvement are explained by better blood/CSF-barrier function resulting in less filtration of blood anti-IgLON5 IgG4 into the CSF.

### CSF anti-IgLON5 IgG4 is synthesized intrathecally especially in HLA-DRB*10:01 carriers

**Figure 3:**
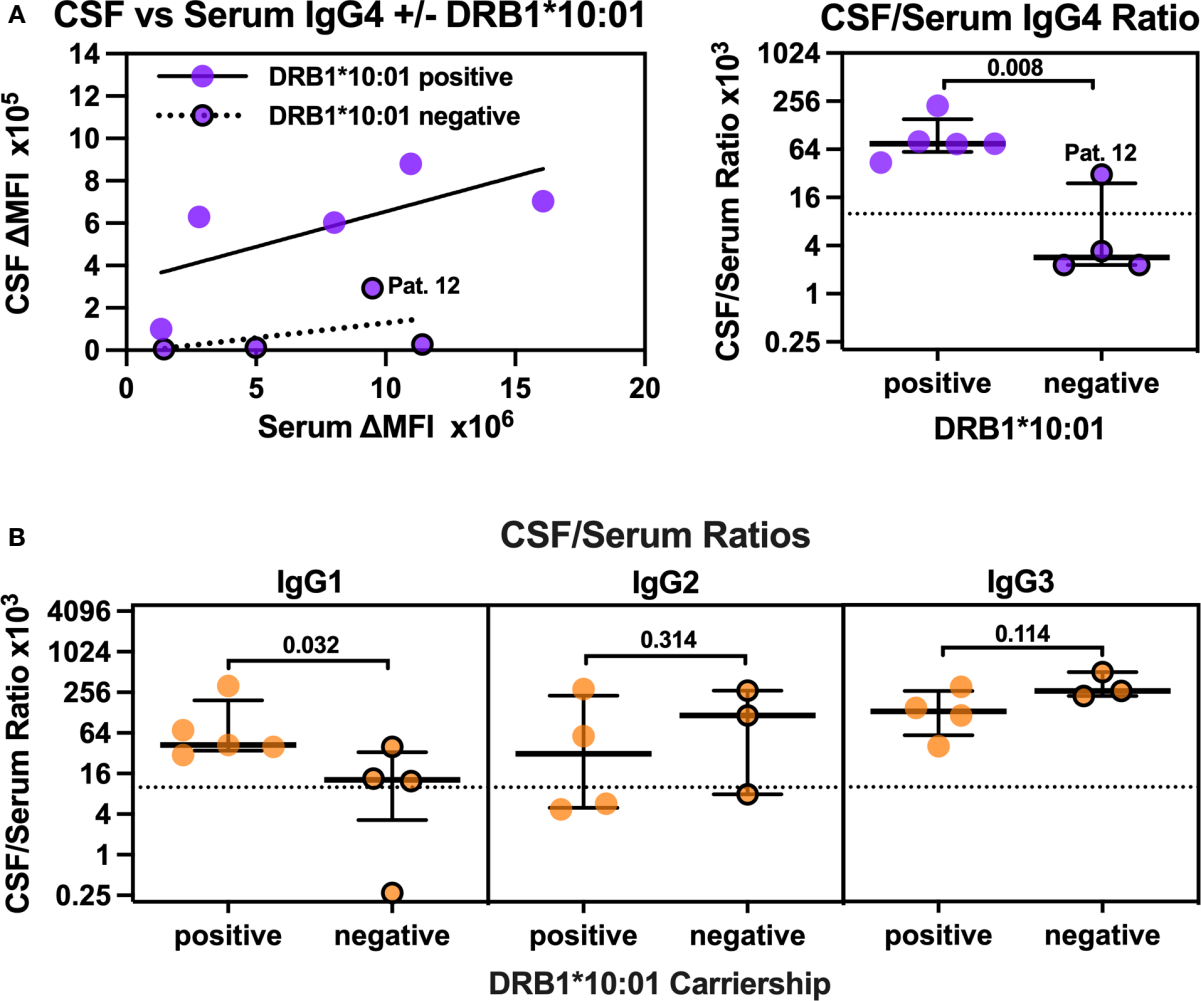
CSF/Serum ratios for anti-IgLON5 IgG4 but also IgG1 are higher in DRB1*10:01 carriers compared to non-carriers. (**A**, left panel) The mean fluorescence intensity above background (ΔMFI) for anti-IgLON5 IgG4 in CSF was plotted against serum anti-IgLON5 IgG4 ΔMFI separatedly for DRB1*10:01 carriers (circles without borders) and non-carriers (circles with black borders). The results of linear regression (carriers: *R*^2 =^ 0.47, *p* = 0.1984, non-carriers *R*^2^ 0.21, *p* = 0.5466) and the non-carrier with the higher CSF/serum ratio (patient 12) are indicated. (**A**, right panel, **B**): CSF/serum ratios for anti-IgLON4 IgG4 (**A**, right panel), IgG1–3 **(B)** of DRB1*10:01 carriers compared to non-carriers. Statistical analysis in the right panel of **(A, B)** was performed using one-sided Mann–Whitney *U* tests. The *p*-values are indicated. A comparison of anti-IgLON5 IgG4 CSF/serum ratios in association with of DRB1*10:01 and DQB1*05:01 carriership can be found in **Supplementary Figure S13**.

Plotting CSF against serum anti-IgLON5 IgG4 levels in all first CSF/serum pairs showed considerably higher relative CSF anti-IgLON5 levels in patients carrying the DRB1*10:01 allele compared to non-carriers (**Figure 3A**, left panel). When filtrated into the CSF from plasma, IgG levels are usually 100- to 1,000-fold lower compared to serum levels corresponding to CSF-to-serum ratios of 1–10 × 10^−3^ (30). CSF levels in three of four DRB1*10:01-negative patients were 292- to 433-fold lower than serum levels (**Supplementary Table S2**), thus, in the expected range when explained by filtration into the CSF only. CSF anti-IgLON5 IgG4 in the fourth non-carrier, patient 12, was only 32-fold lower. In the five DRB1*10:01 carriers, CSF anti-IgLON5 levels were only fourfold to 23-fold lower than the respective serum levels. This resulted in 26-fold higher CSF/serum IgG4 ratios in DRB1*10:01 carriers [75 (59–153) × 10^−3^] compared to non-carriers [2.9 (2.3–24) × 10^−3^, *p* = 0.008, **Figure 3A**, right panel]. Of note, this effect seems to be independent of a simultaneous DQB1*05:01 carriership (**Supplementary Figure S9**). Thus, we hypothesized that, especially in DRB1*10:01 carriers, anti-IgLON5 IgG4 may be synthesized intrathecally. Of note, a similar, but less robust, threefold increase in the CSF/serum anti-IgLON5 IgG1 ratio was found in DRB1*10:01 carriers (**Figure 3B**, left panel), while anti-IgLON5 IgG2 and IgG3 CSF/serum ratios tended to be higher in non-carriers (**Figure 3B**, middle/right panels). These findings strongly suggest anti-IgLON5 IgG4 IS, especially in DRB1*10:01 carriers.

To exactly quantify the IS of anti-IgLON5 IgG4 (zQ_IgLON5-IgG4_) in comparison to total IgG IS (zQ_Total IgG_) in CSF/serum pairs with available data for Q_Alb_ and Q_IgG_ and remaining sample volume, we employed an innovative and sensitive strategy using IS *z* scores (see **Supplementary Methods**). Overall, a strong correlation the zQ_IgLON5-IgG4_ with the anti-IgLON5 IgG4 serum-CSF ratio was observed (**Supplementary Figure S10**), indicating that the mere ratios analyzed for their dependency on the HLA genotype (**Figure 3**) represent a good surrogate parameter of anti-IgLON5 IgG4 IS.

**Figure 4:**
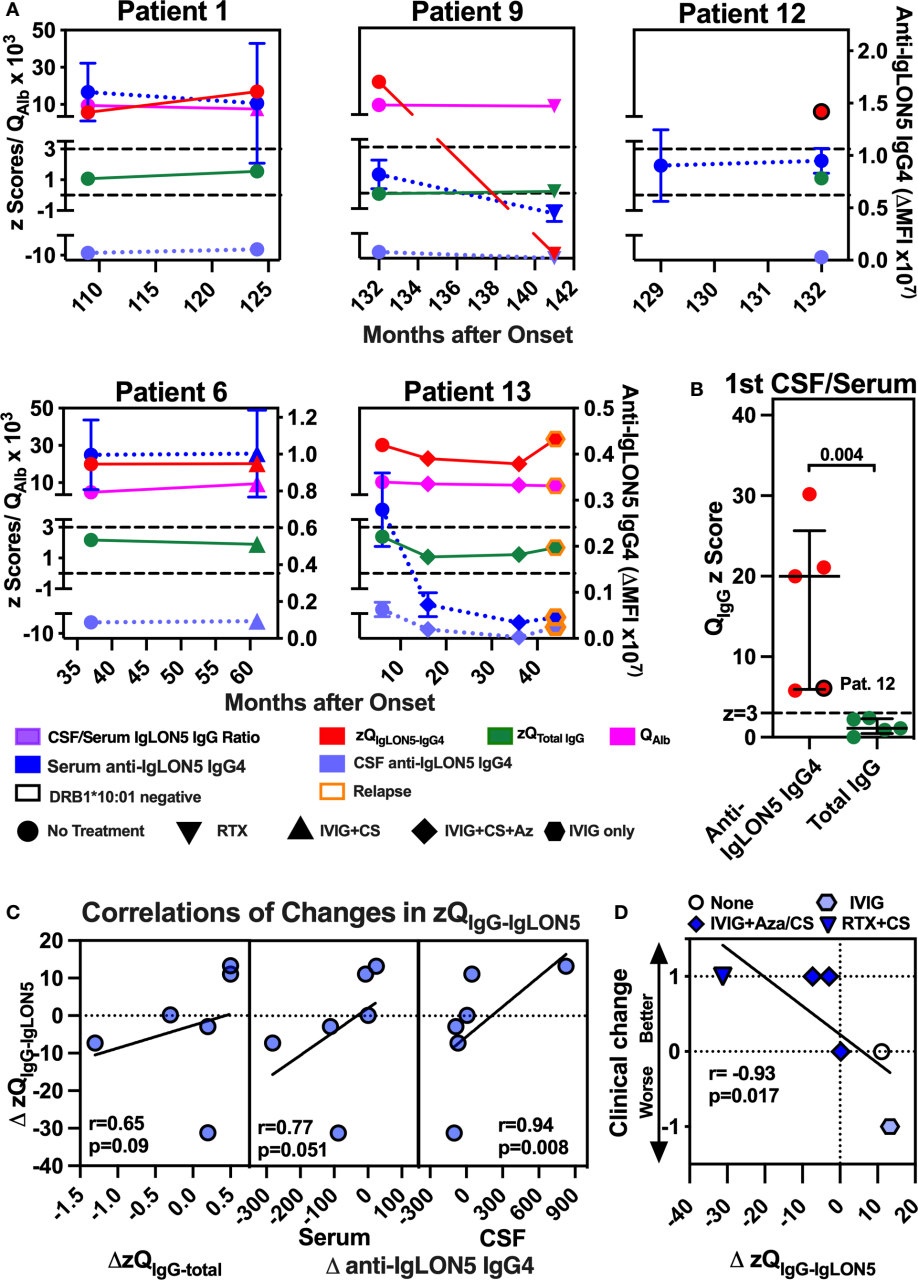
Robust intrathecal synthesis of anti-IgLON5 IgG4 was observed in five of five patients, and change in clinical severity and CSF IgLON5 IgG4 correlate with changes in the IgLON5-IgG4–specific IS (zQIgG-IgLON5). (**A**, left panel) The mean fluorescence intensity above background (ΔMFI) for anti-IgLON5 IgG4 in CSF of each first CSF/serum pair available was plotted against the anti-IgLON5 IgG4 ΔMFI in serum for five DRB1*10:01 carriers (no border) and four non-carriers (black border). Patient 12 is indicated. The lines represent simple linear regression. (**A**, right panel) CSF/serum ratios of DRB1*10:01 carriers compared to non-carriers. **(B)** Precise quantification of intrathecal anti-IgLON5 IgG4 synthesis using CSF/serum pairs with corresponding data for CSF/serum IgG (Q_IgG_) and albumin ratio (Q_Alb_) from patients 1, 6, 9, 12, and 13 still available following the initial measurements. Results from repeat flow cytometry performed after dilution to equal concentrations of total IgG in CSF and serum were converted to z-scores for anti-IgLON5 IgG4 (zQ_IgLON5-IgG4_) _IgG4_ and total IgG (zQ_Total IgG_) as described in the methods and **Supplementary Methods**. Results for zQ_IgLON5-IgG4_ (red) and zQ_Total IgG_ (green) were plotted against the disease duration using the left *y*-axis. The corresponding Q_Alb_ (pink, left *y*-axis) and serum and CSF anti-IgLON5 IgG4 mean ± SEM levels dark blue and light blue, respectively, right *y*-axis) are shown for comparison. The cutoff above which definite intrathecal synthesis (*z* = 3) can be assumed and z-score without intrathecal synthesis (*z* = 0) are indicated as black dashed lines. A black border indicates DRB*10:01 non-carriers, orange border clinical relapse. Circle: no treatment in the preceeding interval, upright triangle: Rituximab (RTX), hexagon: IVIG only, inverse triangle: IVIG + corticosteroids (CS), diamond: IVIG + CS + azathioprine (Az) **(B)** zQ_IgLON5-IgG4_s of the first CSF/serum pair of all five patients compared to the respective z zQ_Total IgG_s. Again, patient 12 is marked by a black border. Statistical comparisons were performed using two-tailed Mann–Whitney *U* test. **(C, D)** The change of the z score for the CSF/serum IgG4 ratio (ΔzQ_IgG4-IgLON5_) in all six intervals with zQ_IgG4-IgLON5_ available at both start and end was plotted against the changes in total IgG IS (zQ_IgG-total_, **C**, left panel), change in serum IgG4 (**C**, middle panel), CSF IgG4 (**C**, right panel), and clinical change **(D)**. Clinical change was defined as −1 = worse (indicated by clinical deterioration and/or increase in mRS), 0 = stable (clinically stable, no change in mRS), 1 = better (clinical improvement and/or reduction of mRS). The results of simple linear regression are depicted. The correlation coefficients obtained by with Pearson correlation and their *p*-values are indicated.

In all five patients, four DRB1*10:01 carriers (Pat 1/9/6/13) and the non-carrier patient 12, definite anti-IgLON5 IgG4 IS (zQ_IgLON5-IgG4_ ≥ 3) could be demonstrated at least at one-time point (**Figure 4A**). Of note, zQ_IgLON5-IgG4_ dropped in patients 9 and 13 after treatment with either RTX+CS (patient 9) or IVIG+CS+Az (patient 13) and again increased in patient 13 after stopping Aza and reducing IVIG frequency, which was associated with clinical relapse. In addition, the median zQ_IgLON5-IgG4_ (20.0, 6.0–25.7) exceeded the zQ_IgG-total_ (1.1, 0.5–2.3, *p* = 0.004) 18-fold with zQ_IgG-total_ being never ≥3 (**Figure 4B**). When changes zQ_IgLON5-IgG4_ during all available intervals were analyzed, zQ_IgLON5-IgG4_ changes only tended to change in the same direction as zQ_IgG-total_ or serum anti-IgGLON5 IgG4 levels (**Figure 4C**, left and middle panels). However, a similar association with changes in CSF anti-IgLON5 IgG4 was highly significant (*p* = 0.008, **Figure 4C**, right panel), confirming CSF anti-IgLON5 IgG as the major influence on zQ_IgLON5-IgG4_. Most importantly, clinical deterioration or improvement during an interval was significantly associated with either an increase or decrease in zQ_IgLON5-IgG4_, respectively(*p* = 0.017, **Figures 4C, D**).

In summary, we could confirm that, in a subset of patients with anti-IgLON5 disease, and likely in those carrying the HLA-DRB1*10:01 allele, anti-IgLON5 IgG4 is synthesized intrathecally and this may be pathophysiologically relevant.

### Combination therapy reduces CSF anti-IgLON5 IgG4 and leads to clinical improvement

**Figure 5:**
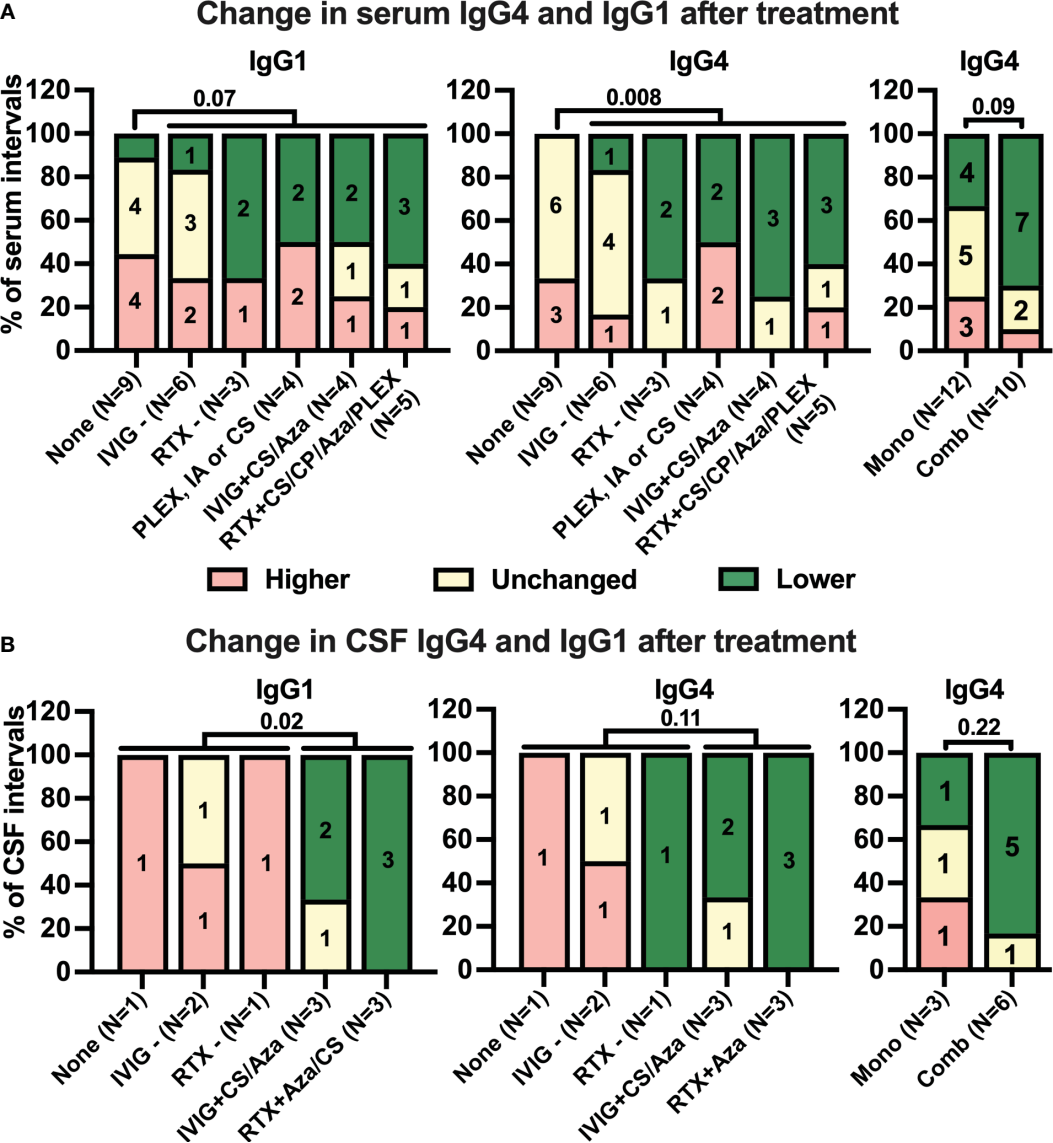
While many treatments tend to reduce serum anti-IgLON5 levels, only combination immunotherapy coincides with reduction of anti-IgLON5 levels in cerebrospinal fluid. Intervals between two consecutive serum **(A)** and cerebrospinal fluid (CSF, **B**) samplings were categorized according to the immunotherapy administrated during the interval as described in the methods sections (None = no treatment, IVIG− = intravenous immunoglobulins only, RTX− = rituximab only, PLEX or CS = plasma exchange or corticosteroids, IVIG +CS/Aza = IVIG + corticosteroids or azathioprine, RTX + CS/CP/Aza/PLEX= rituximab in combination with corticosteroids, cyclophosphamide, azathioprine, or plasmapheresis). The number of intervals is indicated below the bars. Antibody level changes during these intervals were categorized as higher (+> 20%, red), unchanged (± 20%, yellow), or lower (−> 20%, green). Bars show the percentage of intervals assigned to the three antibody levels change categories for anti-IgLON5 IgG1 (left graphs) and IgG4 (middle and right graphs) when grouped according to the treatment regimen. For the right graphs, treatments were dichotomized as monotherapy (Mono) or combination therapy (Comb). In addition, for statistical analysis treatments during the intervals were dichotomized into absence of treatment versus any treatment for serum (**A**, left and middle graph) or no or monotherapy versus combination therapy for CSF (**B**, left and middle graphs). A statistical comparison was performed by one-sided Fisher’s exact test. The *p*-values are indicated above the graphs.

Next, we analyzed how different treatment strategies during the intervals affected anti-IgLON5 IgG1/4 levels. Treatments were categorized either as monotherapies (or combination of short-lived therapies), and combinations of RTX and IVIG with other therapies (for details, see **Figure 1** and Appendix). While there was only a tendency of more frequent decreases in serum anti-IgLON5 IgG1 in intervals with immunotherapy compared to those without therapy [no therapy: one of nine (11%), any therapy: 10 of 22 (46%), *p* = 0.07, **Figure 5A**, left panel, additional information regarding the length of the intervals can be found in **Supplementary Figure S11**], this association was highly significant for anti-IgLON5 IgG4 [no therapy: zero of nine (0%), any therapy: 11 of 22 (50%), *p* = 0.008, **Figure 5A**, middle panel. Interestingly, decreased serum anti-IgLON5 IgG4 was observed in seven of 10 (70%) intervals with combination immunotherapy, but in only four of 12 (33%) with monotherapy. However, due to the low number of data points, this difference failed to reach statistical significance (*p* = 0.09, **Figure 5A**, right panel). In CSF, no treatment (one of one interval, 100%) and immunotherapy with IVIG or RTX alone (three of three intervals, 100%) did not lead to reduced anti-IgLON5 IgG1 levels, but a reduction was found in five of six intervals (83%) with combination therapy (*p* = 0.02 no/mono- compared to combined therapy, **Figure 5B**, left panel). Furthermore, anti-IgLON5 IgG4 levels decreased in one of four intervals (25%) with no or monotherapy (decrease in one interval with RTX only) but in five of six (83%) intervals with combination therapy (six of 10 intervals with any therapy, **Figure 5B** right panels). Due to one interval with response to RTX only, these differences failed to reach statistical significance (no/mono- vs. combination therapy *p* = 0.11, mono- vs. combination therapy, *p* = 0.22).

**Figure 6:**
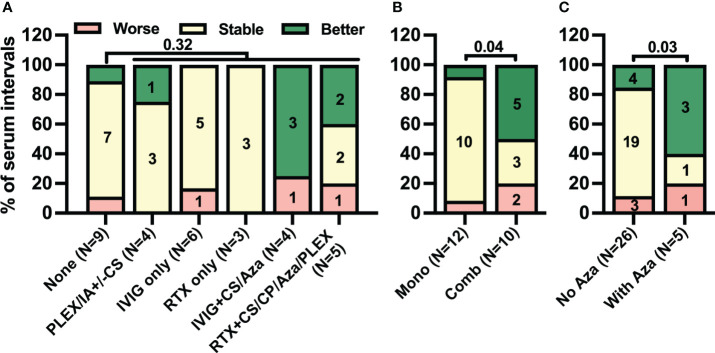
Combination treatment rather than only immunomodulatory treatment is associated with clinical improvement. Intervals between two consecutive serum samplings were categorized according to the immunotherapy administrated during the interval as described in the methods sections (**A**: None = no treatment, IVIG ± CS/Aza = intravenous immunoglobulins with or without corticosteroids or azathioprine, RTX ± CS/Aza/CP/Plex = rituximab with or without CS, Aza, cyclophosphamide or plasma exchange, PLEX or CS = plasma exchange or corticosteroids, **B**: No/Mono = no treatment or monotherapy; **C**: No Aza = without azathioprine, With Aza = with azathioprine). The number of intervals is indicated below the bars. Changes in clinical severity during these intervals were categorized as worse (red), stable (yellow), or better (green) as described in the Materials & Methods section. A statistical comparison was performed by one-sided Fisher’s exact test. The *p*-values are indicated above the graphs.

Next, we investigated clinical responses to different immunotherapy regimens. The frequency of clinical improvement was comparably low during serum intervals with any treatment regimen [six of 22 (27%)] and those without [one of nine (11%), *p* = 0.32, **Figure 6A**]. In contrast, in five of 10 (50%) intervals with combination immunotherapy including either RTX or IVIG but only one of 12 (8%) with monotherapy clinical improvement was observed [**Figure 6B**, *p* = 0.04]. With regard to the combination, in five of 10 intervals with RTX- or IVIG-containing combination immunotherapy (50%) Aza was added. Clinical improvement was observed in three of five (60%) serum intervals with Aza but in only four of 26 (15%) without (*p* = 0.03, **Figure 6C**). Thus, the pattern of clinical improvement in response to therapy closely resembled the decrease in CSF but not serum anti-IgLON5 IgG4 levels.

## Discussion

In this retrospective longitudinal study, we investigated IgLON5 IgG subclass profiles in serum and CSF in 13 IgLON5 patients in the context of the patients’ HLA alleles, clinical presentation, disease severity, and immunotherapy. Our strategy was to analyze all parameters or their change at the beginning and end of each clinical follow-up interval that included sampling of biofluids for assessment of anti-IgLON5 subclass levels, using flow cytometry along with an innovative quantitative technique to quantify the IS of anti-IgLON5 IgG4.

Although the results of our exploratory study often did not meet criteria of statistical significance, they yielded a coherent picture, allowing to generate new hypotheses of the biology of anti-IgLON5 disease, particularly (A) that intrathecally produced CSF IgLON5 IgG4 as well as the cellular inflammatory response may depend on the HLA genotype and, if present, (B) may represent a marker of clinical and prognostic relevance in a subset of patients, and (C) that patients may be more likely to benefit from immunosuppressive treatment in combination. Our main findings that support these hypotheses are that (1) DRB1*10:01 non-carriers show lower CSF-to-serum anti–IgLON5-IgG4 levels and CSF leukocytes when compared to carriers of this risk allele, (2) anti-IgLON5 IgG4 IS was observed not only in all analyzed DRB1*10:01 carriers but also in one non-carrier, (3) the reduction in anti-IgLON5-IgG4 IS correlated with reduced CSF anti-IgLON5 IgG4 and clinical improvement, and (4) low CSF IgG4 levels were associated with future clinical improvement. Further, (5) although many treatments could reduce serum anti-IgLON5 IgG1/4 levels, clinical improvementwas more frequently observed after intense immunotherapy, often containing combinations of either RTX or IVIG with CS, but especially Aza. Taken together these findings led us to form a new hypothesis: that intrathecal anti-IgLON5 IgG synthesis, especially of IgG4, could potentially represent a surrogate marker for disease activity in a subset of patients and may thus be a promising prognostic and/or predictive biomarker. With the limitation that these are preliminary data of a small patient cohort that warrant further studies with larger patient cohorts to validate, we cautiously suggest that both CSF and serum anti-IgLON5 should be monitored. Before becoming available as routine diagnostic tests, the performance of quantitative assays for IgLON5 IgG1 and IgG4 levels along with new strategies to calculate their IS will have to be validated more extensively. Currently, IS of target-specific antibodies is most frequently presented as AI calculated according to Reiber and Lange (31). However, this procedure has two major disadvantages. First, in the AI formula the denominator for the target-specific antibody CSF/serum ratio switches from the actual Q_IgG_ to the 99.5% percentile reference value (Q_lim_) in case the actual Q_IgG_ is higher than Q_lim_. Thus, in cohorts of individuals with and without proven quantitatively relevant intrathecal IgG synthesis according to Reiber (Q_IgG_>Q_lim_) (32), the AIs are calculated by different formulas prohibiting quantitative statistical analysis. Second, when patient in a cohort do not exhibit proven quantitatively relevant IgG synthesis according to Reiber (Q_IgG_≤Q_lim_) but the actual Q_IgG_ is above the 50th percentile of the reference range (as in our cohort), the AI formula tends to underestimate IS as the actual Q_IgG_ (which might indicate a 90% likelihood of IS if, e.g., it is located at the level of the 90th percentile) but not the estimated Q_IgG_ in the absence of IgG IS as the denominator. Both disadvantaged do not occur using the approach suggested by Brauchle et al. and employed in our manuscript, which, in addition, is based on a considerably higher number of data points (27).

Our study shows a predominance of IgG4 in 88% of patients. This is in line with a previous study (25) but in contrast to an earlier study with a lower frequency (33%) (1). The IgG4 predominance in our study was largely due to higher IgG4 levels in IgG4-predominant patients but not lower antibody levels of other subclasses, especially IgG1. Furthermore, in our cohort, which showed the expected high frequency of the known risk alleles HLA-DQB1*05:01 and DRB1*10:01, a higher number of risk alleles seemed to coincide with higher anti-IgLON5 IgG4 levels in serum and CSF. The HLA-DQB1*05 allele has been associated with other IgG4-predominant autoimmune diseases (33–36). Recent data supports the view that HLA-DQ containing a β⁠-subunit encoded by the HLA-DQB1*05:01 anti-IgLON5 disease risk allele plays a pivotal role in the presentation of IgLON5 peptides to T-helper cells and initiation of the anti-IgLON5 immune response (37). The authors suggested that any association of anti-IgLON5 disease with HLA-DRB1*10:01 risk allele may be merely indirect due to its linkage disequilibrium with HLA-DQB1*05:01. Nevertheless, our observations suggest that the presence of HLA-DRB1*10:01 may contribute to disease, as the association of HLA-DRB1*10:01 risk allele with proportionally highly CSF anti-IgLON5 IgG levels most likely representing IS described herein seems to be directly mediated by the HLA-DR genotype. Replicating this observation in larger cohorts might yield relevant insight into the pathoimmunobiology of anti-IgLON5 disease.

Notably, it has been consistently reported that about 10% of anti-IgLON5 disease patients test negative for anti-IgLON5 IgG in CSF [e.g. (38, 39)]. These probably correspond to those with low CSF levels in our cohort when using flow cytometry. In turn, many patients reported to be CSF anti–IgLON5-positive may have target-specific IgG IS. Indeed, one patient each with anti-IgLON5 in CSF only (18) and higher anti-IgLON5 IgG levels in CSF compared to serum have been reported (40). Do these two groups represent different immunological and clinical phenotypes? In the initial description by Sabater et al., one patient with the clinical diagnosis of PSP and anti-IgLON5 IgG in serum only was reported but at this time due to the negative CSF tests it was doubted whether this actually was anti-IgLON5 disease (4). However, later Gaig et al. reported that in anti-IgLON5 disease with PSP-like phenotype CSF may test negative for anti-IgLON5 IgG in 50% of the patients (1). Interestingly, these patients were threefold less likely to carry the DRB1*10:01 risk allele. In our study, only one of four patients with PSP-like anti-IgLON5 disease was DRB1*10:01 carrier. Perhaps the presence of the risk allele predisposes for more robust immigration of antigen-specific B-cells into the intrathecal compartment or brain. Moreover, low intrathecal anti-IgLON5 IgG production may also evade detection in CSF as anti-IgLON5 IgG may be adsorbed in brain tissue due to binding to the membrane-bound and/or shedded antigen (9).

Although clinically distinct from anti-IgLON5 disease, anti-LGI1 AIE equally presents with IgG4 predominance of the target-specific IgG, low titers of target-specific antibodies in CSF compared to serum and largely non-inflammatory CSF results (14, 41, and 42) (41). Of note, not only could clonal expansion of B cells in anti-LGI1 AIE be demonstrated but also that B cells producing LGI1-specific antibodies are present in the CSF (42, 43). Correspondingly, abundant CSF B cells and even plasma cells have been found in most cases with anti-IgLON5 disease (13). These observations might indicate that target-specific recruitment of B cells to the intrathecal compartment with subsequent expansion might be a characteristic of IgG4-predominant autoimmune disorders. In contrast, recruitment of unspecific B cells with production of polyspecific antibodies including multiple anti-viral specificities associated with a robust quantitative IS of total IgG was demonstrated to be a hallmark of AIE with NMDAR antibodies (14, 44), which are largely of the IgG1 and IgG3 subclass. Thus, future experiments should focus on the comparative studies of peripheral and CSF B cells as well as the binding specificity, avidity, affinity and post-translational modifications of target-specific IgG4 produced in both compartments to unravel the relevance of intrathecally produced anti-IgLON5 IgG4.

Which is the best treatment choice for anti-IgLON5 disease? In our small cohort, we observed favorable outcomes mostly after immunosuppressive treatment in combination, which consisted of RTX or IVIG in combination with Aza or CS, or combinations including Aza. However, the number of study participants was low; therefore, further studies are necessary to confirm this observation. Taken together, our findings clearly indicate an urgent need for further studies to investigate the efficacy of combination therapies, particularly involving IVIG or RTX in combination with Aza and CS.

### Limitations

Anti-IgLON5 disease is rare and no guidelines for treatment of anti-IgLON5 disease exist. Thus, our cohort included a limited number of patients with heterogenous treatment regimens. This limits the conclusions that can be drawn from our data. Flow cytometry is a quantitative method, and individual titrations showed linearity of the assay in general, but a high degree of inter-assay variability could not be entirely overcome by normalization. We used four different IgG-subclass–specific secondary antibodies, but these were also used successfully in diseases with IgG1 predominance (45, 46). We decided to measure anti-IgLON5 IgG1/4 at a low dilution to allow detection of low antibody levels, accepting that in exceptional cases with very high antibody levels serum anti-IgLON5 IgG4 levels may exceed the linear range of the assay. We thus optimized the assay to allow a direct comparison of CSF and serum antibody levels and demonstrated that this approach is quantitative, though sensitivity was reduced in patients with very low antibody levels.

## Conclusion

Although anti-IgLON5 disease is rare and the retrospective nature of our study made it difficult to compare results, we present several important findings and hypotheses: (1) intrathecal synthesis of anti-IgLON5 IgG4 may be of clinical and prognostic relevance, as we observed IgLON5-specific IS in all five tested patients (of which four were DRB1*10:01 positive). Further, CSF (but not serum) anti-IgLON5 IgG4 levels correlated with clinical outcomes whereby low CSF levels preceded clinical improvement. (2) Initial observations suggest a potential clinical benefit from combined immunotherapy, as we found that immunotherapy was associated with reduced anti-IgLON5 antibody levels, and combination therapy had greater effect on clinical outcomes. Further studies will be necessary to ascertain our observations in a larger patient cohort.

## Data availability statement

The original contributions presented in the study are included in the article/**Supplementary Material**. Further inquiries can be directed to the corresponding author.

## Ethics statement

The studies involving humans were approved by Ethikkommission Medizinische Universität Wien, Vienna, Austria. The studies were conducted in accordance with the local legislation and institutional requirements. The participants provided their written informed consent to participate in this study.

## Author contributions

IK: Conceptualization, Data curation, Formal analysis, Funding acquisition, Investigation, Methodology, Project administration, Validation, Visualization, Writing – original draft, Writing – review & editing. SM: Resources, Writing – review & editing. MH: Resources, Writing – review & editing. TS-H: Resources, Writing – review & editing. EB-S: Resources, Writing – review & editing. MBl: Resources, Writing – review & editing. MBr: Resources, Writing – review & editing. JD: Resources, Writing – review & editing. LD: Resources, Writing – review & editing. ME: Resources, Writing – review & editing. IF: Resources, Writing – review & editing, Investigation. GF: Investigation, Resources, Writing – review & editing. FF: Methodology, Writing – review & editing. AH: Resources, Writing – review & editing. BH: Resources, Writing – review & editing. VK: Writing – review & editing. SK: Resources, Writing – review & editing. HL: Resources, Writing – review & editing. MN: Resources, Writing – review & editing. JR: Resources, Writing – review & editing. RR: Resources, Writing – review & editing. GR: Writing – review & editing. AS: Writing – review & editing. MS: Writing – review & editing. HT: Writing – review & editing. SW: Resources, Writing – review & editing. TB: Resources, Writing – review & editing. LS: Resources, Writing – review & editing. CG: Resources, Writing – review & editing. JL: Conceptualization, Formal analysis, Resources, Supervision, Validation, Writing – original draft, Writing – review & editing. RH: Data curation, Funding acquisition, Investigation, Resources, Supervision, Validation, Writing – review & editing.

## Footnotes

1
http://www.epidemiolog.net/studymat/.
