# Supplementary material for: HLA dependency and possible clinical relevance of intrathecally synthesized anti-IgLON5 IgG4 in anti-IgLON5 disease
Source: Front Immunol. 2024 May 16;15:1376456. doi: 10.3389/fimmu.2024.1376456 (PMC11141242; doi:10.3389/fimmu.2024.1376456)
Supplement: Supplementary file 1 [file DataSheet_1.pdf]

Supplementary data and Figures

Supplementary data:

<u>Confounders and associations with relative changes of anti-IgLON5 IgG1 and IgG4 levels</u>

Anti-IgLON5 IgG1-4 levels in serum and CSF were measured as ΔMFI in all samples (Supplementary Figure 3, Appendix Figures 1-13). CSF levels of anti-IgLON5 IgG1/4 over time were more variable than serum levels, and IgG1 levels in serum were more variable than IgG4 (Supplementary Figure 4, Supplementary Figure 5). Corresponding to their much higher relative level to IgG1, less change was found for relative serum IgLON5 IgG4 when compared to anti-IgLON5 IgG1 (p=0.0014, Mann Whitney test). Serum anti-IgLON5 IgG1 levels were at follow-up at a median level of 100.7% (42.25-191.0) of their previous level, with 10/43 intervals with a value >120% and 5/43 intervals with a value <80%. Serum IgG4 levels were at their follow up at a median level of 100.0% (51.70-130.7) of their last sample, with 1/46 intervals >120% and 3/46 intervals <80%. The CSF showed a higher variability but no difference between anti-IgLON5 IgG1/4 (IgG1 levels at follow-up: median 93.23%/ (69.73-1784) , 3/10 intervals >120% and 2/10 intervals <80%; CSF IgG4 levels: median 99.78% (18.47-211.3), 1/10 intervals >120% and 4/10 intervals <80%). In contrast to CSF, a significant higher absolute serum anti-IgLON5 IgG1 deviation from 100% compared to serum anti-IgLON5 IgG4 was observed, calculated as absolute value of 100% minus percent value at follow up (p=0.0014, Mann Whitney test, supplementary figure 3B).

Anti-IgLON5 IgG4 predominated in 38/46 (83%) serum, but only 11/20 (55%) CSF samples (p=0.022), IgG1 in 1/46 (2%) serum and 1/20 (5%) CSF samples. IgG2+3 ΔMFI, when added up, comprised less than 10% of IgG1+4 ΔMFI in 45/46 (98%) sera and 15/20 (75%) CSF samples. IgG4 predominance of anti-IgLON5 IgG in both serum and CSF was associated with significantly higher anti-IgLON5 IgG4 levels, but not lower anti-IgLON5 IgG1 levels compared to non-predominance (IgG4: serum p=0.0026, CSF p=0.0023; IgG1: serum p=0.701,

CSF p=0.4561; Supplementary Figure 6). As expected, lower serum IgG4/IgG1 ratios were associated with higher IgG1 levels (Supplementary Figure 6A, left panel) but also a trend for higher IgG4 levels in serum, which did not reach statistical significance (Supplementary Figure 6B, left panel).

Supplementary figures:

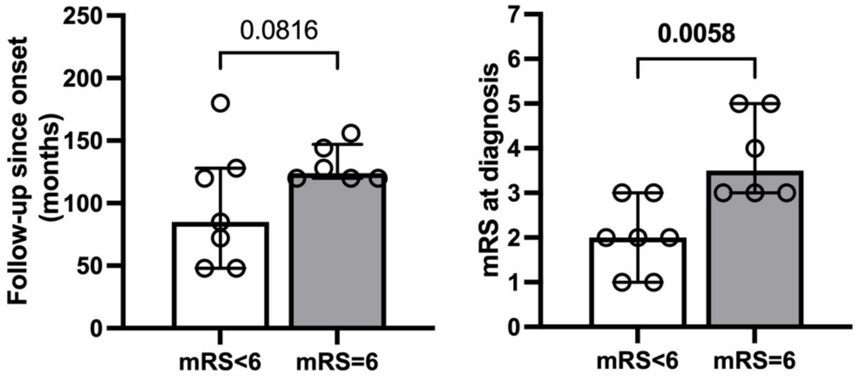

Supplementary Figure S1: Patients that died and were more severely affected at time of diagnosis. Left figure: follow-up time since onset of symptoms in months, right figure: mRS at time of diagnosis. P-values are indicated in the graph. Mann Whitney test. Note that the follow-up time only showed a tendency for a longer duration.

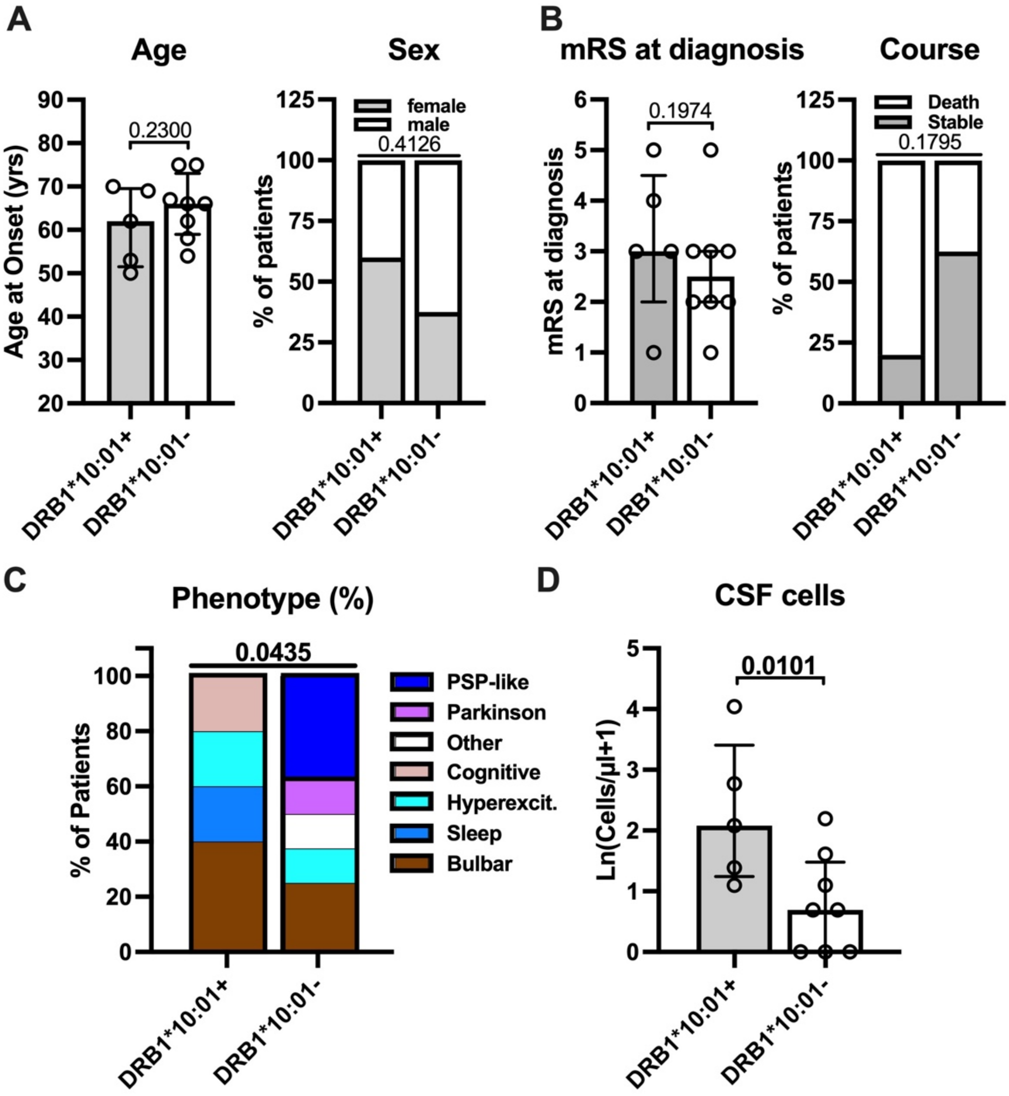

Supplementary Figure S2: Clinical parameters of carriers and non-carriers of the HLA-DRB1*10:01 risk allele. (A) Age of onset and sex distribution, (**B**) mRS at diagnosis and course of the disease. Patients who were progressive all died. Stable: unchanged or improved. (C) Predominant disease phenotypes. (D) CSF cells as first lumbar puncture. A/B left panels and D: one tailed Mann Whitney U test; A/B right panels and C: one-tailed Fisher's exact test. For C number of PSP-like phenotype and other phenotype were compared to genotype.

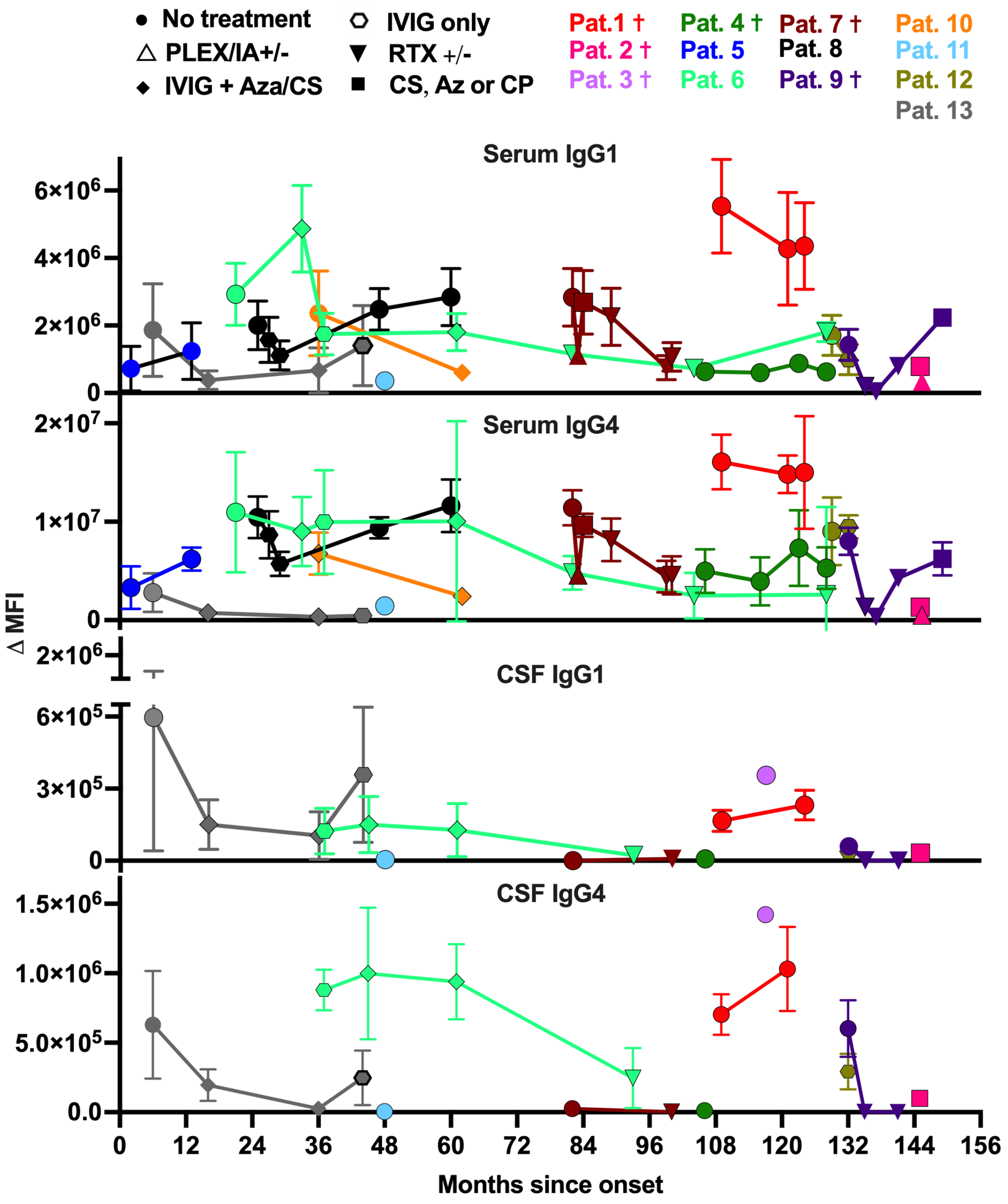

Supplementary Figure S3: Longitudinal changes in anti-IgLON5 IgG1 and IgG4 levels in serum and CSF in a cohort of 13 anti-IgLON5 disease patients. Individual IgG1 and IgG4 levels measured by flow cytometry were colour-coded as indicated and plotted as mean ± SEM fluorescence intensity above background (ΔMFI) against the timepoint of sampling as months since onset. The treatment preceding each sampling is coded by the symbols that represent mean antibody level. Patient 2 had multiple short-term serum samplings during plasma exchange, of which only the first and last are depicted. PLEX/IA = plasma exchange or immune adsorption (triangle), IVIG -/+ = intravenous immunoglobulins with or without additional treatment (rhomboids), IVIG only (hexagons), RTX-/+ = rituximab with or without additional treatment (inverted triangles), CS, Az or CP = corticosteroids, azathioprine or cyclophosphamide (squares). Crosses indicate that the patient is deceased.

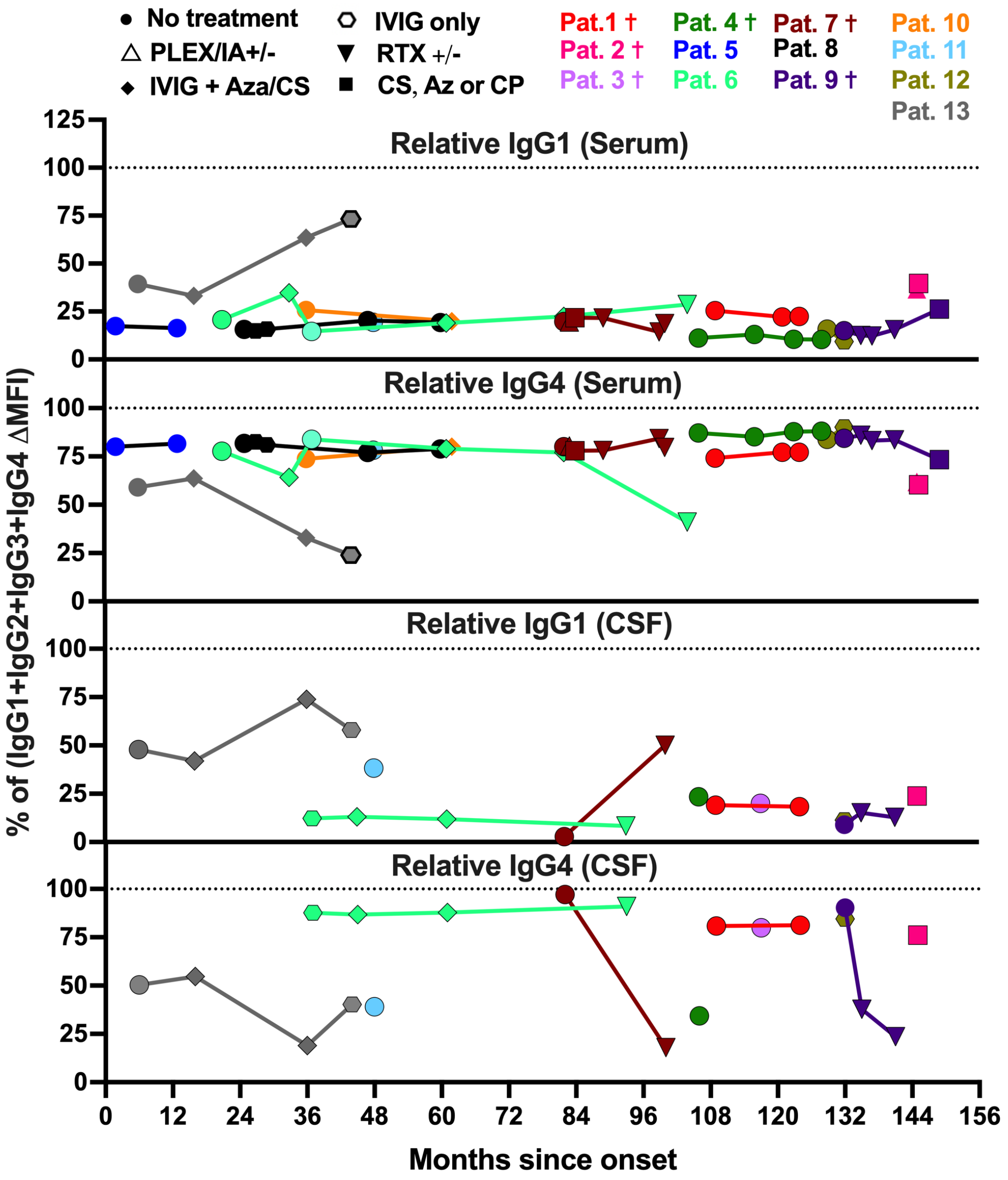

Supplementary Figure S4: Change of IgLON5 IgG1 and IgG4 relative proportion in longitudinal serum and CSF samples. IgG1 and IgG4 titres were measured by flow cytometry (ΔMFI) as in Supplementary Figure 3. The mean ΔMFIs for IgG1 and IgG4 in CSF and serum were normalized by the sum of all mean ΔMFI for anti-IgLON5 IgG1, IgG2, IgG3 and IgG4 as surrogate marker for total anti-IgLON5 IgG (see Appendix Figure 1-13 for depiction of the raw data) and are expressed as percentage of "total" anti-IgLON5 IgG. X axis: time scale indicating the time after disease onset in months. PEX= plasmapheresis, CS= corticosteroids, RTX= rituximab, AZ= Azathioprine, IVIG= intravenous immunoglobuline, CP= cyclophosphamide. Cross indicates that the patient is deceased. Colours indicate individual patients.

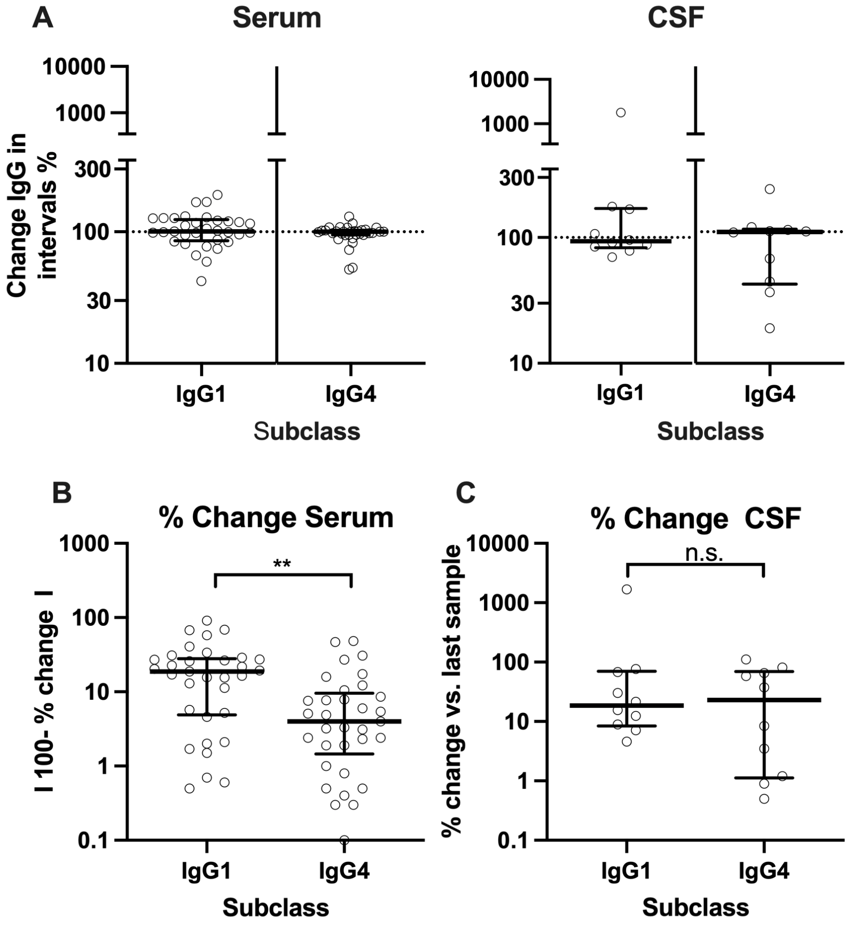

Supplementary Figure S5: Analysis of percent change of IgLON5 IgG1 and IgG4 levels during intervals in the serum and CSF demonstrates higher variability in serum IgG1 then IgG4, and higher variability in CSF antibody levels than in serum antibody levels. No difference in change was found between CSF IgG1 and IgG4, supporting the finding that IgG1 variability is found specifically in the serum. (A) Change in antibody levels depicted as percentage of last follow-up. (B) Change in antibody levels at next follow-up depicted as deviation from 100%. Mann Whitney test. **: p=0.0014.

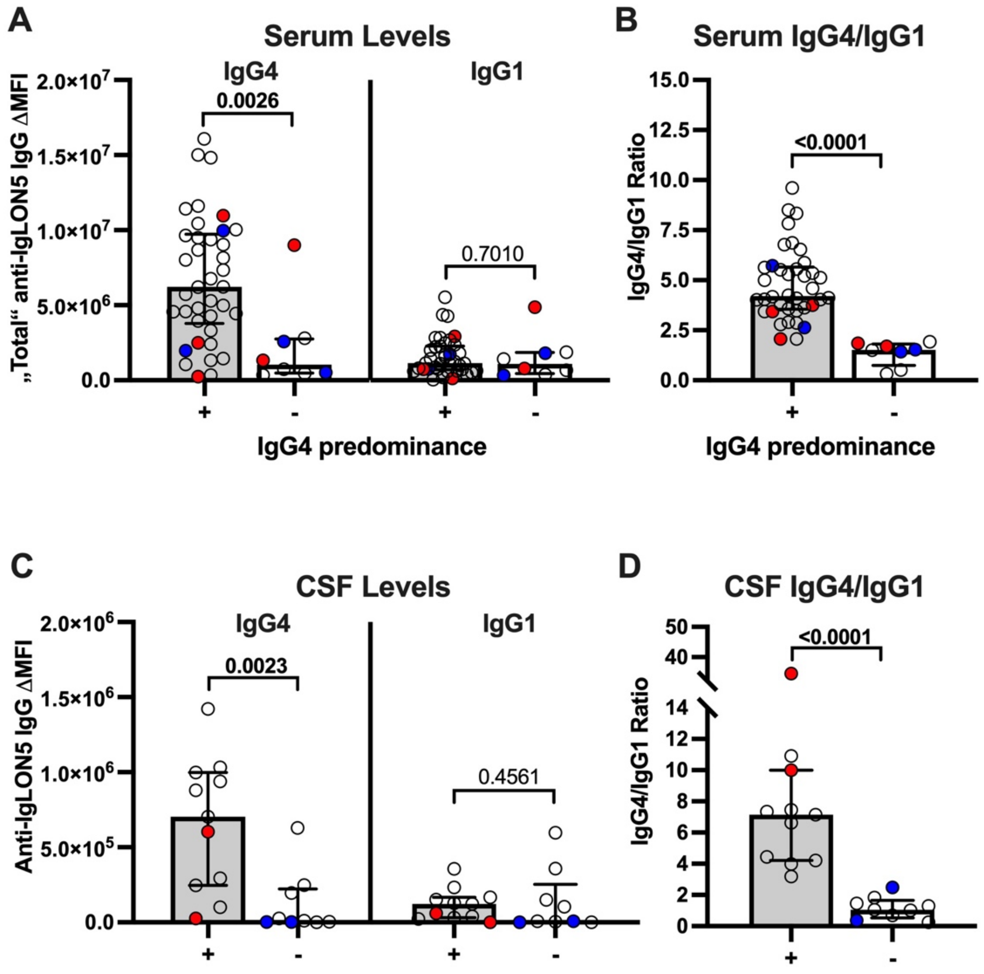

Supplementary Figure S6: Anti-IgLON4 IgG4 predominance is determined by higher IgG4 levels rather than lower IgG1 levels in both serum and CSF. (A) Serum anti-IgLON5 IgG1 and IgG4 levels. (B) Serum anti-IgLON5 IgG4/IgG1 ratio. (C) CSF anti-IgLON5 IgG4 and IgG1 levels, (D) CSF anti-IgLON5 IgG4/IgG1 ratio. Colours indicate a change of predominance in comparison to the last (blue) or next (red) follow-up. Note that for serum but not for CSF most IgG4/IgG1 datapoints were close to the cut-off of 2, which is explained by higher variation IgG2 and IgG3 levels in the CSF compared to serum. Mann-Whitney test.

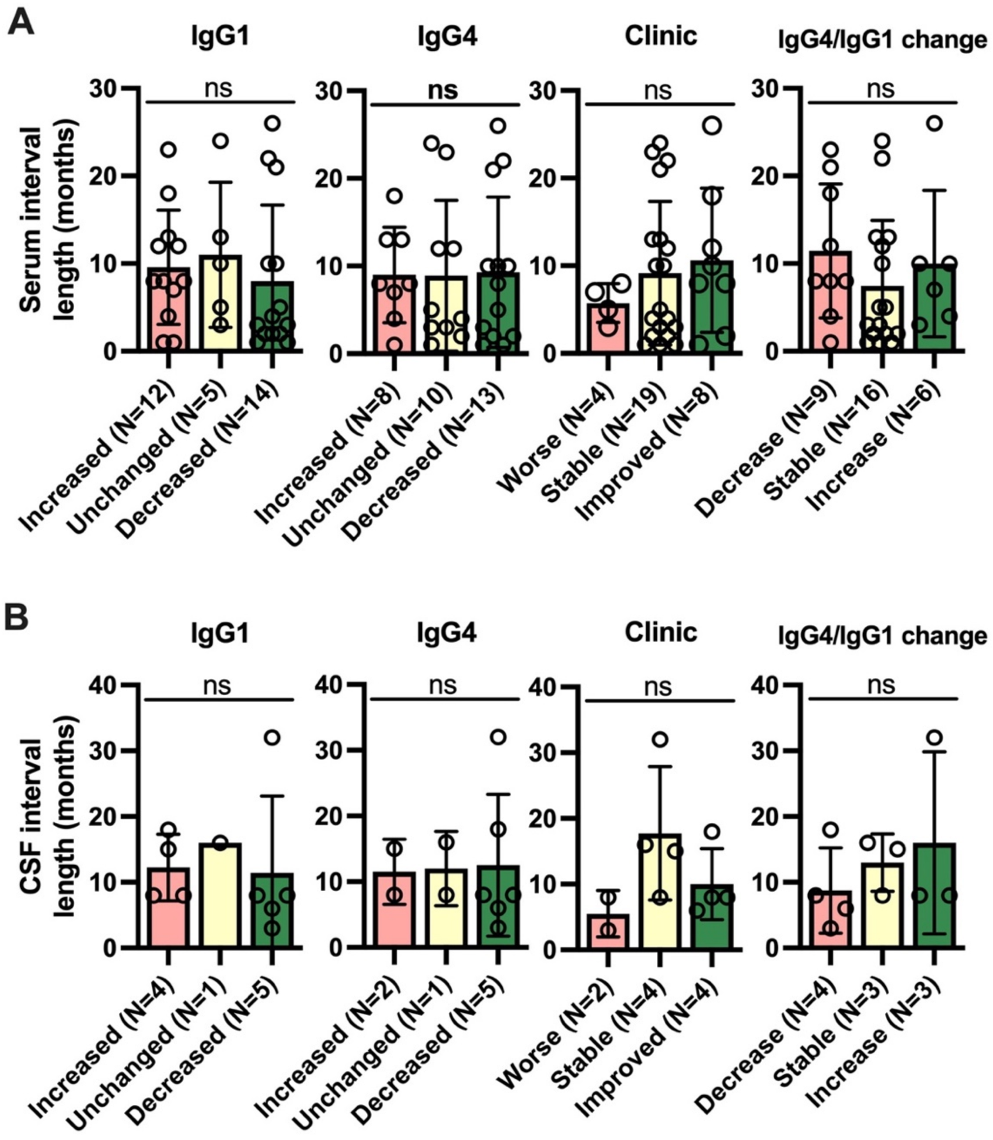

Supplementary Figure S7: Interval duration was similar across intervals with distinct changes in anti-IgLON5 IgG1 and IgG4 levels or clinical changes. (A) Serum intervals, (B) CSF intervals. No significant differences, Kruskal-Wallis test.

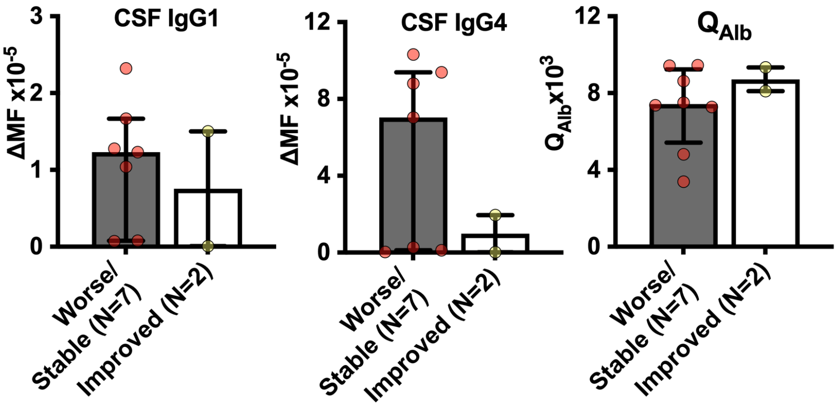

Supplementary Figure S8: **Decreased CSF anti-IgLON5 IgG4 levels prior to clinical improvement are not associated with a corresponding decrease in the CSF/serum albumin ratio.** The CSF IgG1 (left graph) and IgG4 datapoints (middle graph) of patients with CSF/serum ratio (Q_{Alb}) available as well as the Q_{Alb}s (right graph) were grouped according to future clinical changes as in the left panels of Figure 3B/D but with worse and stable future outcome combined.

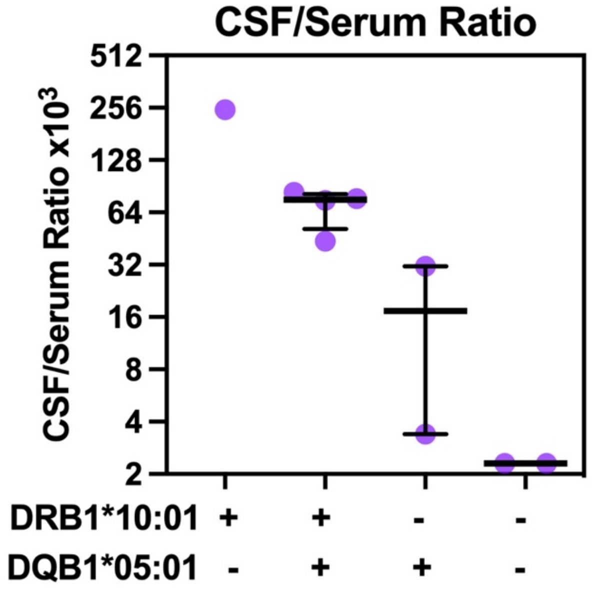

Supplementary Figure S9: Carriership of HLA-DRB1*10:01, rather than DQB1*05, is associated with elevated CSF/serum ratio of IgLON5 IgG4. The CSF/Serum ratio of anti-IgLON5 IgG4 in patients carrying one or two alleles of DRB1*10:01, DQB1*05:01 or no alleles.

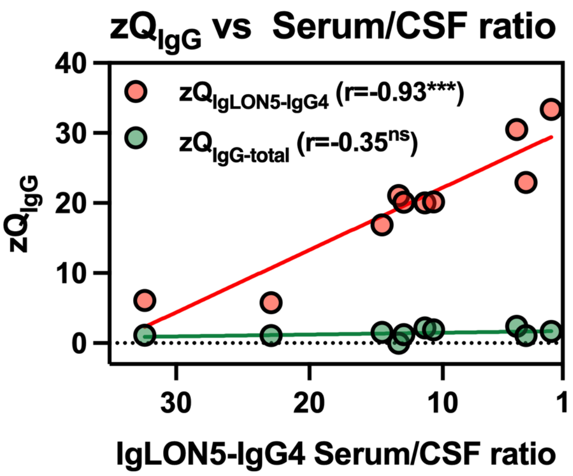

Supplementary Figure S10: Precise measurement and calculation of the intrathecal anti-IgLON5 IgG4 synthesis correlated with the IgLON5-IgG4 serum/CSF ratio. All z scores for the IgLON5-IgG4 specific CSF/serum ratio ($zQ_{IgG\text{-}IgLON5}$, red) that could be obtained were plotted against the corresponding ratios in serum and CSF as shown in Supplementary Figure 3. The corresponding z scores for the total CSF/serum ratio were plotted for comparison ($zQ_{IgG\text{-}total}$, green). One datapoint (second $zQ_{IgG\text{-}IgLON5}$ from patient 9 in Figure 4A) excluded as outlier, as CSF values were negligible after rituximab treatment and below a reliable detection limit, leading to a serum/CSF ratio of 2800, which was considered unlikely. The results of simple linear regressions are depicted. The results of Pearson correlation are indicated, n.s. not significant, ****p<0.0001.

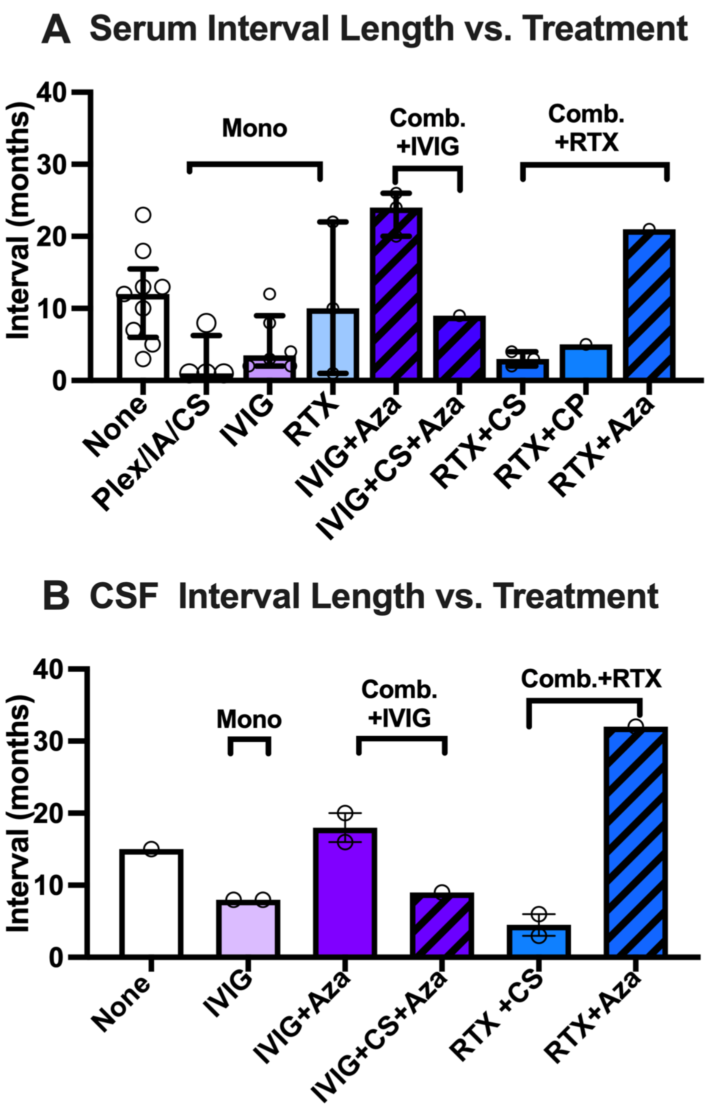

Supplementary Figure S11: Duration of sampling intervals with different treatments. (A) Serum and (B) CSF interval length after different treatments. Mono =Monotherapy, Comb.= combination therapy. IVIG = intravenous immunoglobulins, Aza = azathioprine, CS = corticosteroid, RTX = rituximab. Treatment regiments containing IVIG are colored violet, those containing RTX blue. Note the comparably longer intervals when treatment regiments containing Aza were administered, which might indicate longer clinical stability not requiring repeat sampling.

Supplementary Table S1: Symptoms and predominant clinical phenotype.

Patient	Neurologic profile	Sleep disorder	Bulbar syndrome	Bradykinesia/Dystonia	Gait instability	Oculomotor abnormalities	Cognitive impairment	Autonomic dysfunctiion	Peripheral hyperexcitability (motor)	Peripheral hyperexcitability (sensory)	Tremor	1. MND	2. MND
2	Sleep disorder												
9	Bulbar syndrome												
5	Bulbar syndrome												
7	Bulbar syndrome												
1	Bulbar syndrome												
3	PSP-like												
12	PSP-like												
4	PSP-like												
10	Parkinsonism												
11	Unclassified												
6	Cognitive												
8	Peripheral hyperexcitability												
13	Peripheral hyperexcitability												

Parasomnia confirmed by video-EEG
History of parasomnia not video-EEG confirmed
Other sleep dysturbances in the absence of parasomnia

mild
moderate to severe

mild
moderate to severe

Severe, repeated falls
mild
subjective

supranuclear gaze palsy
other oculomotor problems

Dementia
mild cognitive impairment

bladder dysfunction, bradycardia, hyperhidrosis

fasciculations, cramps, stiffness, itching, pain

paresis, atrophy, denervation

predominant spasticity, hyperreflexia
mild

Supplementary Table S2: Synopsis of clinical phenotype, demographic characteristics and laboratory findings.

Patient	Neurologic profile	Age at Onset	Sex	DRB1*10:01	% IgG4 serum	CSF cells	OCB	Serum-to-CSF-IgG4 ratio
1	Bulbar syndrome	69	female	+	74%	<4	-	23
2	Sleep disorder	53	female	+	62%	2	n.d.	13
3	PSP-like	66	male	-	80%	1	-	n.d.
4	PSP-like	62	male	-	87%	0		433
5	Bulbar syndrom	75	female	-	80%	n.d.	n.d.	n.d.
6	Cognitive	62	female	+	78%	7	-	12
7	Bulbar syndrome	67	female	-	80%	2	-	429
8	Peripheral hyperexitability	58	male	-	82%	8	-	n.d.
9	Bulbar syndrome	50	male	+	84%	15	-	13
10	Unclassified	54	female	-	74%	n.d.	n.d.	n.d.
11	Parkinsonism	75	male	-	78%	<5	n.d.	292
**12**	PSP-like	66	male	-	84%	1	-	32
13	Peripheral hyperexitability	70	male	+	59%	56	+	4

Supplementary Table S3: HLA haplotype data of 13 IgLON5 patients.

Patient	HLA result
Patient 1	**DRB1*10:01**, **DQB1*05:01** /DRB1*09:01, DQB1*03:03
Patient 2	**DRB1*10:01, DQB1*05:01**
Patient 3	DRB1*01:02P, **DQB1*05:01**/ DRB1*03:01P, DQB1*02:01
Patient 4	DRB1*03:01, DQB1*02:01/ DRB1*14:01, *DQB1*05:03*
Patient 5	DRB1*03:01, DQB1*02:01/DRB1*04:01, DQB1*03:02
Patient 6	**DRB1*10:01, DQB1*05:01**/DRB1*07:01, DQB1*02:02
Patient 7	DRB1*14:01P, *DQB1*05:03P/*DRB1*03:01P, DQB1*02:01
Patient 8	DRB1*01:01, **DQB1*05:01**
Patient 9	**DRB1*10:01, DQB1*05:01**
Patient 10	DRB1*01:01, **DQB1*05:01**/DRB1*15:01, DQB1*06:02
Patient 11	DRB1*01:01, **DQB1*05:01**/DRB1*04:0, DQB1*03:01
Patient 12	DRB1*01:01, **DQB1*05:01**
Patient 13	**DRB1*10:01**, DQB1*06:02 / DRB1*13, DQB1*06:03

Supplementary Table S4: HLA class II associations in a cohort of 11 Austrian patients (patients 1-8 and 10-12) with anti-IgLON5 disease. Significant associations are shown in **bold.**

	Controls		IgLON5 patients					Fisher
HLA Allele	Positive	Negative	Positive	Negative	OR	Lower 95% CI	Upper 95% CI	2-tailed p-value
DQB1*02:01	48	152	4	7	1.80	0.37	7.46	0.55
DQB1*02:02	35	165	1	10	0.47	0.01	3.52	0.82
DQB1*03:02	27	173	1	10	0.64	0.01	4.85	1.11
DQB1*03:03	12	188	1	10	1.57	0.03	12.81	1.02
**DQB1*05:01***	**48**	**152**	**8**	**3**	**8.44**	**1.90**	**50.71**	**0.002**
DQB1*05:03	10	190	2	9	4.22	0.39	24.54	0.245
DRB1*01:01	41	159	4	7	2.22	0.45	9.18	0.37
DRB1*01:02	7	193	1	10	2.76	0.06	25.10	0.71
DRB1*03:01	44	156	4	7	2.03	0.41	8.37	0.44
DRB1*04:01	27	173	2	9	1.42	0.14	7.41	0.92
DRB1*07:01	49	151	1	10	0.31	0.01	2.27	0.44
DRB1* 09:01	3	197	1	10	6.57	0.11	89.02	0.39
**DRB1*10:01***	**1**	**199**	**3**	**8**	**74.63**	**4.94**	**3933**	**0.00083**
DRB1*14:01	9	191	2	9	4.71	0.43	27.99	0.21
DRB1*15:01	12	188	1	10	1.57	0.03	12.81	1.02
