# Supplementary material for: HLA dependency and possible clinical relevance of intrathecally synthesized anti-IgLON5 IgG4 in anti-IgLON5 disease
Source: Front Immunol. 2024 May 16;15:1376456. doi: 10.3389/fimmu.2024.1376456 (PMC11141242; doi:10.3389/fimmu.2024.1376456)
Supplement: Supplementary file 2 [file DataSheet_2.pdf]

Supplementary Methods

1.1 Flow cytometry gating strategy

Flow cytometrical analysis of human IgLON5-IgG positive serum bound to HEK293 cells transfected with IgLON5-tGFP (derived from Origene, RG22549) using biotinylated anti-human IgG1, IgG2, IgG3 or IgG4, respectively, and visualized with streptavidin-Cy3. After staining, intact cells were selected (FSC-A: forward scatter area/ SSC-A: side scatter area) in gate P1 (Supplementary Methods figure 1A), followed by selection of single cells (FSC-width: forward scatter width – FSC height: forward scatter height) in gate P2 (Supplementary Methods figure 1B). Transfected and untransfected cells were identified based on their green fluorescence intensity (B525-FITC-A) due to enhanced green fluorescent protein (tGFP) expression in transfected cells and either gated as untransfected/tGFP-negative (gate P8) and transfected/tGFP-positive (gate P6, Supplementary Methods figure 1C).

The binding of patient antibodies can be visualized as red fluorescence derived from Cy3, which is measured in the Y585-PE channel, as shown in gate 2, where clearly two distinct cell populations can be seen: the transfected cells (which are tGFP positive, also seen as P6 in (C))

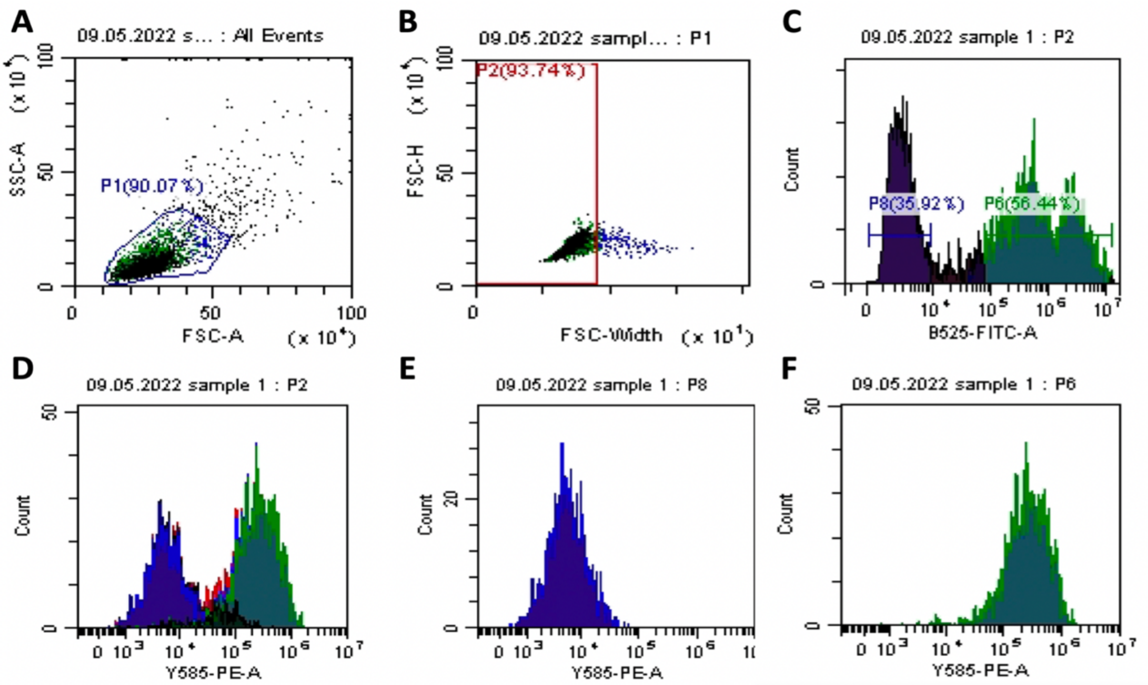

Supplementary Methods Figure 1: Example flow cytometry data of anti-IgLON5-IgG4 positive serum. (A) Gating on intact cells (FSC-A: forward scatter area/ SSC-A: side scatter area) in gate P1, followed by (B) selection of single cells (FSC-width: forward scatter width – FSC height: forward scatter height) in gate P2. (C) Transfected and untransfected cells were identified based on their green fluorescence intensity (B525-FITC-A) due to Turbo green fluorescent protein (tGFP) expression in transfected cells and either gated as untransfected/tGFP-negative (gate P8) and transfected/tGFP-positive (gate P6). (D) The median red (Cy3, Y585-PE-A) fluorescence intensity (MFI) of cells from gate 2, including transfected cells in gate 6 (indicated in green) and untransfected cells in gate 8 (indicated in blue) showed the binding of patient antibodies. (E) Antibody binding of untransfected cells selected by gate P8 and (F) of transfected cells selected by gate P6 was measured as median red (Cy3) fluorescence intensity (Y585-PE-A).

show a stronger red signal (indicated in green) than the untransfected cells (which are tGFP negative, indicated in blue, also seen as P8 in (C)). To quantify antibody binding, the red (Cy3) signal of untransfected cells selected by gate P8 (Supplementary Methods figure 1E) and of transfected cells selected by gate P6 was measured (Y585-PE-A, Supplementary Methods figure 1F). The median red fluorescence intensity (MFI) from untransfected cells ($MFI_{untransfected}$) incubated with human IgLON5-IgG-positive serum and subsequently biotinylated anti-human IgG1, IgG2, IgG3 or IgG4, respectively, followed by streptavidin-Cy3 (P8) was subtracted from median red fluorescence intensity of transfected cells ($MFI_{transfected}$) treated similarly (P6) to account for unspecific binding of the respective IgG subclass to HEK293 cells. The difference of $MFI_{transfected}$ and $MFI_{untransfected}$ (ΔMFI) was used as a marker for bound IgLON5-specific IgG of the respective subclass.

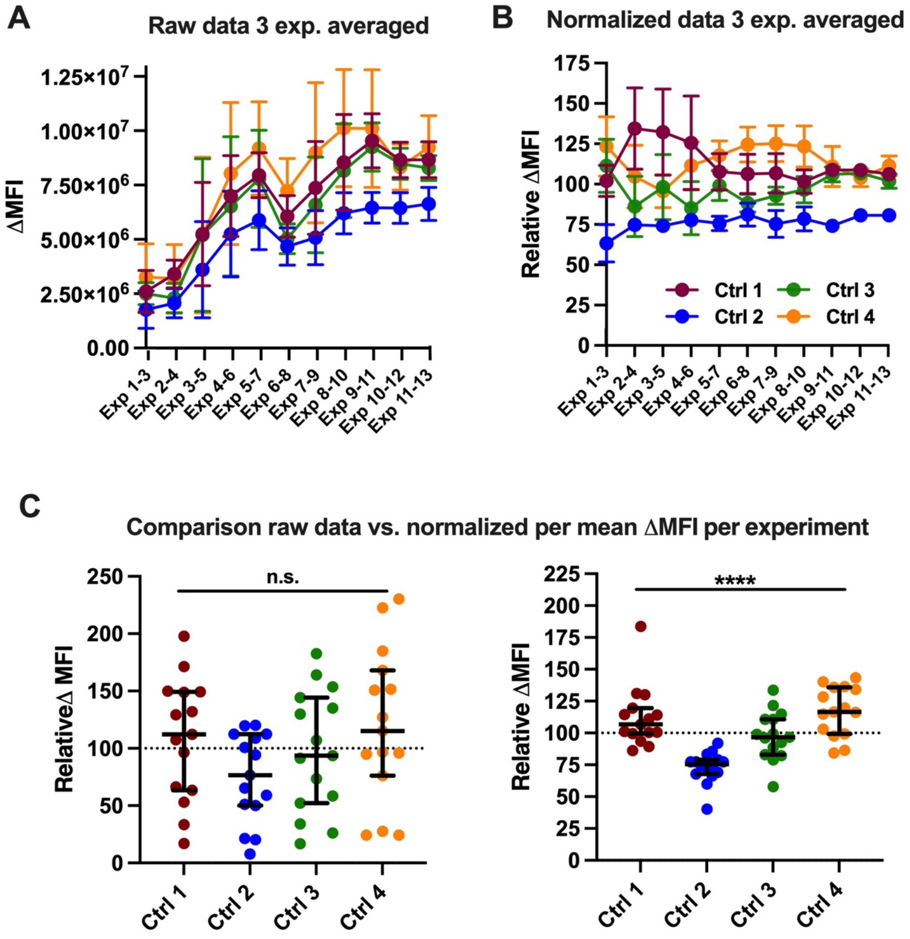

Supplementary Methods Figure 2: **Inter-assay variability and normalization efficiency. The first four sera from patient 8 (Ctrl 1-4) were used as standards in each experiment.** (A) IgLON5 IgG4 ΔMFI raw values of the four standards averaged from 3 experiments each, (B) Distribution of values after normalization. (C) Summarized raw data of the four control samples, (D) summarized data of the four control samples after normalization. Brown-Forsythe and Welch ANOVA tests and Kruskal-Wallis test, $p<0.0001$.

1.2 Normalization of ΔMFI data

The ΔMFI results were multiplied by the dilution factor to account for different serum and CSF volumes used in the experiments. For the purpose of normalization in order to reduce the inter-assay variability, four standard samples (the first four sera from patient 8) were measured in each experiment. To adjust for different general levels of fluorescence intensities across all samples among the different experiments, the IgLON5 IgG ΔMFI value of each sample was divided by the mean IgLON5 IgG4 ΔMFI of the four standard samples of the individual experiment. The resulting values were for each sample were then multiplied with the average IgLON5 IgG4 ΔMFI value of the four standard samples across all experiments.

Supplementary Methods Figure 2 shows the inter-assay variability and the efficiency of the normalization procedure by showing the results of the four standard samples (Supplementary Methods Figure 2).

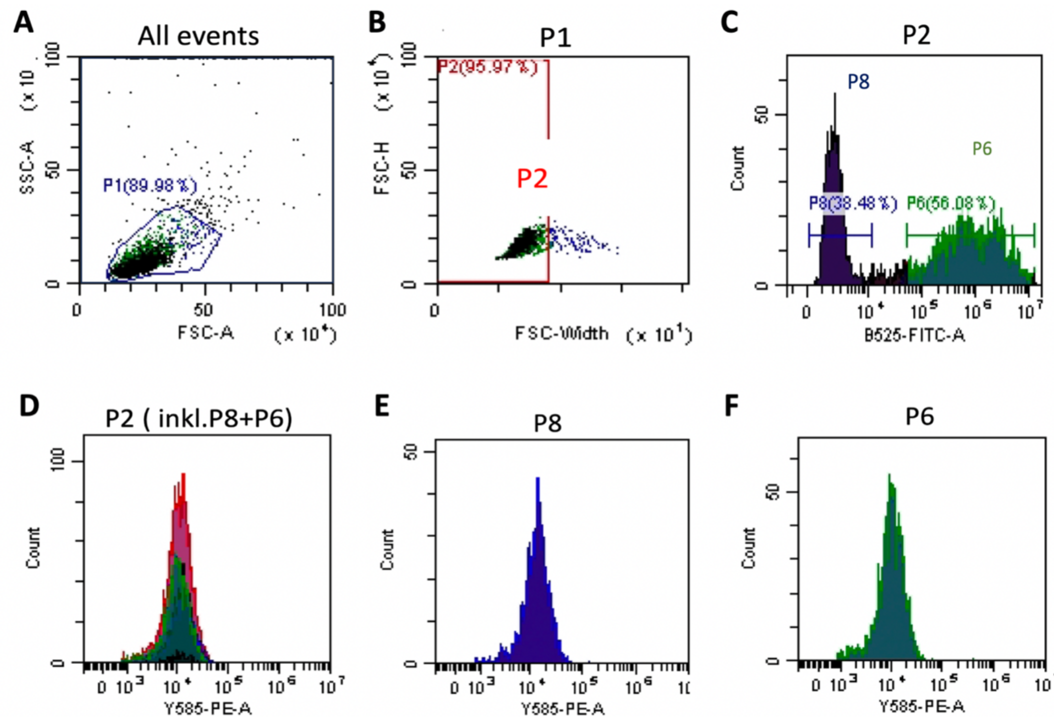

Supplementary Methods Figure 3: Example flow cytometry data of anti-IgLON5-negative serum. (A) Gating on intact cells (FSC-A: forward scatter area/ SSC-A: side scatter area) in gate P1, followed by (B) selection of single cells (FSC-width: forward scatter width – FSC height: forward scatter height) in gate P2. (C) Transfected and untransfected cells were identified based on their green fluorescence intensity (B525-FITC-A) due to Turbo green fluorescent protein (tGFP) expression in transfected cells and either gated as untransfected/tGFP-negative (gate P8) and transfected/tGFP-positive (gate P6). (D) The median red (Cy3, Y585-PE-A) fluorescence intensity (MFI) of cells from gate 2, including transfected cells in gate 6 (indicated in green) and untransfected cells in gate 8 (indicated in blue) showed the binding of patient antibodies. (E) Antibody binding of untransfected cells selected by gate P8 and (F) of transfected cells selected by gate P6 was measured as median red (Cy3) fluorescence intensity (Y585-PE-A).

1.3 Assay Specificity

To determine specificity of the assay, anti-IgLON5 negative sera from three different healthy controls were subjected to the test for anti-IgLON5 IgG1, IgG2, IgG3 and IgG4, respectively, in three repeat experiments. In all experiments, binding of the respective IgG subclass from anti-IgLON5 negative sera to IgLON5 expressing cells (P6) was lower than to untransfected cells (P8) resulting in slightly negative values for ΔMFI (Supplementary Methods Figure 3,4). This may derive from reduced unspecific binding of IgG to IgLON5-expressing cells, although the mechanism is unclear. Alternatively, the reduction of the red signal in the PE channel derived from overcompensation of the FITC channel.

While therefore the values for IgG1, 2, 3 and 4 in healthy control sera were below zero in all experiments at all tested dilutions, this was not the case for serum and CSF samples from patients with anti- IgLON5 disease (Supplementary Methods Figure 4 A/B/C, Supplementary Figures 1-13). For the further analysis of the IgG subclass profiles, particularly for the graphical presentation of the profiles (Supplementary Figure 1-13), the ΔMFI values of any samples that were < 0 (which affected mostly healthy control sera and IgG2, IgG3) were rounded up to a value of 0.

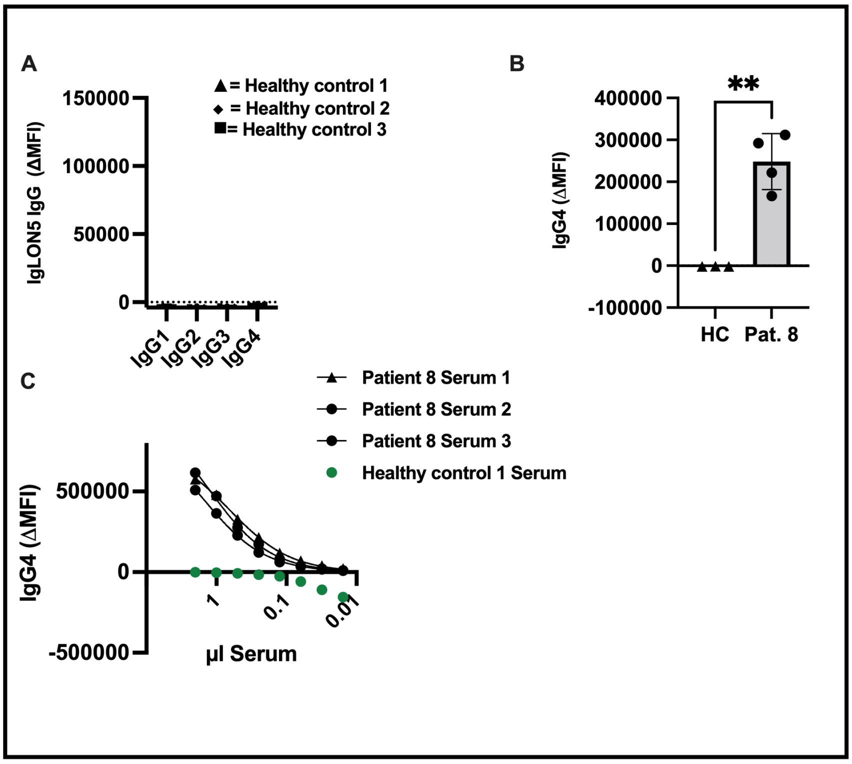

Supplementary Methods Figure 4: No detection of anti-IgLON5-like immunoreactivity against IgLON5-transfected HEK293 cells when using anti-IgLON5 negative control sera. Healthy controls showed ΔMFI values below zero for all IgG subclasses at 1:40 dilution (n=3)(A), in contrast to control sera from patient 8 at a dilution of 1:40 (n=3) (B) and at different dilutions (n=1) (C). A-B shows mean + SD.

1.4 Determination of intra-assay variability and linearity of measurements.

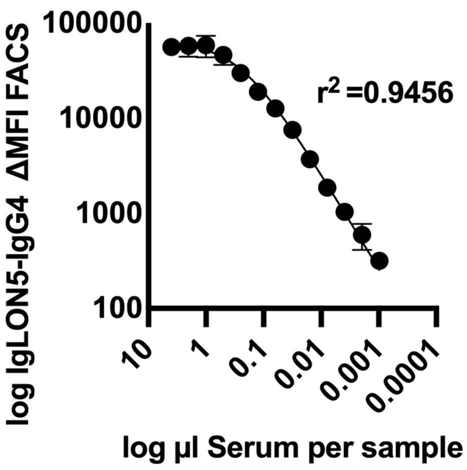

Supplementary Methods Figure 5: Mean fluorescence intensities derived by titration of high-titre IgLON5-positive serum of patients. 1. Serum samples were measured in one experiment with duplicates. “One-site specific binding” curve fitting based on the equation Y = Bmax*X/(Kd + X) was applied in Graphpad prism.

For Figures 1-4, serum and CSF IgLON5 IgG levels were assessed using the flow cytometrical analysis using a dilution of 1:40 (1 µl) and 1:10 (4 µl), respectively. As for the analysis of intrathecal anti-IgLON5 IgG4 synthesis strict linearity of the measurements was required, the linearity of the assay was re-examined. To this end, increasing volumes (starting with 0.001 µl) of the serum with the highest anti-IgLON5 antibody level (Figure 1) were analysed using the flow cytometry assay (Supplementary Methods Figure 5). In addition, intraassay variability of the duplicates for each volume was assessed.

The results indicate that the data points in this assay follow the “One-site specific binding” curve fitting based on the equation Y = Bmax*X/(Kd + X) in GraphPad Prism 9, with Bmax defined as maximum specific binding in the same units as Y (in this case 58190 to 72021 ΔMFI), and Kd defined as the equilibrium dissociation constant, in the same units as X (in this case 0.17 to 0.36 µl serum per sample). Intra-assay variability was observed by variability between duplicate measurements. Saturation was reached at serum volumes of >0.1 µl corresponding to a dilution of 1:400 or less.

Next, additional serum and CSF samples with either high or medium/low IgLON5 IgG levels in increasing volumes were used similarly to determine whether volumes of serum or CSF obtained by dilutions of 1:40 and 1:10, respectively, results in ΔMFI values (red dots) within the linear rage of the assay or within the plateau phase of the curve (Supplementary Methods Figure 6).

We concluded that although majority of sera and CSF samples at 1:40 and 1:10 dilution, respectively, were in the linear range of binding for IgLON5 IgG4, serum and serum samples with very high IgLON5-IgG4 antibody concentration (Patient 1, see also figure 1 in the main manuscript) were above the linear range at dilutions used for the assessments shown in Figures 1-4. However, since the majority of samples had lower autoantibody concentrations, and IgLON5- IgG1, 2 and 3 subclass antibody concentrations were significantly lower than IgG4 concentrations, most but not all IgLON5 antibodies levels shown or analyzed in Figures 1 - 4

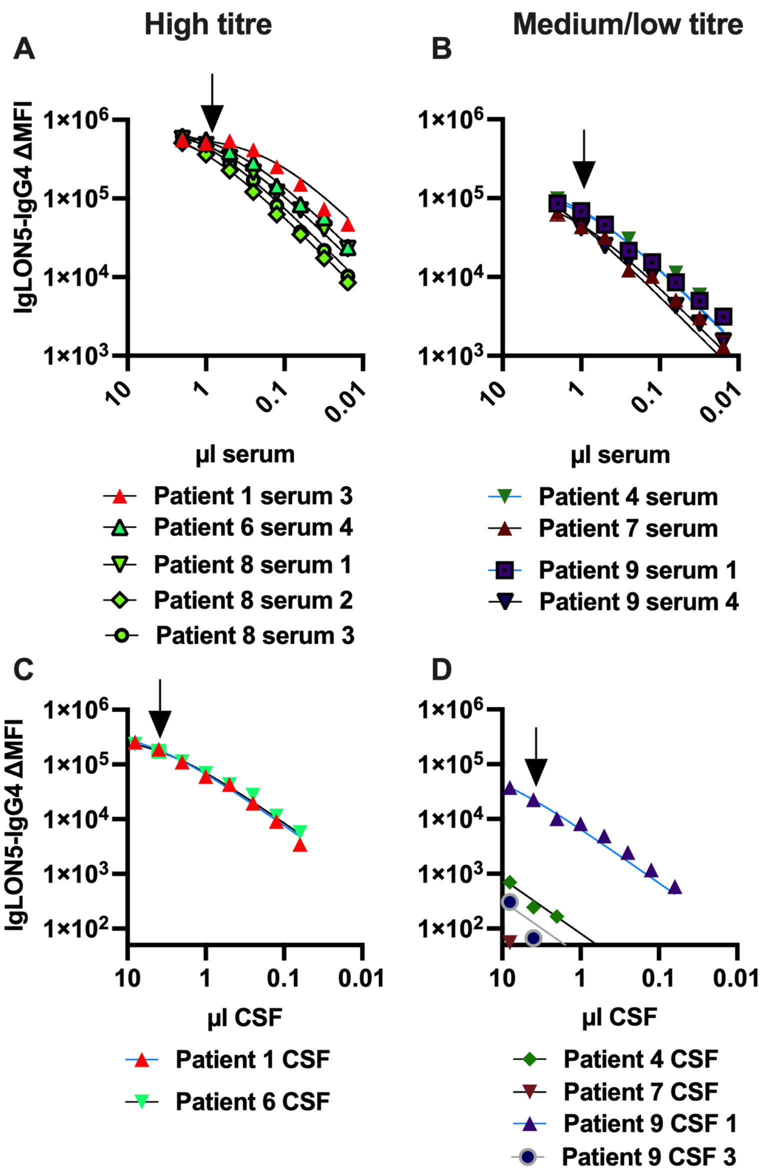

Supplementary Methods Figure 6: **Linearity of the assay when using of decreasing volumes of sera and CSF with either high or medium/low anti-IgLON5 IgG4 levels.** A CSF samples from patient 1 (blue) and 6 (green), B) serum samples from patient 1(blue), 6 (green) and 8 (black). Arrows indicate the 1:10 (CSF) and 1:40 (serum) dilutions used to create IgG subclass profiles. “One-site specific binding” curve fitting based on the equation Y = Bmax*X/(Kd + X) was applied in Graphpad prism.

can be judged as quantitative. Consequently, we pursued a semiquantitative approach to analyze changes in antibody levels for Figure 2 and 3 and employed non-parametrical statistical tests when ΔMFI values were compared.

1.5 Investigation of the matrix effect

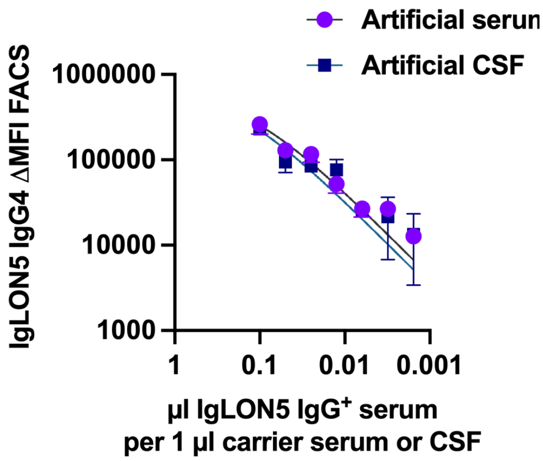

Supplementary Methods figure 7: Investigation of CSF and serum matrix effects on IgLON5 autoantibody binding. Dilution of IgLON5 IgG-positive serum from patient 8 in non-IgLON5 patient carrier CSF or serum to create artificial IgLON5 IgG-positive serum and CSF samples with defined IgLON5 antibody concentrations in different matrices. "One-site specific binding" curve fitting based on the equation Y = Bmax*X/(Kd + X) was applied in Graphpad prism. P= 0.227, two-tailed t-test.

For a reliable quantification of a CSF/serum antibody index, absence of a matrix effect is mandatory. To test whether the composition of the matrix surrounding the antibodies (serum or CSF) affects the measurement accuracy of the flowcytometry assay for IgLON5 antibodies, we spiked serum and CSF from a non-IgLON5 patient (carrier serum/CSF) with a highly anti-IgLON5 positive serum (patient 8) to create artificial serum and CSF samples with defined IgLON5 antibody concentrations from the same source with similar matrix conditions. These resulting artificial IgLON5 IgG-positive serum and CSF samples were then diluted 1:40 in blocking medium and measured (Supplementary Methods figure 7). At all anti-IgLON5 antibody levels, the matrix, either CSF or serum, did not affect the ΔMFI values in the flow cytometry assay.

1.6 Measurement of the CSF/serum IgLON5-IgG4 index

After showing that dilution to more than 1:400 for serum where necessary to obtain IgLON5 IgG4 quantification and that a matrix effect is neglectable, we proceeded to measure CSF/serum IgLON5-IgG4 antibody indices to test whether intrathecal synthesis (IS) of IgLON5 IgG occurs in patients with anti-IgLON5-disease. For this purpose, we re-analyzed the CSF/serum samples already analyzed for Figure 1-4, which were still available. In addition, laboratory data for the CSF/serum albumin (Q_{Alb}) and IgG ratios (Q_{IgG}) had to be available. We based our method on a technique described by Reiber and Lange (1). IgLON5 antibodies can be assumed to enter the CSF at a similar rate as total IgG. IgG4 has the same size as IgG in general. Thus, in the absence of IS the CSF/serum ratio of anti-IgLON5 IgG4 ($Q_{IgLON5\text{-}IgG4}$) should be identical to the total IgG CSF/serum ratio (Q_{IgG}). In case that anti-IgLON5 antibodies are synthesized intrathecally, the ratio of $Q_{IgLON5\text{-}IgG}$ to Q_{IgG}, the anti-IgLON5 CSF/serum antibody index (IgLON5-IgG4 AI), should be >1.

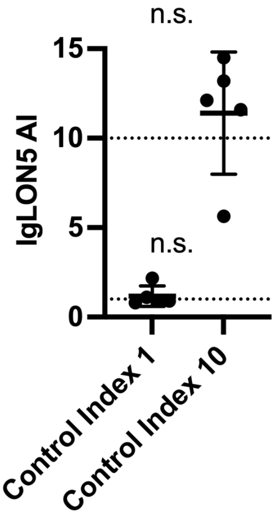

Supplementary Methods Figure 8: Validation of the IgLON5 IgG4 AI using artificial serum/CSF pairs generated from control serum spiked with anti-IgLON5 serum. Artificial AI=1 shows samples with an absence of IS, while Artificial AI=10 shows a sample with 10-fold IS (N=5). Both results were not significantly different from the expected AIs of 1 and 10 when tested by one sample t tests (p=0.5418 and 0.4099, respectively).

First, CSF was diluted 1:20 to ensure that the ΔMFI values were within the linear range of the assay, and then we diluted sera to similar IgG concentrations as the CSF according to the sample pairs' Q_{IgG} (to obtain $Q_{IgG} = 1$). In the absence of IS of IgLON5-IgG4, this should lead to identical ΔMFI values for IgLON5 IgG4 levels in CSF and serum.

Next, we divided the ΔMFI of CSF by the ΔMFI serum to obtain the $Q_{IgLON5\text{-}IgG}$, which was equivalent to the IgLON5-IgG4 AI as the samples were diluted to a Q_{IgG}=1. To validate the assay, we employed control CSF/serum pairs artificially spiked with anti-IgLON5 serum to obtain serum and CSF anti-IgLON5 concentration expected in the absence of IS, i.e. an IgLON5 IgG4 AI of 1.0 ("control index 1") and CSF anti-IgLON5 IgG4 concentration ten-fold higher than expected according to the serum concentration, i.e. an IgLON5 IgG4 AI of 10 ("control index 10"). Repeated measurements of "control index 1" and "control index 10" sample pairs resulted in an IgLON5 AI of 1.2 ± 0.6 (mean ± SD) and 11.4 ± 3.4, respectively (N=5). Both results were not significantly different from the expected AIs of 1 and 10 when tested by one sample t tests (p=0.5418 and 0.4099, respectively). The upper normal limit in the absence of IS (mean of "control index 1" ± SD), above which an IS can be assumed with high confidence, was 2.7 (Supplementary Methods Figure 8). When the data generated from by the repeat measurement of CSF and serum and the respective QAlb were used, 7 or 11 data point were clearly above the cut-off (Supplementary Methods Figure 9).

To analyze the IS of IgLON5-IgG4 independently of the actual Q_{IgG}, we converted to z scores. Here the denominator is not the actual Q_{IgG} but the mean expected Q_{IgG} at a the Q_{Alb} of the CSF/serum sample pair in the absence of IS of total IgG. To this end, the IgLON5 IgG4 AI, which is the CSF/serum IgG4 antibody ratio divided by the Q_{IgG}, has to be first multiplied by the Q_{IgG}. Based on a large cohort of normal CSF datasets, the z score can be calculated from the IgLON5-IgG4 AI using the following formula:

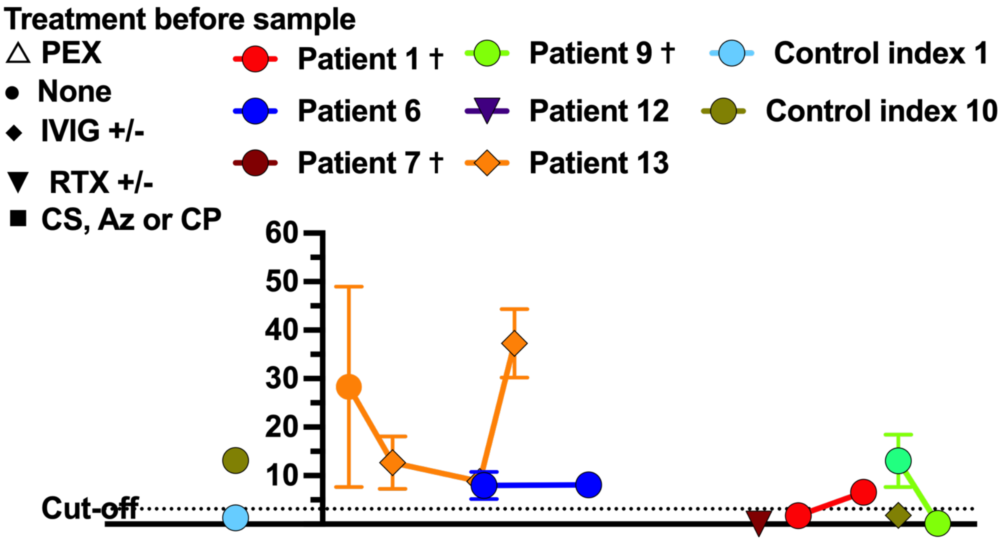

Supplementary Methods Figure 9: Intrathecal synthesis of anti-IgLON5 autoantibodies determined by the IgLON5 IgG4 antibody index (AI). IgLON5-IgG4 was quantified using flow cytometry in cerebrospinal fluid and serum using flow cytometry diluted to equivalent total IgG concentrations. Controls: artificial control samples without intrathecal synthesis (indicated by a 1:1 ratio IgLON5-IgG4 in liquor and serum, "control index 1") were generated and measured as control. The cut-off for intrathecal synthesis was determined as mean plus 3x standard deviation of the CSF/serum IgLON5 antibody index of the control index 1 patient and is indicated as dotted line. As positive control, artificial control samples with a tenfold intrathecal synthesis were generated and measured ("control index 10").

$$Formula\ 4{:}\ zIgLON5(IgG_4) = \frac{(\mathrm{LN}(Q_{IgG} * \mathrm{IgLON5}\ IgG_4\ \mathrm{AI}) - (0.9698 * \mathrm{LN}(Q_{Alb}) - 0.6863))}{0.114}$$

Similarly, Q_{IgG} can be used instead of "Q_{IgG}*IgLON5 IgG_4 AI" to calculate the z score for total IgG. The establishment of the z-score analysis is described in detail in Brauchle et al. (2)

References

1. Reiber H, Lange P. Quantification of virus-specific antibodies in cerebrospinal fluid and serum: sensitive and specific detection of antibody synthesis in brain. Clin Chem. 1991;37(7):1153-60.
2. Brauchle F, Rapp D, Senel M, Huss A, Dreyhaupt J, Klose V, et al. Clinical associations and characteristics of the polyspecific intrathecal immune response in elderly patients with non-multiple sclerosis chronic autoimmune-inflammatory neurological diseases – a retrospective cross-sectional study. Frontiers in neurology. 2023;14.
