# Supplementary material for: HLA dependency and possible clinical relevance of intrathecally synthesized anti-IgLON5 IgG4 in anti-IgLON5 disease
Source: Front Immunol. 2024 May 16;15:1376456. doi: 10.3389/fimmu.2024.1376456 (PMC11141242; doi:10.3389/fimmu.2024.1376456)
Supplement: Supplementary file 3 [file DataSheet_3.pdf]

Appendix: Clinical data

Patient 1

This 78-year-old female patient was diagnosed with anti-IgLON5 disease after having developed a slowly progressive dizziness and gait instability approximately 9 years earlier.

Within the last three years, her mood had become depressed and she reportedly had developed memory impairment and dyssomnia. Upon neurological examination, psychomotor slowing, sustained horizontal gaze-evoked nystagmus but no supranuclear gaze palsy were noted. There was mild dysarthria and she reported mild difficulties swallowing and fragmented sleep. In addition, she would fall asleep 3-5 times during the daytime. The gait instability and dizziness had considerably worsened so that she would fall repeatedly. Walking distance had shortened to 100 m. No bradykinesia or postural instability were noted. Her medical history revealed breast cancer diagnosed two years after onset of neurologic symptoms, which had been treated with bilateral mastectomy, and diabetes mellitus type II.

Cranial MRI showed moderate white matter changes compatible with cerebral microangiopathy. FDG PET showed glucose hypermetabolism of the basal ganglia. CSF had normal cell count (<4 leukocytes/µl, normal <5/µl), slightly increased protein (542 mg/L, upper normal limit 500 mg/L), CSF/serum albumin ratio (9.5 x 10^{-3}, upper normal limit 9.2 x 10^{-3}), and normal lactate levels (2.0 mmol/l, upper normal limit 2.6 mmol/L). Oligoclonal IgG was negative in CSF. IgLON5 IgG was positive in serum (1:12.800) and cerebrospinal fluid (CSF, 1: 128). HLA genotyping revealed that she was a heterozygous carrier of both the DRB1*10:01 and DQB1*05:01 alleles.

After diagnosis of anti-IgLON5 disease, treatment with intravenous immunoglobulins was initiated (2g/kg bodyweight per cycle). However, no improvement was noted. Fourteen months later, serum and CSF anti-IgLON5 titers were 1:6400 and 1:128, respectively. An intraductal papillary mucinous neoplasm (IPMN) of the pancreas was diagnosed and immunotherapy was discontinued. The patient was lost to follow-up and died one year later because of central hypopnea.

Retrospective analysis of the archived samples for anti-IgLON5 IgG subclasses showed IgG4 predominance in all samples tested. However, the last serum sample showed an 11-fold increase in anti-IgLON5 IgG3 levels compared to the previous serum, while CSF anti-IgLON5 IgG2 and IgG3 levels increased 15- and 40-fold, respectively. However, their contribution for the sum of all ΔMFI was still less than 1%. IgG1 in serum dropped by 23% in the second compared to the third sample, while later it remained stable. Serum IgG4 in serum didn't change

considerably (<10%), while IgG1 and IgG4 in CSF in the second compared to the first sampling increased 1.4- and 1.5-fold, respectively.

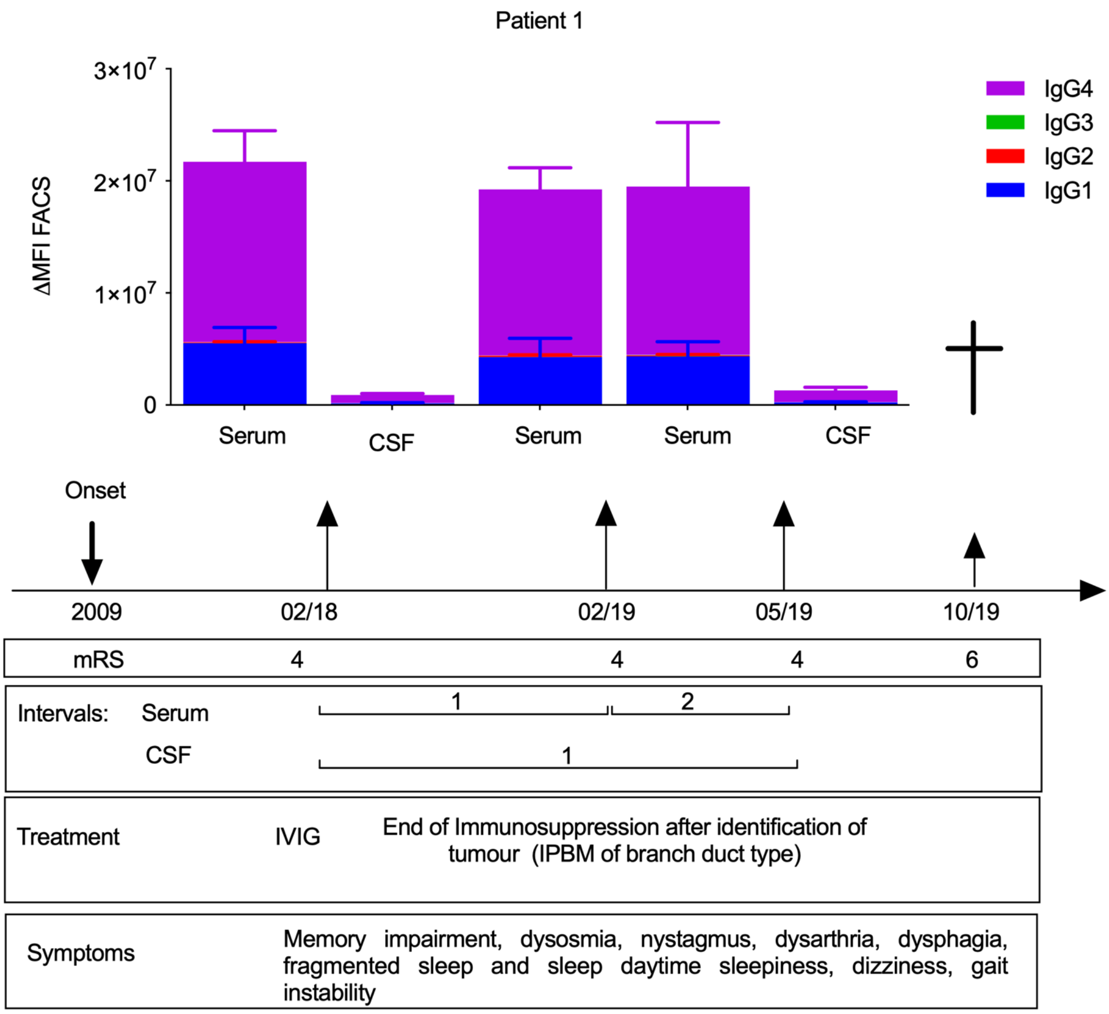

Patient 2

This case was reported in detail previously (1). In summary, the 54-year-old female presented with bilateral ptosis, her voice had become hoarse, she had dysarthrophonia and difficulties swallowing. She had a 1.5-year history of sleep disturbances comprising not only sleep onset and maintenance, but also daytime sleepiness with episodes of suddenly falling asleep, snoring as well as talking and arm and leg jerks during sleep. Video polysomnography performed before anti-IgLON5 disease was described demonstrated parasomnia with finalistic movements, vocalisations and stridor. In addition, a broad-based gait was noted.

Eleven years later, in 05/2014, she was diagnosed with anti-IgLON5 disease with positive anti-IgLON5-IgG in serum and CSF. HLA genotyping revealed that she carried the risk alleles DRB1*10:01 and DQB1*05:01. Four months later, she was admitted due to severe respiratory insufficiency because of stridor that required intubation (mRS 5). After initial treatment with plasmapheresis, high-dose intravenous methylprednisolone followed by oral tapering was administered. To treat respiratory insufficiency due to adduction of the vocal cords during expiration and lack of abduction during inspiration, she received a laterofixation of the left vocal cord in 11/2014. Although no artificial ventilation was needed following laterofixation, the patient was in a reduced general condition with relevant cognitive impairment, psychomotor slowing and unable to walk unassisted. As over time, there were no further signs of improvement, three months later treatment with plasmapheresis was repeated in 02/2015. However, she died due to aspiration pneumonia the following month.

Retrospective analysis of patient samples just prior to, during and following the second plasmapheresis demonstrated variable IgG4 predominance in serum with percentage of “total” IgG ΔMFI ranging from 60 to 72%, but 76% IgG4 in CSF. Only serum contained a low percentage of IgG2 (<3%), IgG3 was neither detected in serum nor in CSF. Following plasmapheresis serum IgG1 and IgG4 became 2.3- and 2.6-fold lower, respectively.

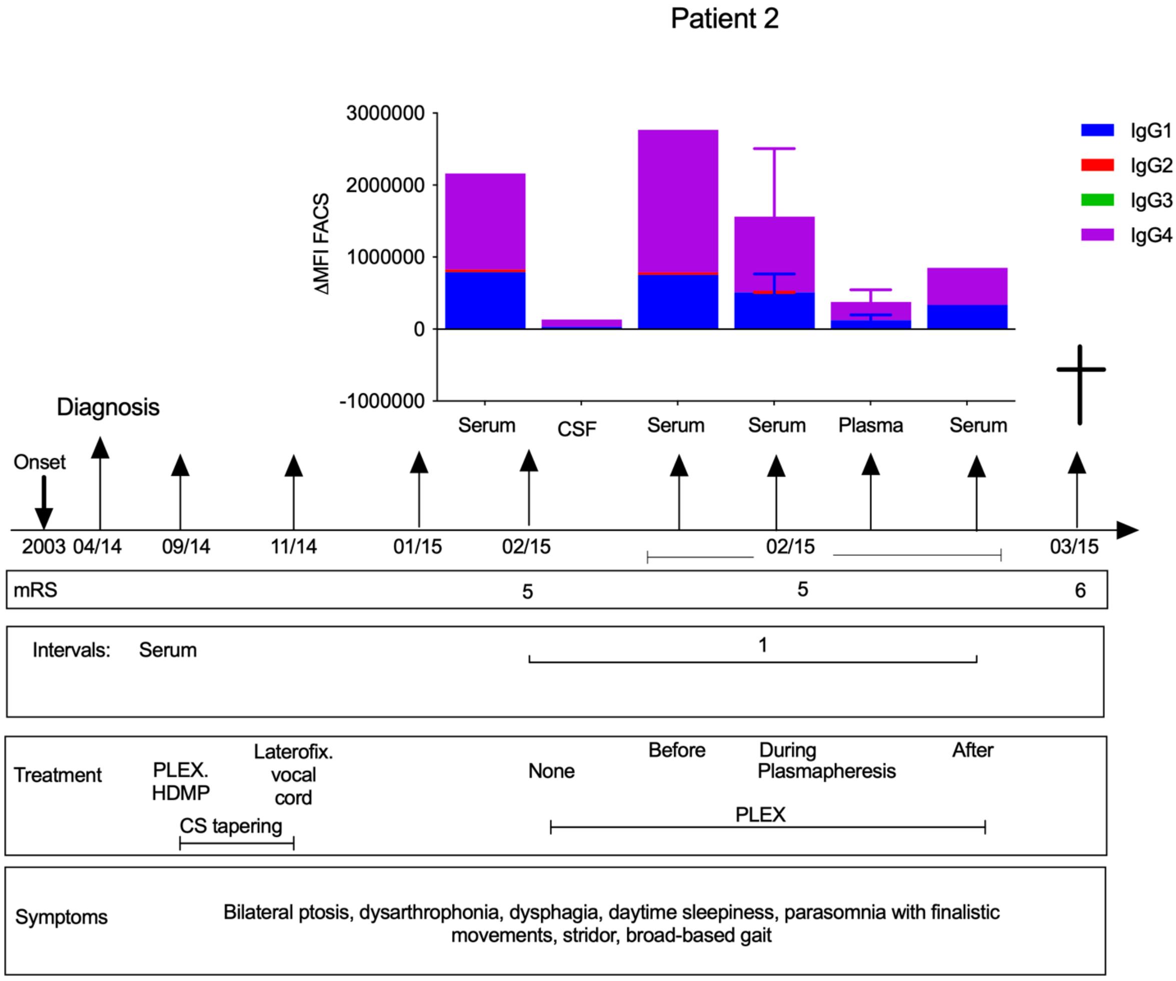
Patient 2
ΔMFI FACS
3000000
2000000
1000000
0
-1000000
Serum
CSF
Serum
Serum
Plasma
Serum
IgG1
IgG2
IgG3
IgG4
Diagnosis
Onset
2003
04/14
09/14
11/14
01/15
02/15
02/15
03/15
mRS
5
5
6
Intervals:
Serum
1
Treatment
PLEX.
HDMP
Laterofix.
vocal
cord
CS tapering
None
Before
During
Plasmapheresis
After
PLEX
Symptoms
Bilateral ptosis, dysarthrophonia, dysphagia, daytime sleepiness, parasomnia with finalistic movements, stridor, broad-based gait

Patient 3

The case of this 76-year-old male (age of onset 67 years), who died before anti-IgLON5 disease had been discovered, was published recently (2). Upon admission in 2012, he reported a 9-year history of symptoms: His mood had become depressed, and upon neurological examination, double vision, hypsometric and slow saccades were observed. In addition, mild dysarthrophonia was present. More recently, he had developed a hoarse voice and difficulties swallowing and started to aspirate solid food. He also reported snoring and insomnia, but neither vocalizations nor goal-directed behavior during sleep were reported. There were mild facial myokymias, mild tremor of both hands as well as limb ataxia. He reported imbalance with repeated falls. Gait was moderately broad-based and short-stepped with mild postural instability. He also reported nocturnal urge incontinence.

Brain MRI revealed temporomesial and midbrain atrophy as well as thinning of the corpus callosum. Dopamine transporter SPECT showed reduced striatal uptake, more prominent on the left. CSF leukocyte count was normal (1 leukocyte/µl), CSF protein slightly elevated (447 mg/l, UNL <430). Oligoclonal IgG in CSF was negative. Neuropsychological evaluation revealed mild cognitive impairment. In summary, PSP was suspected.

During his stay in hospital, episodes with severe stridor, especially during sleep, resulting in respiratory insufficiency became apparent. Thus, continuous CPAP ventilation was started during the night. Finally, he required tracheotomy. Four months later, he died from traumatic subdural hematoma after falling.

Five years after his death, archival CSF was analyzed and tested positive for IgLON5 antibodies. Retrospective HLA genotyping revealed that the patient did not carry the HLA DRB1*10:01, but the DQB1*05:01 risk allele.

Flow cytometrical quantification of anti-IgLON5 IgG subclasses in the CSF sample revealed IgG4 predominance with 80% of “total” MFI, the other 20% were IgG1.

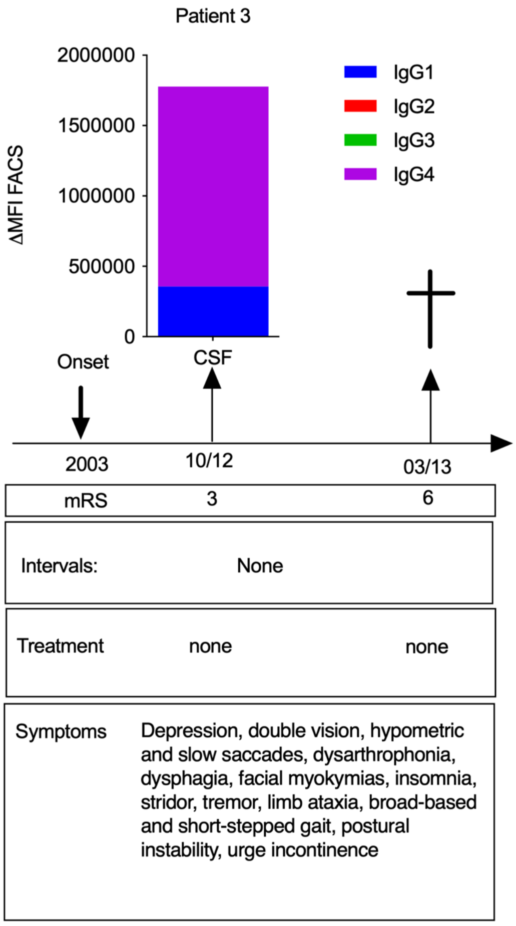

Patient 3
ΔMFI FACS
2000000
1500000
1000000
500000
0
CSF
IgG1
IgG2
IgG3
IgG4
Onset
2003
10/12
03/13
mRS
3
6
Intervals:
None
Treatment
none
none
Symptoms
Depression, double vision, hypometric and slow saccades, dysarthrophonia, dysphagia, facial myokymias, insomnia, stridor, tremor, limb ataxia, broad-based and short-stepped gait, postural instability, urge incontinence

Case 4

The 62-year-old male presented in 2009. Neurological examination revealed vertical supranuclear gaze palsy. He also reported insomnia and daytime sleepiness. There was mild hypo- and bradykinesia, and he reported progressive gait disturbances including unsteady gait but no rigidity. Gait was slightly bent forward with reduced arm swing on the left more than on the right side. There was mild postural instability. Brain MRI revealed mild microangiopathy. Dopamine transporter SPECT with [^{123}I]FP-CIT revealed striatal uptake more pronounced on the right. CSF examination showed a normal leukocyte count (1 cells/µl) and slightly elevated protein 520 mg/L. Oligoclonal bands in CSF were negative. Video polysomnography did not reveal any relevant abnormalities. Following first presentation, he remained stable for 2-3 years without immunosuppressive therapy. Then symptoms gradually became worse. Within the following 5 years, vertical gaze paresis progressed as did the gait disorder with recurrent falls. Neurological examination in July 2017 revealed saccadic horizontal pursuit, gaze-evoked nystagmus bilaterally, complete vertical supranuclear gaze palsy upwards, hypomimia and pronounced hypophonia, dysarthria, nuchal and axial rigor, hypo- and bradykinesia due to mild rigor of the extremities (left > right), exaggerated muscle reflexes bilaterally without positive pyramidal signs. There was no evidence of abnormalities in surface and depth sensitivity or cerebellar function. In particular, the patient had difficulty getting up from a sitting position. Gait was broad-based and atactic. Furthermore, moderate to severe postural instability was seen. Brain MRI revealed midbrain atrophy. Dopamine transporter revealed no significant change compared to the previous examination in 2009. An additional [^{18}F]fallypride PET/CT showed normal dopamine D2/3 receptor density bilaterally and, thus, there was no evidence for an atypical Parkinson's syndrome. L-Dopa test showed no benefit. A tilt table examination did not indicate orthostatic dysregulation.

In September 2017 daytime sleepiness as well as difficulties to concentrate had developed. Video polysomnography diagnosed obstructive sleep apnea and difficulty falling asleep/staying asleep. CSF examination in November 2017 showed normal leukocyte count (0 cells/µl), and normal protein (420 mg/L). Oligoclonal bands in CSF again were negative. However, anti-IgLON5 disease was diagnosed. HLA genotyping showed that the patient neither carried the DRB1*10:01 nor the DQB1*05:01 risk allele.

The patient remained largely stable for 1.5 years without immunosuppressive therapy (mRS3) and worsened by April 2019. The patient became unable to walk without help and developed a mild cognitive impairment (mRS4). He developed dysphagia and dysarthrophonia. A combined sleep breathing disorder with obstructive and central apneas with Cheyne-Stokes breathing was

diagnosed. He did not respond to HDMP administered later than the last serum sample analyzed in this study. He died 14 years after onset due to central respiratory failure within the context of anti-IgLON5 disease.

Flow-cytometric analysis of anti-IgLON5 IgG subclass levels showed IgG4 predominance in all serum samples (85-88% of "total" MFI), however in CSF the levels of all IgG subclasses were comparable (IgG1 and 2 23 % each, IgG3 19% and IgG4 34%). Total levels of anti-IgLON5 IgG fluctuated during the course of the disease with a more than 2-fold increase from September 2018 to April 2019 followed by a roughly equal decrease.

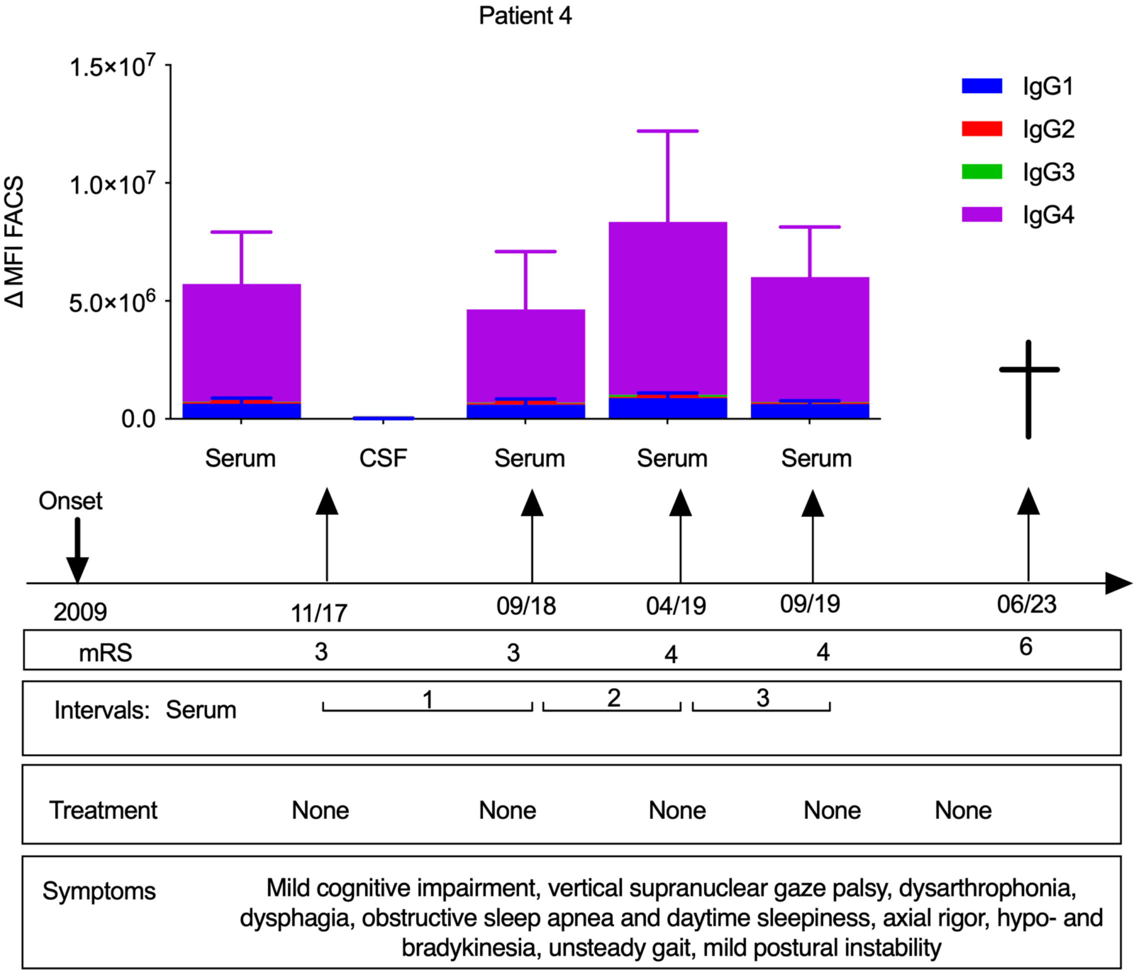

Patient 5

In August 2018, a 75-year-old female presented with a 6-month history of slurred speech, snoring, and unsteady stance and gait as an out-patient to the movement disorders clinic.

CSF was not investigated. IgLON5 IgG was positive in serum using routine cell-based assays (1:320, August 2018). Soon after diagnosis of anti-IgLON5 disease, she was admitted due to a minor right-hemispheric stroke with a left-sided hemiparesis.

Detailed neurological examination after recovery in 01/2019 demonstrated no oculomotor abnormalities, but dysarthria due to signs of glossopharyngeal and hypoglossal nerve lesions (deviation of the tongue to the left, limited elevation of the left soft palate).

Video polysomnography revealed severe obstructive sleep apnea with stridor and reduced sleep efficiency (72.3%; deep sleep phases 29.9%, REM phases 4.7%), but no vocalizations or goal-directed behavior.

Motor examination revealed slight central spastic hemiparesis on the left with exaggerated muscle jerks, probably as a residuum of the cerebral ischemia.

The gait was atactic, short-stepped broad-based and shuffling. Pull test showed moderate to severe postural instability.

There was no evidence of chorea, dystonia, rigidity/bradykinesia, autonomic dysfunction, or peripheral hyperexcitability.

MRI showed no relevant brainstem abnormalities; a small gliotic area in a right periventricular are was evident explaining the mild left-sided spastic hemiparesis.

HLA genotyping did not demonstrate carriership of the HLA risk alleles DRB1*10:01 und DQB1*05:01.

Clinically, she remained stable during 13 months without any immunosuppressive therapy. By June 2020, the unsteadiness of standing and walking gradually worsened and the patient developed a mild cognitive impairment (negative Amyloid-PET/CT). Afterwards she was lost to follow-up.

Flow cytometrical analysis of anti-IgLON5 IgG subclasses revealed IgG4 predominance (80% and 82% of "total" MFI, respectively), while IgG2 and IgG3 were less than 3%. "Total" Anti-IgLON5 MFI increased 1.8-fold from the first to the second serum without any relevant change in subclass distribution.

Patient 5

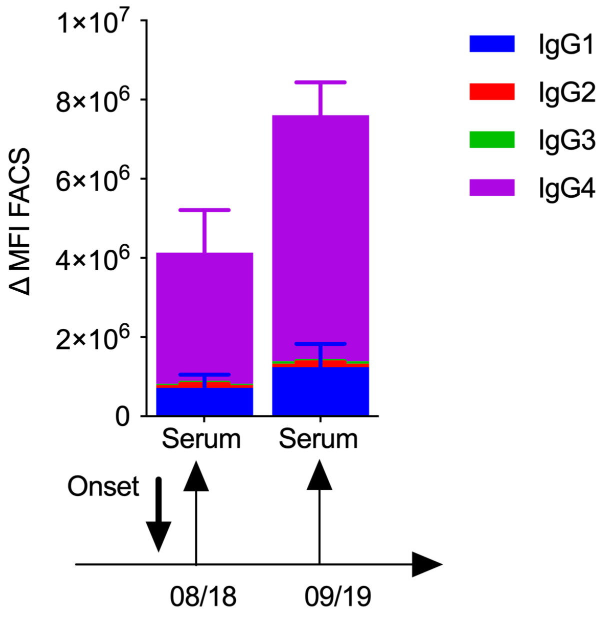

mRS	2	2
Intervals: Serum	1	

Treatment	None	None

Symptoms	Mild cognitive impairment, dysarthria, glossopharyngeal and hypoglossal nerve palsy, short-stepped gait, postural instability.

Patient 6

This 62-year-old female was admitted in 09/2013 due to an amnestic episode. In addition, reportedly depressive mood, personality changes and memory problems had developed within the last six months.

In the previous months, she had been admitted to another hospital following sudden and transient loss of consciousness, which had been diagnosed as syncope. Upon neurological examination, she first was disoriented but improved rapidly. Probably, the short-lasting disorientation represented postictal cognitive impairment. Still, memory problems persisted. No additional neurological abnormalities were observed.

Cerebral MRI revealed T2-hyperintensity and diffusion restriction of the right hippocampus. There was mild CSF pleocytosis (7 leukocytes/µl). Total protein was normal, oligoclonal IgG negative. With the suspected diagnosis of acute (limbic) encephalitis of either infectious or autoimmune origin with epileptic seizures and probably a prolonged postictal state, the patient was treated with acyclovir, and in 10/2013 IVIG and HDMP. PCR for HSV and VZV in CSF turned out to be negative. In addition, antiepileptic therapy with levetiracetam was started. Following immunotherapy, memory and mood improved, although residual memory deficits remained. Treatment was continued with monthly courses of IVIG in parallel to oral 75 mg prednisolone daily until 12/2013 followed tapering to 5 mg daily.

Over the disease course, her symptoms remained relatively stable while taking 5 mg prednisolone and receiving monthly IVIG. No more episodes suggestive of epileptic seizures were observed. Therefore, the antiepileptic therapy was discontinued. IVIG were also discontinued in 10/2014.

After a period of stable condition with 5 mg prednisolone only, the patient started to complain of hyperhidrosis. Repeat CSF analysis in 02/2015 revealed normal leukocyte count and again negative OCB. Now, testing for anti-IgLON5 IgG was performed, which was positive both in serum and CSF when using routine CBAs. HLA genotyping confirmed that the patient carried both HLA risk alleles DRB1*10:01 and DQB1*05:01. Video polysomnography could not detect parasomnia with goal-directed behavior typical for anti-IgLON5 disease but position-depending obstructive sleep apnea. Again, IVIG administered in regular intervals were added to 5 mg prednisolone. However, upon follow-up slowly progressive memory decline was observed. Thus, repeated CSF analysis was performed in 10/2015, which again showed normal cell count and negative OCB. Following LP, IVIG and 5 mg prednisolone were complemented with azathioprine in 10/2015. Still, worsening of the memory dysfunction continued. Thus,

IVIG were switched to rituximab in 03/2017, which was complemented with entecavir as occult HBV infection was present. Prednisolone was discontinued in 09/2017. In 10/2019 azathioprine was discontinued as adherence to oral medication became questionable. Following the last dose of rituximab, the patient did not re-appear to the agreed date for the next application, probably due to the cognitive impairment. As entecavir adherence became unlikely and she remained stable without immunosuppression, a wait-and-see strategy was viewed as most appropriate.

Testing the sera and CSF for anti-IgLON5 IgG subclasses using flow cytometry demonstrated IgG4 predominance in all sera but those from October 2014 (64% IgG4) and August 2022 (41% IgG4). In October 2014, this coincided with a relative increase of IgG1 from 21 to 35%, while from September 2020 to August 2022 the proportion of anti-IgLON5 IgG2 and IgG3 increased from less <0.5% to 22% and 8%, respectively, while anti-IgLON5 IgG1 increased only from 22 to 29%. CSF always showed IgG4 predominance (87-91%) with low relative IgG1 levels (8-13%) and very low IgG2/3. Levels of serum and CSF IgG4 remained relatively stable overtime until RTX treatment complemented azathioprine. In November 2018 compared to February 2017 serum anti-IgLON5 IgG4 had dropped by 52%, in September 2020 compared to November 2018 serum anti-IgLON5 IgG4 had again decreased by 48%. Correspondingly, CSF IgG4 in October 2019 war 4-fold lower than in February 2017. Discontinuation of immunotherapy in 2020 was associated with stable levels of serum anti-IgLON5 IgG4 while IgG1 increased 2.5-fold and IgG2/3 even more dramatically (92- and 519-fold).

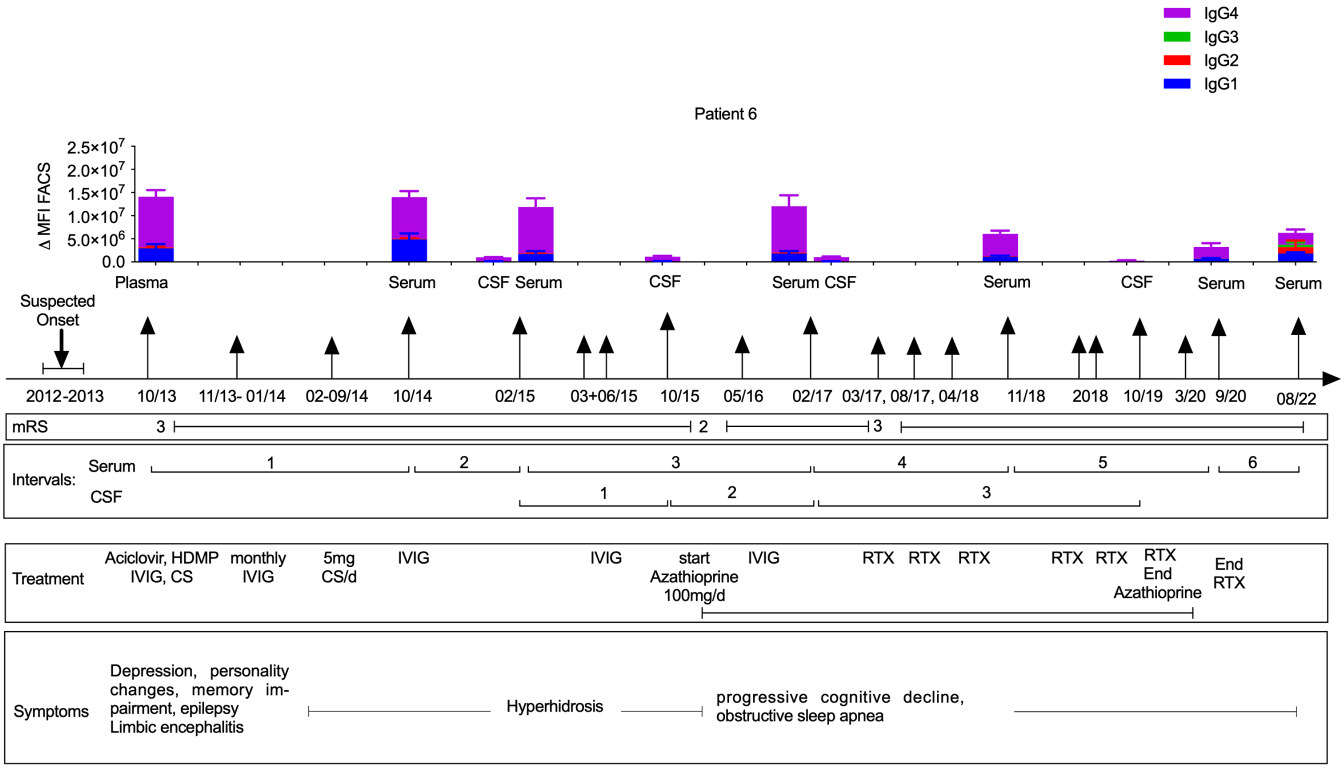

Patient 7

Following a 6-year history of progressive hoarseness, swallowing and breathing difficulties inluding nocturnal stridor, the 73-year-old female was admitted to the hospital in a comatose state due to respiratory failure and hypercapnia associated with pneumonia in 10/2017. According to her husband, she had been drowsy for several days before admission.

Respiratory insufficiency due to bilateral vocal cord palsy had already led to similar incidences since 2014. Repeated surgical expansion of the glottis had already been performed. CSF revealed normal cell count (3 cells/µl) and elevated total protein (507 mg/L). Oligoclonal bands were absent. The patient was treated with amoxicillin/clavulanic acid as pneumonia was detected.

After clinical stabilization, the patient was admitted to the department of otorhinolaryngology where she developed episodes of hypercapnia-association loss of consciousness that led to ICU treatment. Following artificial ventilation, she received tracheotomy and nightly CPAP ventilation.

Neurological examination following recovery from pneumonia revealed slight memory difficulties, horizontal gaze-evoked nystagmus, severe dysarthria and dysphagia. The patient reported restless sleep. No bradykinesia or gait disturbance were noted. Cerebral MRI showed transient pallidal T1 and T2 hyperintensity interpreted as post-ischemic/metabolic and cerebral microangiopathy. Repeat-CSF again showed normal cell count (2 leukocytes/µl) and normal total protein (426 mg/L). Oligoclonal IgG in CSF again was negative. Anti-IgLON5 CBA was positive both in serum (1:3200) and in CSF (1:16). HLA genotyping excluded carriership of the risk alleles DRB1*10:01 and DQB1*05:01.

As the patient recovered from the E. coli sepsis and pressure-controlled ventilation was successful, immunoadsorption and overlapping high-dose methylprednisolone 1000 mg over 5 days was started in 11/2017. As no further improvement was noted, the patient received two cycles of 375 mg/m^2 RTX in 01/2018. Immunotherapy was escalated to two cycles of cyclophosphamide in 01 and 03/2018. As no further improvement was noted, treatment was then continued with RTX maintenance therapy until 09/2021. In 2019 progressive depressed mood, cognitive decline, dysarthria and dysphagia were noted. In addition, another episode with respiratory insufficiency occurred. In addition, brief episodes of asymptomatic bradycardia (35 bpm) were noted. Cerebral MRI in 04/2019 showed mesiotemporal atrophy associated with slight global cerebral atrophy. CSF analysis in 06/2019 again showed normal cell count and protein (424 mg/l) and absent oligoclonal IgG. Anti-IgLON5 IgG tested by CBA was now 1:800 in serum and negative in CSF. RTX treatment was continued.

In December 2020, gait instability was noted, but the patient was still able to walk more than 1,000 m with walking aid and hundreds of meters when unassisted. During the following year, she lost her ability to walk unassisted due a multifactorial gait disorder including muscular disuse atrophy but also mild bradykinesia. In December 2021, the patient died in response to infection-associated respiratory failure.

Upon quantification of anti-IgLON5 subclasses, IgG4 remained the predominant subclass in all sera (78-84%), the proportion of IgG1 remained stable with 19 to 20% and IgG2 and IgG3 were either undetectable or very low. In CSF however, IgG4 predominance in the first sample (97%) switched to 50% IgG1 and 13 and 19% IgG2 and 3, respectively, while only 18% were IgG4. Following plasmapheresis, antibody levels dropped 2.5-fold in serum, but had already increased almost to levels prior treatment one month later. Following HMDP, RTX and cyclophosphamide levels decreased only by 16%, but more robustly later following continuous rituximab. Compared to the first CSF, the "total" anti-IgLON5 IgG was 2-fold lower. However, anti-IgLON5 IgG1 was 10-fold higher and IgG4 10-fold lower.

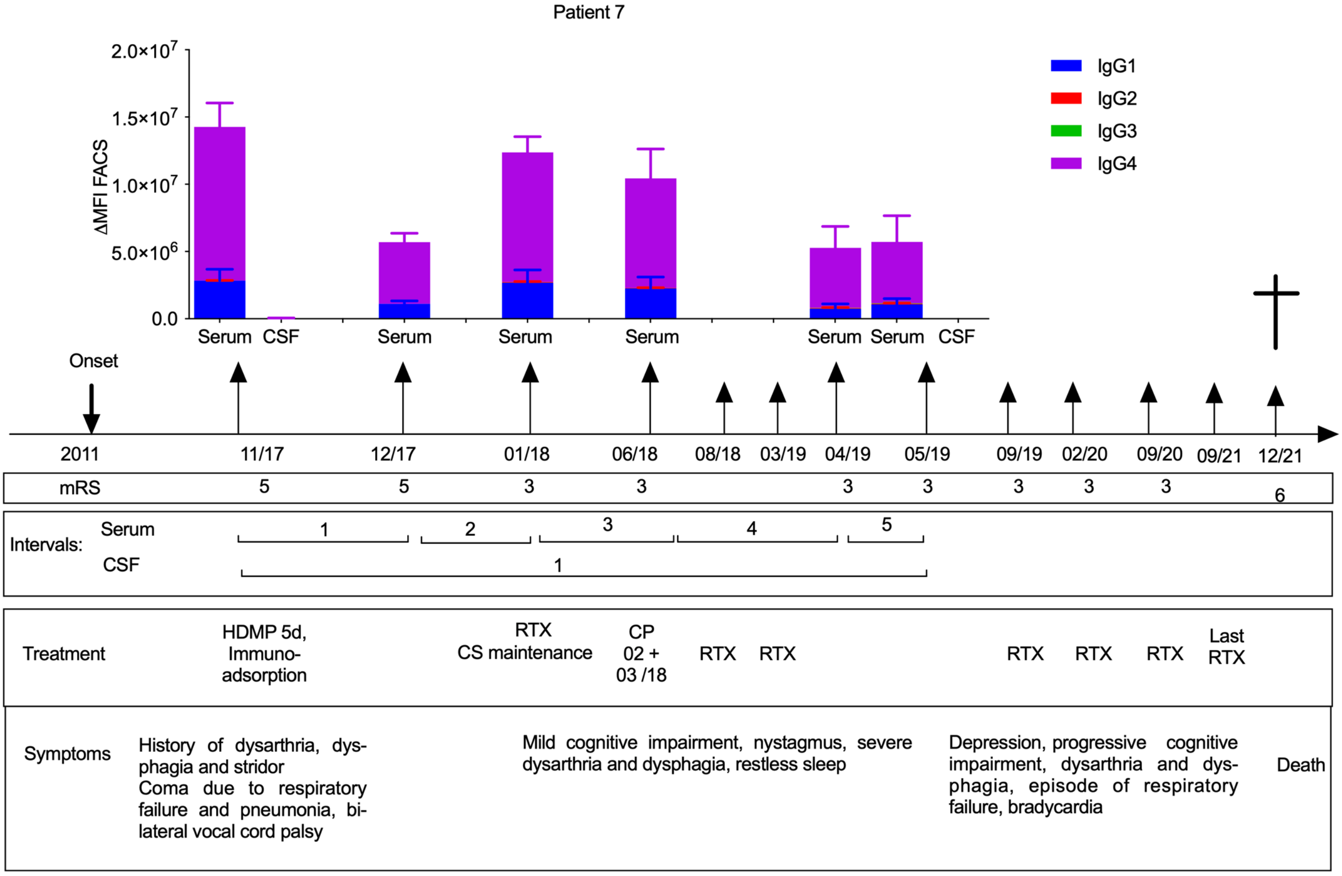

Case 8

In November 2016, a 60-year-old male presented with a two-year history of fasciculations, cramps and muscle stiffness in the lower limbs (initially of short duration and low frequency, over time persistent with reinforcement during physical exertion). In the 6 months before presentation, fasciculations also occurred in the arms, upper body and sometimes in the eyelid. In addition, muscle cramps developed in the lower legs during night. The patient also reported recurrent muscle pain with stiffness of both thighs and startle reaction. No other complaints were reported, especially no sleep disturbances, dysphagia or unsteadiness of gait.

Neurological examination revealed the reported fasciculations but no other neurological abnormalities.

Cerebral/spinal MRI and whole body FDG-PET/CT including brain showed no abnormalities. Analysis of CSF revealed mild pleocytosis (8 leukocytes/µl) and increased total protein (700 mg/L), oligoclonal IgG was negative. Onconeuronal, antiganglioside, myositis, thyroid, and GAD65 antibodies were negative. Testing for antineuronal surface antibodies, however, was positive for anti-IgLON5 IgG in both serum (titer 1:1600) and CSF (1:100). HLA genotyping was negative for DRB1*10:01 but positive for DQB1*05:01. Video polysomnography neither showed obstructive sleep apnea nor parasomnia typical for anti-IgLON5 disease.

Treatment with IVIG was initiated (6 cycles with 1 x 2g/kg and 5 x 1g/kg bodyweight; 6- to 8-week intervals for ten months), upon which the patient became symptom-free after five cycles. The patient remained symptom-free over a follow-up period of more than six years after stopping immunomodulatory treatment.

Testing the sera for anti-IgLON5 IgG subclasses using flow cytometry demonstrated IgG4 predominance with IgG4 being between 77 and 83% and IgG1 between 15 and 20% of all DeltaMFI. IgG2 and IgG3 were very low (2-3%). During the first 4 months of IVIG treatment, anti-IgLON5 IgG levels dropped 1.8-fold. One year after discontinuation of IVIG treatment, antibody levels had reached initial levels with an additional 1.2-fold increase 13 months later.

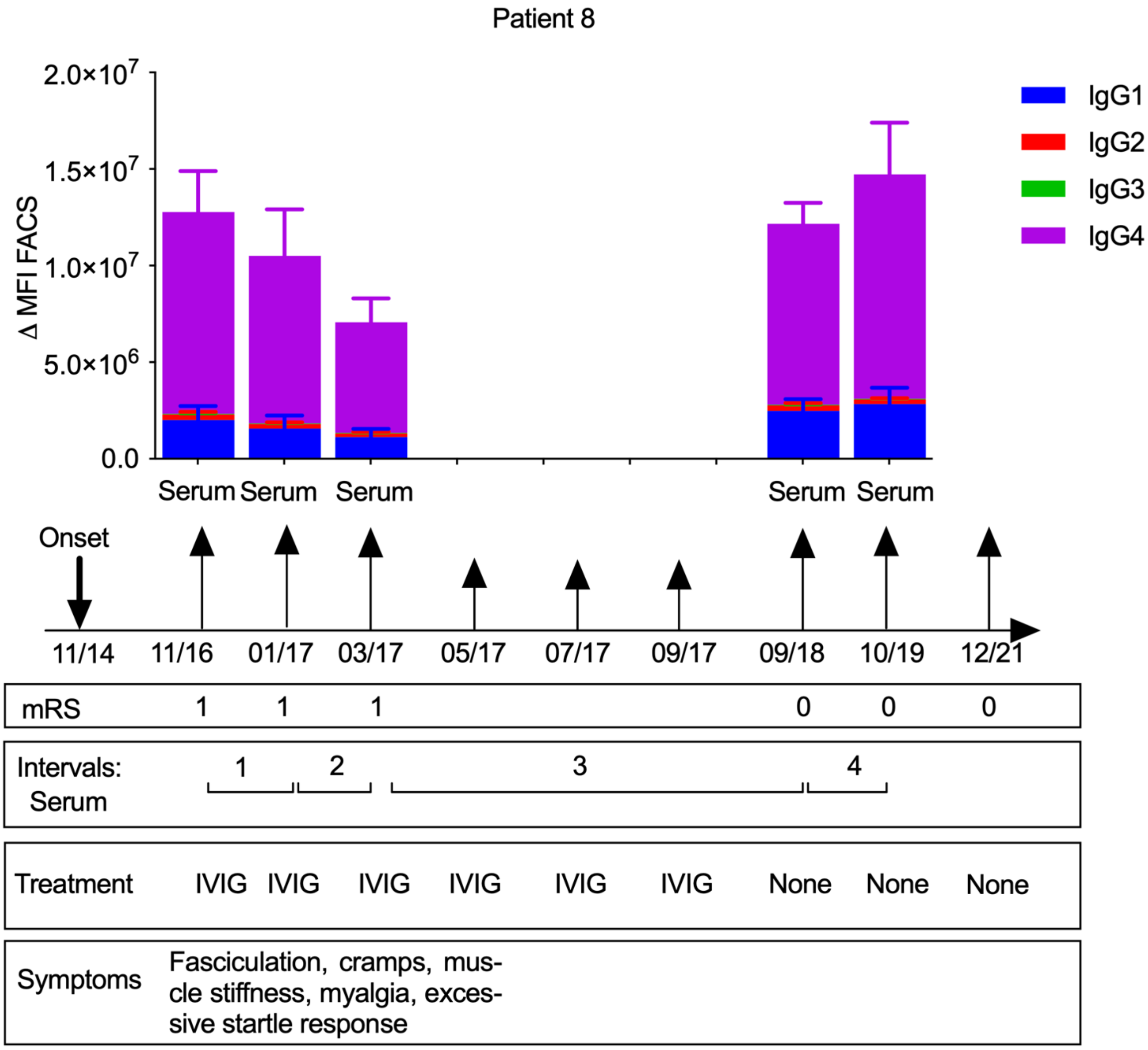
Patient 8
Δ MFI FACS
2.0×10^7
1.5×10^7
1.0×10^7
5.0×10^6
0.0
IgG1
IgG2
IgG3
IgG4
Serum Serum Serum Serum Serum
Onset
11/14 11/16 01/17 03/17 05/17 07/17 09/17 09/18 10/19 12/21
mRS 1 1 1 0 0 0
Intervals: Serum 1 2 3 4
Treatment IVIG IVIG IVIG IVIG IVIG IVIG None None None
Symptoms Fasciculation, cramps, muscle stiffness, myalgia, excessive startle response

Case 9

The case of this patient from Denmark has been published previously (3). The 61-year-old male was admitted to hospital in 01/2019 due to respiratory failure resulting in hypercapnia-induced loss of consciousness. A Mini Mental State Examination showed mild cognitive impairment (24/30) with visuospatial abnormalities. Upon neurological examination, left-sided ptosis as well as horizonal gaze palsy were noted. In addition, there was mild dysarthria and orofaciomandibular dystonia. Eleven years earlier, he had developed slowly progressive double vision, hoarseness and slurred speech and mild swallowing difficulties. Laryngoscopy showed bilateral vocal cord palsy.

One year before admission, he had developed respiratory problems and had been diagnosed with obstructive sleep apnea. Although CPAP ventilation had been started, he had been admitted to hospital several times within the previous year due to respiratory failure at night.

Within the last 6 months, he had experienced mild gait imbalance. In addition, his wife reported that movements during sleep and behavioral changes with disinhibition had developed. Upon examination, polysomnography showed stridor, vocalizations and abnormal movements during the rare instances of sleep (24 min per night only, no N3).

At presentation, generalized limb fasciculations were seen. There was mild spastic tetraparesis, pronounced in the right leg, here with the plantar reflex being extensor, as well as mild gait ataxia.

Shortly after onset, cerebral MRI had shown white matter hyperintensities in the brainstem, which had not progressed upon follow-up examinations. CSF analysis had shown mild pleocytosis (15 leukocytes/µl), normal protein levels and no OCB.

CSF analysis was normal. CBA was positive for anti-IgLON5 IgG in serum (1:1000) as well as CSF (1:10). HLA genotyping proved carriership of both risk alleles DRB1*10:01 and DQB1*05:01.

The patient received HDMP and plasma exchange. Oral prednisolone in decreasing doses was continued following HDMP. Although both the patient and his spouse reported that gait and sleep had become somewhat better, clinical examination did not find any relevant improvement. Thus, second-line treatment with rituximab was started in 02/2019. However, three weeks later, in 04/2019, another episode with respiratory failure, now during daytime, ensued. This time artificial ventilation had to be continued due to repeated hypercapnia during weaning. Thus, the patient received tracheotomy. At time of respiratory failure, anti-IgLON5 IgG had dropped to 1:100 in serum and 1:1 in CSF. Another cycle of plasma exchanges was performed, and prednisolone was increased to 75mg/d and again slowly tapered (25 mg in 06/2019, 10 mg in

10/2019). From September 2019 on, sleep improved (3 hrs and 58 min sleep in September). Prednisolone was finally discontinued in 02/2020. Further improvements were seen in May 2020 (N3 but no REM sleep and the patient presented with less abnormal movement). In addition, gait also improved. The patient still required respirator treatment at night, but not during the day. In October 2019, the symptoms included opthalmoplegia, ptosis, hypophonia and mild ataxia. The patient was considered stable and had been off corticosteroids since February 2020. He had become independent of mechanical ventilation at night, but still required a tracheostomy tube (mRS1). In June 2020, treatment with rituximab was continued with dosing every six months. However, symptoms progressed directly following rituximab administration until he stabilized one month later and then started to improve steadily. In September 2020, he was again able to ride a bike for the first time since >10 years. A split-night examination (CPAP only first part of the night) in February 2021 showed further improvement without apneas. Thus, in April 2021 the patient was decannulated. In March 2022, four months following the last dose of rituximab given in December 2021, gait ataxia began to worsen. He again received plasma exchange followed by oral prednisolone. In July 2022, he was re-admitted to the hospital with pneumonia associated with severe respiratory symptoms. He died following to hypoxic cardiac arrest. Autopsy confirmed Tau depositions compatible with anti-IgLON5 disease.
Quantification of anti-IgLON5 IgG subclasses in serum and CSF using flow cytometry showed IgG4 predominance of both serum (84%) and CSF (90%), with anti-IgLON5 IgG1 being the second most abundant subclass (15% and 9% in serum and CSF, respectively). Relative contribution of anti-IgLON5 IgG2 and IgG3 to total DeltaMFI in serum remained negligible (0.3% to 1.4%) with exception of the June 2019, where "total" anti-IgLON5 was lowest and relative anti-IgLON5 IgG2/3 peaked at 4.5%. In contrast, in CSF IgG4 predominance was lost upon initial treatment with plasma exchange, corticosteroids and one course of rituximab (IgG4 from 90% to 38%), associated with a prominent increase in IgG2 (0.4 to 27%) and IgG3 (0.3 to 20%). Still, the initial treatment drastically reduced "total" anti-IgLON5 IgG 6-fold in serum and 80-fold in CSF. Although serum "total" anti-IgLON5 IgG increased from April 2019 to October 2019 3.3-fold, with lower levels in June 2019, CSF levels decreased 1.1-fold. A further 1.7-fold increase in "total" anti-IgLON5 IgG levels was seen at the latest timepoint in June 2020.

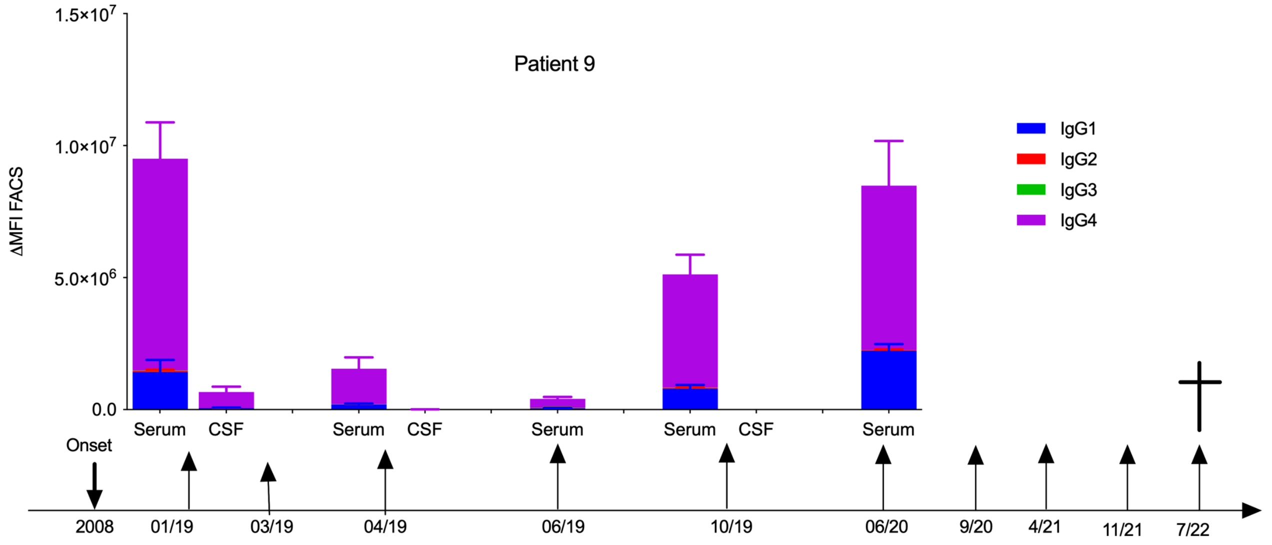

mRS	3	4	2	1	1	1	1	1	6

Intervals:
- Serum: 1, 2, 3, 4
- CSF: 1, 2

Treatment: PLEX + CS 5 days, then tap. | RTX | PLEX +Tracheost. + CS 25-75mg | 25 mg CS | 10 mg CS | RTX every 6 months | Removal tracheo-stoma

Symptoms

History of: disinhibition, behavioral changes, diplopia, dysarthrophonia, dysphagia, obstructive sleep apnea, episodes of respiratory failure, gait instability
Acute: coma due to respiratory failure
Upon recovery: Mild cognitive impair-ment, horizontal gaze palsy, ptosis, orofacial dystonia, parasomnia with finalistic behavior, stridor, fasciculations, gait ataxia, spastic tetraparesis

Prolonged res-piratory failure

improvement of sleep and gait (mild gait ataxia) Respirator treat-ment at night

Opthalmople gia, ptosis, hypophonia, mild ataxia

Stable,nights w/o respirator, tracheostomy.

Bicycle riding possible

Independent living at home Respirator treatment at night

pneumonia, respiratory failure Death

Case 10

This 58-year-old female reported low mood and problems concentrating. . Her voice had become low. She also reported sleeping difficulties at night with daytime sleepiness. She further reported a 4-year history of tremor and difficulties in walking. However, she had never experienced falls. The patient Treatment with L-dopa and dopamine agonists due to the diagnosis of Parkinson's disease had not improved her symptoms.

Upon neurological examination in 2018, the Mini Mental State Eamination (MMSE) was 30/30, MDS-UPDRS III was 20 and her oculomotor function was normal. Prominent hypokinetic dysarthria was observed. Her movements were bradykinetic and generalized fasciculations were observed. There was right-sided rigidity and resting tremor induced by contralateral activation. Gait was shuffling with some freezing at turning. However, postural reflexes were normal.

Cerebral MRI was normal. Dopamine transporter SPECT demonstrated reduced striatal uptake consistent with Parkinson's disease. Due to the generalized fasciculations anti-neuronal antibodies were screened and anti-IgLON5 tested positive in serum (Titer: 1:6400). HLA genotyping excluded carriership of the risk alleles DRB1*10:01 and DQB1*05:01. Immunotherapy with IVIG 2g/ kg bodyweight every 4 weeks for 3 months and azathioprine 150 mg daily were added to the dopaminergic medication with 3.15 mg pramipexol daily in 04/2020. Two months later, tremor as well as bradykinesia had improved to that extent that these symptoms did not lead to any functional impairment. IVIG was continued with 1g/kg bodyweight until 09/2022. IVIG was switched to subcutaneous immunoglobulins in 09/2022. At that time, L-dopa was added in a daily dose of 600 mg, azathioprine increased to 175 mg/daily. MDS-UPDRS III was 19. Azathioprine was increased to 200 mg in 11/2020. MDS-UPDRS III improved to 16 in 03/2021, 14 in 07/2021, and finally 10 in 11/2021. In 2022 the patient reported limb paresthesias. However, nerve conduction studies were normal.

Analysis of serum anti-IgLON5 IgG subclasses at time of diagnosis and after 15 months of immunotherapy both revealed IgG4 predominance (74% and 80%, respectively) with the remaining anti-IgLON5 almost exclusively IgG1 (0.5% IgG2/3 in 12/2019, no IgG2/3 detected in 02/2022). Upon immunotherapy, "total" anti-IgLON5 IgG decreased 3-fold.

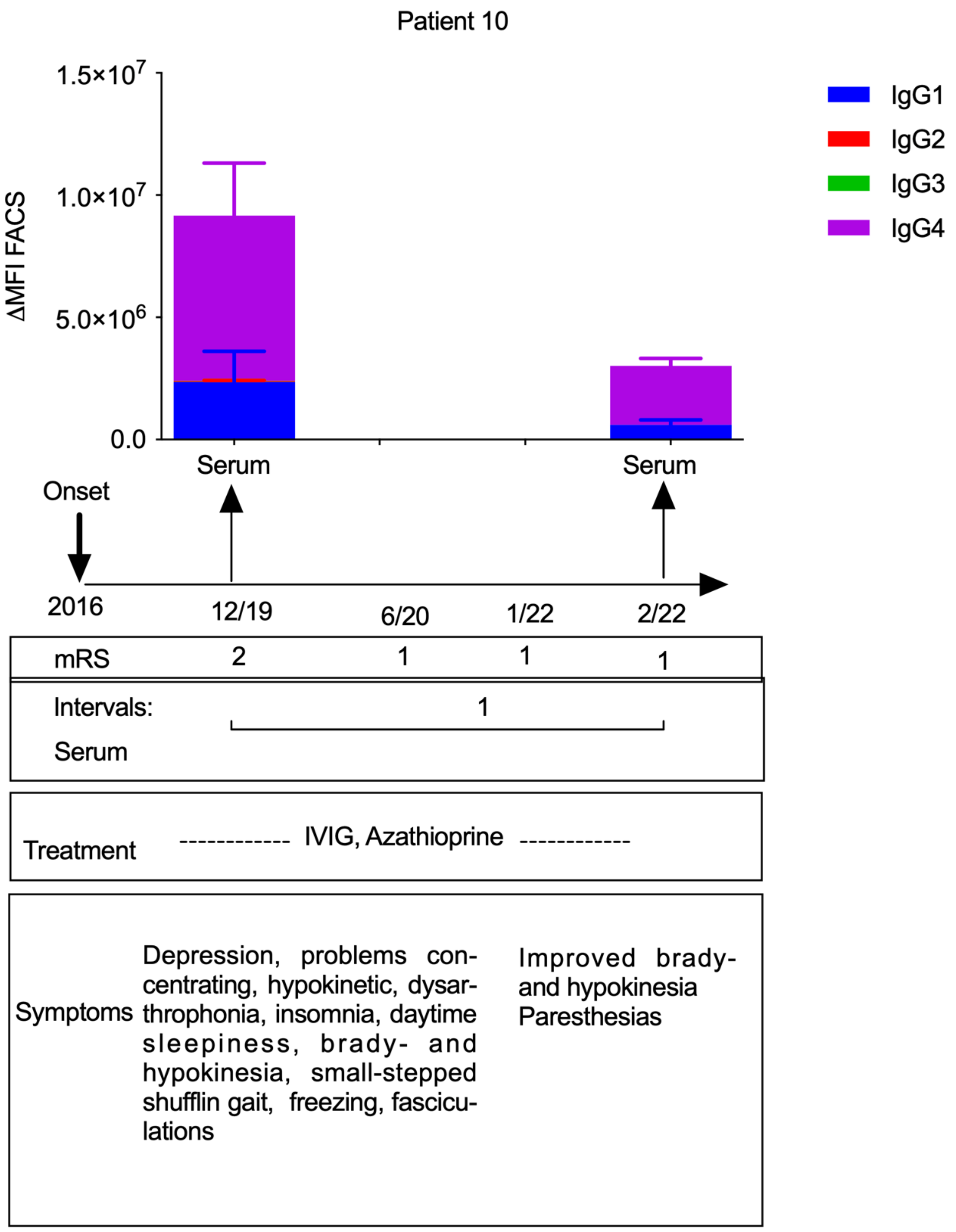
Patient 10
1.5×10^7
1.0×10^7
5.0×10^6
0.0
ΔMFI FACS
IgG1
IgG2
IgG3
IgG4
Serum
Serum
Onset
2016
12/19
6/20
1/22
2/22
mRS
2
1
1
1
Intervals:
Serum
1
Treatment
------------ IVIG, Azathioprine ------------
Symptoms
Depression, problems con-centrating, hypokinetic, dysar-throphonia, insomnia, daytime sleepiness, brady- and hypokinesia, small-stepped shufflin gait, freezing, fascicu-lations
Improved brady- and hypokinesia Paresthesias

Case 11

The 78-year-old male with a 3-year history of memory difficulties. Upon neurological examination, no oculomotor abnormalities were seen. He had developed sleeping difficulties with vivid dreams, but no purposeful behavior during sleep were reported. He was admitted due to involuntary worm-like movements of this right arm that had developed 5 days before. Examination of the lower cranial nerves was normal. Muscle jerks were slightly exaggerated bilaterally. He reported a 3-year history of slight unsteadiness of gait and upon examination the gait was atactic. For quite some time he suffered from an itching scalp, nocturnal burning feet, and calf cramps.

Cerebral MRI revealed mild microangiopathy, while absolute spinal stenosis on the level C5/6 was seen in spinal MRI, however without myelopathic signal alterations. CSF analysis revealed normal cell count (1 leukocyte/µl) and normal protein. Oligoclonal IgG in CSF was not tested. IgLON5 antibodies tested positive in CBA for serum (1:400) and negative in CSF. HLA genotyping excluded carriership for the risk alleles DRB1*10:01 and DQB1*05:01.

The hyperkinetic movements of his right arm receded spontaneously. All other complaints presented as stable at follow-up examinations 11 months later. No immunotherapy was started. Testing of serum and CSF obtained at the timepoint of diagnosis by flow cytometry to quantify anti-IgLON5 IgG subclass concentrations revealed IgG4 predominance in serum (78%) but not in CSF (39%). Relative IgG1 in CSF was two-fold higher than in serum (38% vs. 19%, respectively). While relative contribution of anti-IgLON5 IgG2 in both serum and CSF was about 2%, the proportion of anti-IgLON5 IgG3 in CSF was 40-fold higher in CSF compared to serum (20% vs. 0.5%).

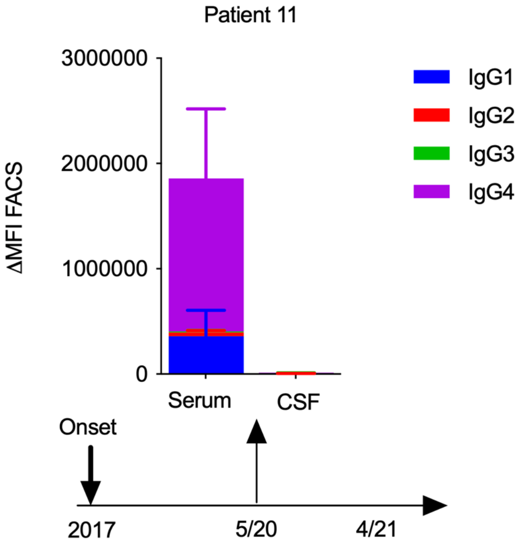

mRS	2	2
Intervals:	None	
Treatment	None	None
Symptoms	Mild cognitive impairment, sleep difficulties, transient choreoathetosis right arm, unsteady gait, fascicu-lations, calf cramps, itching scalp, nocturnal burning feet	

Case 12.

This 76-year-old male presented in 2019 with a 10-year history of depressed mood, impaired cognition, progressive horizontal and vertical gaze palsy, and neurological examination in 2019 showed supranuclear gaze palsy and glottic stenosis. He also reported a history of jaw dystonia and neurological examination showed severe jaw opening dystonia.He further reported fragmented sleep. The examination also showed mild bradykinesia, slightly more pronounced on the left, with mild action tremor.

Due to cardiac insufficiency (heart failure with preserved ejection fraction, HFpEF), the patient had been diagnosed with cardiac wild-type transthyretin amyloidosis by endomyocardial biopsy in 2018. A cardiac pacemaker had been implanted in 2019 for III° atrioventricular block. Probably as part of the amyloidosis, he had a pre-existing sensorimotor axonal polyneuropathy. In the first neurological examination in 2009, atypical Parkinson's disease had been suspected. Correspondingly, dopamine transporter SPECT had demonstrated reduced uptake in the left striatum. However, dopaminergic therapy had not resulted in any beneficial response. Follow-up SPECT in 2017 had shown progression with now bilateral reduction in dopamine transporter expression. Midbrain atrophy was revealed with otherwise unremarkable cerebral MRI in 2018. Previous medical treatments included systemic levodopa and local botulinum toxin with no effects.

Anti-IgLON5 antibodies were found in serum in 2019. Cerebrospinal fluid analysis revealed a normal cell count and no oligoclonal bands. HLA genotyping could not confirm carriership of the two risk alleles DRB1*10:01 and DQB1*05:01.

After the diagnosis of anti-IgLON5 disease, immunomodulatory treatment with IVIG was started. In addition, tracheostomy was performed. After for 4 months with no change in his neurological condition, it was decided to discontinue IVIG due to his cardiac insufficiency. Percutaneous endoscopic gastrostomy was performed in 2020. CSF analysis in 01/2020 revealed 1 leukocytes/µl. OCB were negative.

Upon flow-cytometrical analysis of the serum obtained in 10/2019 and CSF and serum from 01/2020 to quantify anti-IgLON5 IgG subclasses, IgG4 predominance was found in both serum samples (84% and 90%, respectively) and in CSF (84%). Upon treatment with IVIG, "total" anti-IgLON5 IgG did not change. However, serum IgG1 decreased 1.7-fold.

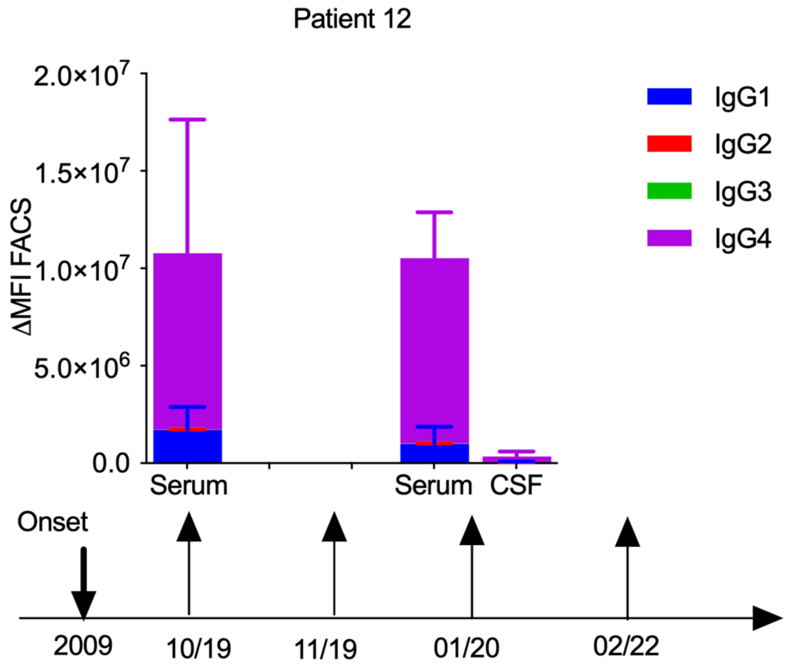

mRS	3		3	3
Treatment	None	IVIG	IVIG	None
Intervals: Serum	1			
Symptoms	Depression, mild cognitive impairment, supranuclear gaze palsy, glottic stenosis, jaw opening dystonia, fragmented sleep, mild bradykinesia, action tremor			

Case 13

This case has been previously published (4, 5). In June 2019, this 70-year-old male presented with the history of double vision starting 6 months earlier and neurological examination revealed double vision when oculomotor function was tested. The patient reported generalized muscle twitching, and generalized fasciculations and a slightly unsteady gait were observed. Electromyography in another hospital had shown acute and chronic neurogenic changes in several muscles. The patient also reported to have developed an awkward feeling in his left arm and moderate fatigue.

Cerebral MRI had been normal. CSF had shown pleocytosis (33 leukocytes/µl) and intrathecal IgG synthesis

Cerebral MRI now showed a prominent, non-enhancing, probably inflammatory lesion, in the right striatum. Electromyography showed generalized fasciculations. CSF (CSF 1, 06/19) again exhibited pleocytosis (56 leukocytes/µl), Q_{Alb} was increased to $10.3x10^{-3}$ (upper normal limit 8.7×10^{-3}), quantitative intrathecal immunoglobulin synthesis could not be demonstrated neither for IgG, IgA nor for IgM. Oligoclonal IgG was positive in the CSF. IgLON5 IgG was present in serum and CSF, in both with titers of 1:1,000 (Euroimmun cell-based assay). As latent tuberculosis was detected, the patient received IVIG, 2g per kg bodyweight, not corticosteroids. One month later, the patient was re-admitted with double vision and gait instability, which both had improved for 4 weeks following IVIG, but then had relapsed and worsened. CSF again showed pleocytosis (28 leukocytes/µl), blood-CSF barrier dysfunction (Q_{Alb} $11.3x10^{-3}$) and positive oligoclonal bands. IgLON5 IgG titer in serum was 1:1000, in CSF 1:100. HLA genotyping revealed carriership for the DRB1*10:01 but not for the DQB1*05:01 risk allele. Thus, in 08/2019 the patient received HDMP (1g for 5 days) followed by 100 mg of oral prednisolone daily and slow tapering. In addition, he received oral rifampicin for 9 months as tuberculosis prophylaxis.

After another transient improvement, the patient again developed double vision at an oral dose of prednisolone of 80 mg. In addition, muscle cramps in his hands, unsteady gait, dizziness, and a weak feeling in his legs had developed. Upon neurological examination, square wave jerks and generalized fasciculations, action tremor of both hands in addition to impaired balance were observed. Thus, in 09/2019 he again received combined IVIG (2g per kg bodyweight) and HDMP (1g for 5 days) followed by oral prednisolone. IVIG repeated every four weeks. In addition, azathioprine was started at a dose of 150 mg (2 mg/kg bodyweight) in 11/2019. The patient continuously improved under this regimen.

In 03/2020, the patient reported that at a prednisolone dose of 10 mg in parallel to azathioprine and monthly IVIG his gait again had become unsteady. In the meantime, polysomnography had revealed obstructive sleep apnea. In addition to fasciculations and hand tremor, mild paresis of elbow flexion bilaterally and finger flexion and extension of the right hand were observed. CSF (CSF 2, 04/20) showed 7 leukocytes/µl, Q_{Alb} was slightly increased (9.4×10^{-3}), oligoclonal IgG was now negative. IgLON5 IgG in serum was 1:1000, in CSF 1:100). Oral prednisolone was again increased to 50 mg, azathioprine to 175 mg and IVIG were given every 3 weeks. Under this regimen, the patients slowly improved, and prednisolone could be tapered to 2.5 mg without relapse in 09/2020 and discontinued one month later. However, tremor and fasciculations remained present. After improvement followed by stable disease, IVIG frequency was reduced to every four weeks in 08/2021. In 12/2021 and still stable disease, CSF (CSF 3) showed normal cell count (4 leukocytes/µl), normal blood-CSF barrier function (8.6×10^{-3}). Oligoclonal IgG was negative. IgLON5 IgG titer was 1:1000 in serum, 1:10 in CSF. Azathioprine was discontinued After another six months of 4-weekly IVIG and stable residual disease with slight tremor and fasciculations, IVIG frequency was reduced to every 5 weeks in 06/2022. In 08/2022 the patient again developed persistent double vision. CSF (CSF 4, 08/22) showed mild pleocytosis (7 leukocytes/µl), Q_{Alb} was normal (8.2×10^{-3}), oligoclonal IgG was negative. IgLON5 IgG titer in serum was increased to 1:10,000, in CSF to 1:100. The patient again received HDMP followed by oral tapering and IVIG frequency was again increased to every 4 weeks. Under this regimen, double vision increasingly improved.

Flow-cytometrical quantification of anti-IgLON5 IgG subclasses in the initial serum and CSF showed almost equal proportions of anti-IgLON5 IgG1 and IgG4 (serum: 39% vs 59%, CSF: 48% vs 50% respectively). Anti-IgLON5 IgG2/3 was always below 3% with the exception of December 2021, when anti-IgLON5 IgG4 levels were lowest, it peaked by 3.6% serum but 7% in CSF. During increasingly intensive immunosuppression, both serum and CSF IgG4 levels steadily declined from June 2019 to April 2020 and finally December 2021 (compared to initial levels: serum 3.8- and 8.1-fold, CSF 3.2- and 23.4-fold, respectively). However, in December 2021 compared to April 2020 serum anti-IgLON5 IgG1 increased 1.8-fold, and CSF anti-IgLON5 IgG1 dropped not as much as IgG4 did. This led to 63% and 74% anti-IgLON5 IgG1 in serum and CSF at the nadir of “total” anti-IgLON5 IgG. Following IVIG monotherapy with reduced frequency in August 2021, anti-IgLON5 IgG4 increase 1.3-fold in serum but 9.3-fold in CSF. In parallel, anti-IgLON5 IgG1 increase 2.1- and 3.4-fold in serum and CSF, respectively.

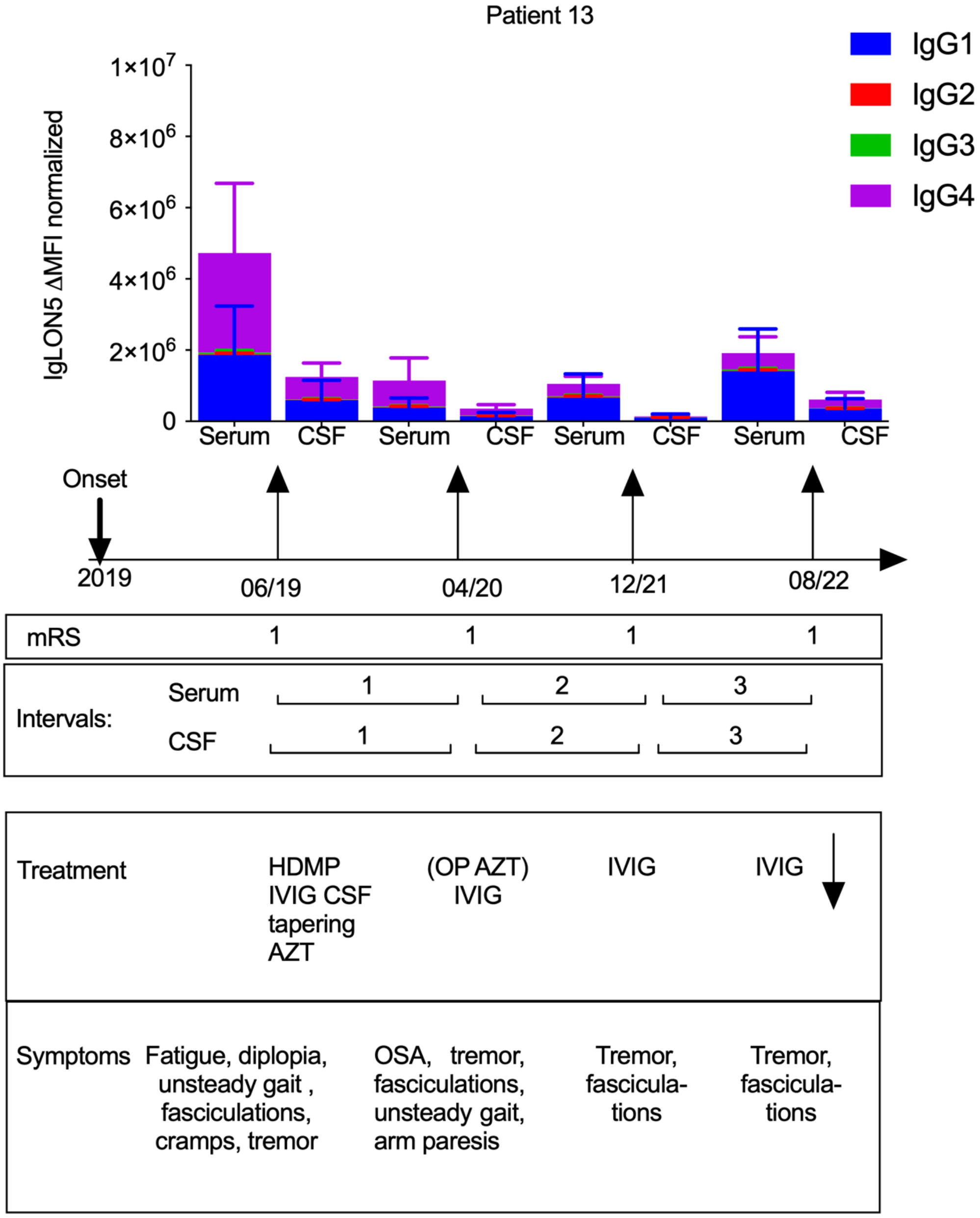

1. Hogl B, Heidbreder A, Santamaria J, Graus F, Poewe W. IgLON5 autoimmunity and abnormal behaviours during sleep. Lancet. 2015;385(9977):1590.
2. Berger-Sieczkowski E, Endmayr V, Haider C, Ricken G, Jauk P, Macher S, et al. Analysis of inflammatory markers and tau deposits in an autopsy series of nine patients with anti-IgLON5 disease. Acta Neuropathol. 2023;146(4):631-45.
3. Nissen MS, Blaabjerg M. Anti-IgLON5 Disease: A Case With 11-Year Clinical Course and Review of the Literature. Frontiers in neurology. 2019;10:1056.
4. Gaig C, Compta Y, Heidbreder A, Marti MJ, Titulaer MJ, Crijnen Y, et al. Frequency and Characterization of Movement Disorders in Anti-IgLON5 Disease. Neurology. 2021;97(14):e1367-81.
5. Gruter T, Mollers FE, Tietz A, Dargvainiene J, Melzer N, Heidbreder A, et al. Clinical, serological and genetic predictors of response to immunotherapy in anti-IgLON5 disease. Brain. 2022.
